# A proteomic approach reveals possible molecular mechanisms and roles for endosymbiotic bacteria in begomovirus transmission by whiteflies

**DOI:** 10.1093/gigascience/giaa124

**Published:** 2020-11-13

**Authors:** Adi Kliot, Richard S Johnson, Michael J MacCoss, Svetlana Kontsedalov, Galina Lebedev, Henryk Czosnek, Michelle Heck, Murad Ghanim

**Affiliations:** Department of Entomology, The Volcani Center, HaMacabim Rd., Rishon LeZion, 50250, Israel; Institute of Plant Sciences and Genetics in Agriculture, Robert H. Smith Faculty of Agriculture, Food and Environment, Hebrew University of Jerusalem, Rehovot, Israel; Genomic Pipelines, Earlham Institute, Colney lane, Norwich, NR7 4UH, UK; Department of Genome Sciences, University of Washington, Foege Building, 98195-5065 Seattle, USA; Department of Genome Sciences, University of Washington, Foege Building, 98195-5065 Seattle, USA; Department of Entomology, The Volcani Center, HaMacabim Rd., Rishon LeZion, 50250, Israel; Department of Entomology, The Volcani Center, HaMacabim Rd., Rishon LeZion, 50250, Israel; Institute of Plant Sciences and Genetics in Agriculture, Robert H. Smith Faculty of Agriculture, Food and Environment, Hebrew University of Jerusalem, Rehovot, Israel; USDA-Agricultural Research Service, Boyce Thompson Institute for Plant Research, Department of Plant Pathology and Plant-Microbe Biology, Cornell University, Ithaca, NY, USA; Department of Entomology, The Volcani Center, HaMacabim Rd., Rishon LeZion, 50250, Israel

**Keywords:** Bemisia tabaci, proteome, TYLC, transmission, bacterial symbiont

## Abstract

**Background:**

Many plant viruses are vector-borne and depend on arthropods for transmission between host plants. Begomoviruses, the largest, most damaging and emerging group of plant viruses, infect hundreds of plant species, and new virus species of the group are discovered each year. Begomoviruses are transmitted by members of the whitefly *Bemisia tabaci* species complex in a persistent-circulative manner. *Tomato yellow leaf curl virus* (TYLCV) is one of the most devastating begomoviruses worldwide and causes major losses in tomato crops, as well as in many agriculturally important plant species. Different *B. tabaci* populations vary in their virus transmission abilities; however, the causes for these variations are attributed among others to genetic differences among vector populations, as well as to differences in the bacterial symbionts housed within *B. tabaci*.

**Results:**

Here, we performed discovery proteomic analyses in 9 whitefly populations from both Middle East Asia Minor I (MEAM1, formerly known as B biotype) and Mediterranean (MED, formerly known as Q biotype) species. We analysed our proteomic results on the basis of the different TYLCV transmission abilities of the various populations included in the study. The results provide the first comprehensive list of candidate insect and bacterial symbiont (mainly *Rickettsia)* proteins associated with virus transmission.

**Conclusions:**

Our data demonstrate that the proteomic signatures of better vector populations differ considerably when compared with less efficient vector populations in the 2 whitefly species tested in this study. While MEAM1 efficient vector populations have a more lenient immune system, the Q efficient vector populations have higher abundance of proteins possibly implicated in virus passage through cells. Both species show a strong link of the facultative symbiont *Rickettsia* to virus transmission.

## Data Description

The whitefly *Bemisia tabaci* is a serious threat to worldwide agriculture, yet to our knowledge an extensive analysis of its proteomic profile has not been performed before. The data that we collected in this study represents the most extensive proteomic dataset available for this insect pest, or any hemipteran insect. We extracted total proteins for whole insects that were pooled from various populations and 2 different sub-species, digested them to peptides, and ran them on a mass spectrometer. Three biological replicates were collected per population and 3 technical replicates were run at random order per biological replicate. Data are available through ProteomeXchange with identifier PXD016964 and will be a valuable tool for future research of *B. tabaci* proteins involved in virus transmission and for further proteomic studies in insects.

## Potential Implications

The data provided here represent the first large-scale discovery proteomics dataset created for *B. tabaci* Middle East Asia Minor I (MEAM1, formerly known as B biotype) and Mediterranean (MED, formerly known as Q biotype) species, both worldwide pests of extreme economic importance. These data were used to mine different protein abundance patterns correlated with virus transmission ability. The 9 populations used in this study harbour different bacterial symbionts and have varying levels of resistance to insecticides. This dataset and the identified protein patterns provide a basis to study other differences at the protein level. The dataset was searched against hundreds of thousands of available whitefly sequences in the public databases; however, they were not searched against the published MEAM1 and MED genomes because those exhibited tremendous differences at the assembly level and have yet to be well annotated. We thus preferred to compare the dataset that we generated against available whitefly datasets, and with other insect species for which better genome sequences are available. In the future, the dataset provided here may be searched against the assembled genomes of both studied species.

## Background

Since first described >100 years ago, the whitefly *B. tabaci* has become an agricultural pest distributed on a worldwide scale. Its importance stems from its extreme invasiveness with international commodity trade, rapidly occupying new niches and displacing local populations, and it is now considered one of the most invasive species worldwide. *B. tabaci* causes direct cosmetic damage to various crops during feeding, and by the attraction of sooty mold fungus to its sugar-rich honeydew secretions [1]. However, the most serious damage caused by *B. tabaci* is virus transmission. *B. tabaci* is a vector for >100 different plant viruses, primarily Old and New World begomoviruses of the family Geminiviridae. The whitefly's vectoring abilities are not limited to begomoviruses, and new viruses belonging to Potyviridae, Closteroviridae, Luteoviridae, and Betaflexiviridae were also recently reported to be vectored by *B. tabaci* [2–4].

*B. tabaci* is a complex of morphologically indistinguishable species. Based on sequence polymorphism in defined mitochondrial genes, it is now agreed that *B. tabaci* comprises 11 species groups, each including some species-complex members, previously termed as biotypes [5, 6]. The 2 most polyphagous and invasive species in this complex are MEAM1 and MED [7]. Surveys conducted over the years in Israel have reported the presence of those 2 species only [8].

Recently, the genomes of both MEAM1 and MED have been sequenced and published [9, 10], creating a wealth of new resources for genetic and molecular studies. *B. tabaci* genomes, which are still being annotated, are highly divergent from those of previously sequenced hemipteran species and show vast expansions in gene families related to metabolism and insecticide resistance [9].

Mass spectrometry (MS)–based proteomic approaches have become a prevalent tool in research of various biological systems—from humans to arthropods. Recent studies performed on arthropods and entomopathogenic viruses were able to isolate and identify viral structural proteins and virions from both insect cell cultures and hemolymph [11, 12]. Proteomic studies comparing efficient and non-efficient virus vector clone lines in aphids were able to identify protein markers linked to transmission ability: in the greenbug aphid, *Schizaphis graminum*, and *Cereal yellow dwarf virus-*RPV (CYDV-RPV) [13], and in the English grain aphid, *Sitobion avenae* and *Barley yellow dwarf virus-*PAV (BYDV-PAV) [14]. Proteomic studies conducted with *B. tabaci* thus far have focused on targeting proteins or genes for the development of new insecticides [15] or for studying insecticide resistance mechanisms [16].

In this study we performed a discovery MS analysis using 9 populations from the MEAM1 and MED species collected in Israel and Croatia that vary in their *Tomato yellow leaf curl virus* (TYLCV) transmission ability. We compared the proteomic profiles between efficient TYLCV vector populations within each species and between the 2 species. We were able to identify previously undescribed proteins from *B. tabaci*, some of which are important for virus transmission. Such candidate proteins shed more light on the molecular mechanisms that underlay the insect-virus interactions during TYLCV transmission by *B. tabaci*.

## Analyses

### TYLCV transmission assays

**Figure 1:**
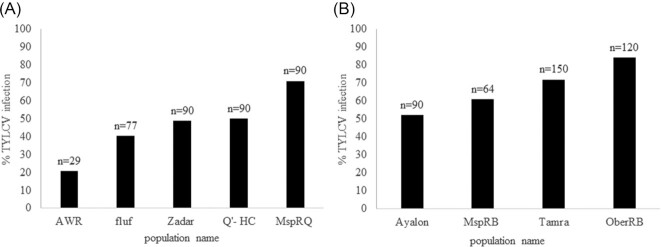
TYLCV Transmission abilities of MED (A) and MEAM1 (B) species populations used in this study. MspRQ and OberRB are the populations with the highest transmission efficiency in each species. Numbers above columns represent the number of plants tested for virus transmission with whiteflies from each population.

To characterize our selected populations with regard to their TYLCV transmission abilities we performed several transmission experiments. We identified a gradient of transmission abilities, with MEAM1 being in general a better vector for the virus compared to MED populations (Fig. 1A and B). Our results are consistent with previously published results from Israel [17]. We identified MspRQ as the most efficient vector population of the MED species (Fig. 1A) and OberRB as the most efficient MEAM1 species TYLCV vector (Fig. 1B).

### Proteomic analysis

We used shotgun proteomics to compare the abundance of protein profiles of the 9 different populations of the 2 different *B. tabaci* species collected in Israel and Croatia (Fig. 1). Data for each population were composed of 3 biological replicates and 3 technical replicates per biological one. A principal component analysis (PCA) made of all data showed a low percentage of variance originating from the biological replicates, proving high reproducibility of the technical and biological replicates (Supplementary Fig. S1).

We were able to identify on average 3,350 proteins from 2,510 protein families, with a mean false discovery rate (FDR) of 0.9% in each replicate. We then compared the quantity of all peptides and proteins in order to identify proteins that differ in their abundance between TYLCV efficient vector populations compared to the other populations of the respective species. We found that the general level of variability was much higher between the different MED populations than between the MEAM1 populations. We limited our analysis to up to 15,000 peptides showing >2-fold change in abundance. In the MEAM1 population peptides, we used only peptides with *P* < 0.05;  this approach produced too many results in MED, so we reduced our analysis to peptides with *P* < 0.01. This coincides with the findings showing that while MEAM1 and MED are derived from the same ancestral species, during speciation, MEAM1 remained stable while MED continued to separate into more species such as MED, J, L, and others [5]. Therefore, while MEAM1 populations are more unified in their proteomic profiles, MED populations show higher variance.

### Proteins differentially abundant in MEAM1 biotype efficient vector population

**Figure 2:**
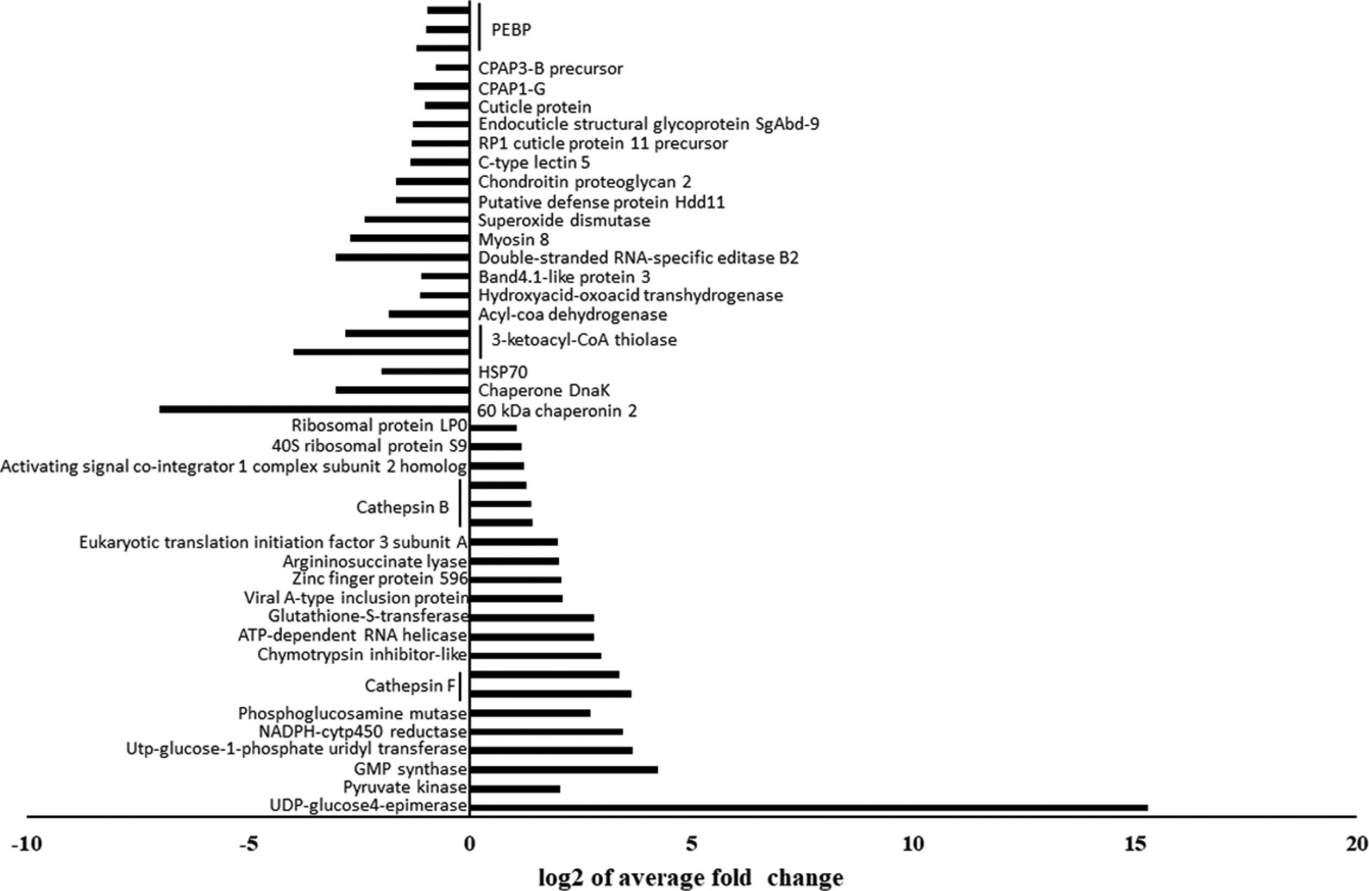
Top 40 differentially abundant proteins in OberRB. The 20 proteins with significantly low abundance and the 20 proteins with significantly high abundance in the MEAM1 efficient vector population compared to all other MEAM1 populations.

We compared each efficient vector population to other populations of the same species and identified several interesting candidate proteins with possible functional roles in virus transmission (Fig. 2). Of 108 proteins that are significantly more abundant in the efficient biotype MEAM1 vector, the proteins with >1 peptide identified and with the highest abundance were as follows: a eukaryotic translation initiation factor 3, Cathepsins B and F, and a viral A inclusion protein (full list in Supplementary Table S1). Cathepsins are a large family of proteases; in arthropods they are primarily expressed in the digestive system. It is postulated that Cathepsin B proteases are excreted into the plant phloem or that they may assist in resistance to plant defensive secondary metabolites found in the plant sap [18].

We found 85 proteins with significantly lower abundance in the MEAM1 efficient vector compared to all other MEAM1 populations (selected proteins shown in Fig. 2, full list in Supplementary Table S1). Of these proteins we found chondroitin proteoglycan, HSP70, Hdd11 defense protein, and 2 cuticular proteins analogous to peritrophin (CPAP), all of which were previously studied in relation to virus transmission or immune responses. All but chondroitin proteoglycan are known as virus transmission inhibitors; Hdd11 and CPAP are related to the immune system and HSP70 was previously shown in whiteflies to inhibit TYLCV passage through the insect midgut epithelial cells [19–21].

### Proteins differentially expressed in MED biotype efficient vector population

**Figure 3:**
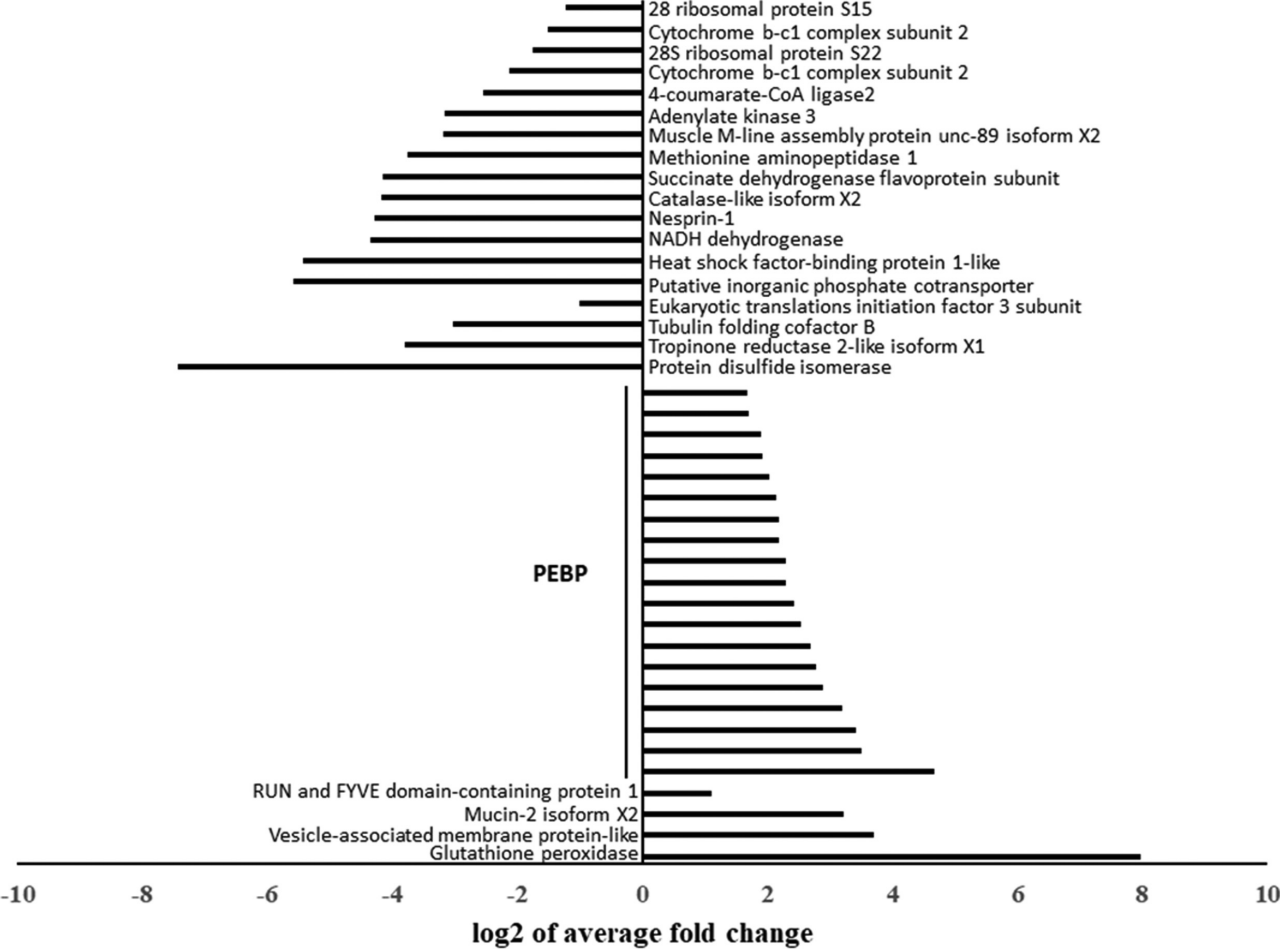
Top 40 differentially abundant proteins in MspRQ. The 20 proteins with significantly low abundance and the 20 proteins with significantly high abundance in the MED efficient vector population compared to the other MED populations.

Among the 41 proteins significantly more abundant in the MED efficient vector compared with all other MED populations, 20 were identified as phosphatidylethanolamine binding proteins (PEBPs) (Fig. 3, full list in Supplementary Table S1). Alignment of the DNA and amino acid sequences of those 20 candidates showed low sequence identity, implying that these peptides belong to different proteins of the same protein family.

Other prominent highly abundant proteins include a protein with a RUN and FYVE domain, a vesicle-associated membrane protein, glutathione peroxidase, and mucin-2 like protein. The FYVE domain functions in membrane trafficking [22]. A FYVE-containing phosphatidylinositol-3-phosphate in mammals was found to be a binding site initiating endocytosis and cell invasion of *Vesicular stomatitis virus*. Inhibition of the FYVE domain of the protein inhibited infection [23]. In arthropods, a FYVE domain containing a zinc finger was found upregulated in *Litopenaeus vannamei* shrimp resistant to *Taura syndrome virus* [24].

A mucin-like protein was associated with the passage of *Plasmodium* through the guts of the mosquito *Aedes aegypti* [25]. It is also a possible target protein of Baculoviruses while crossing the plasma membrane of the arthropod host [26].

Proteins with lower abundance in the efficient TYLCV vector population included mitochondrial ribosomal and cytochrome b proteins, metabolism-related enzymes such as methionine aminopeptidase 1 and adenylate kinase 3, a heat shock factor binding protein, tubulin folding protein, and more (Fig. 3, full list in Supplementary Table S1). Cytochrome b was found to be downregulated in *Anopheles gambiae* midguts after acquisition of O'nyong-nyong virus [27].

### Bacterial proteins differentially expressed in TYLCV efficient vector populations

**Figure 4:**
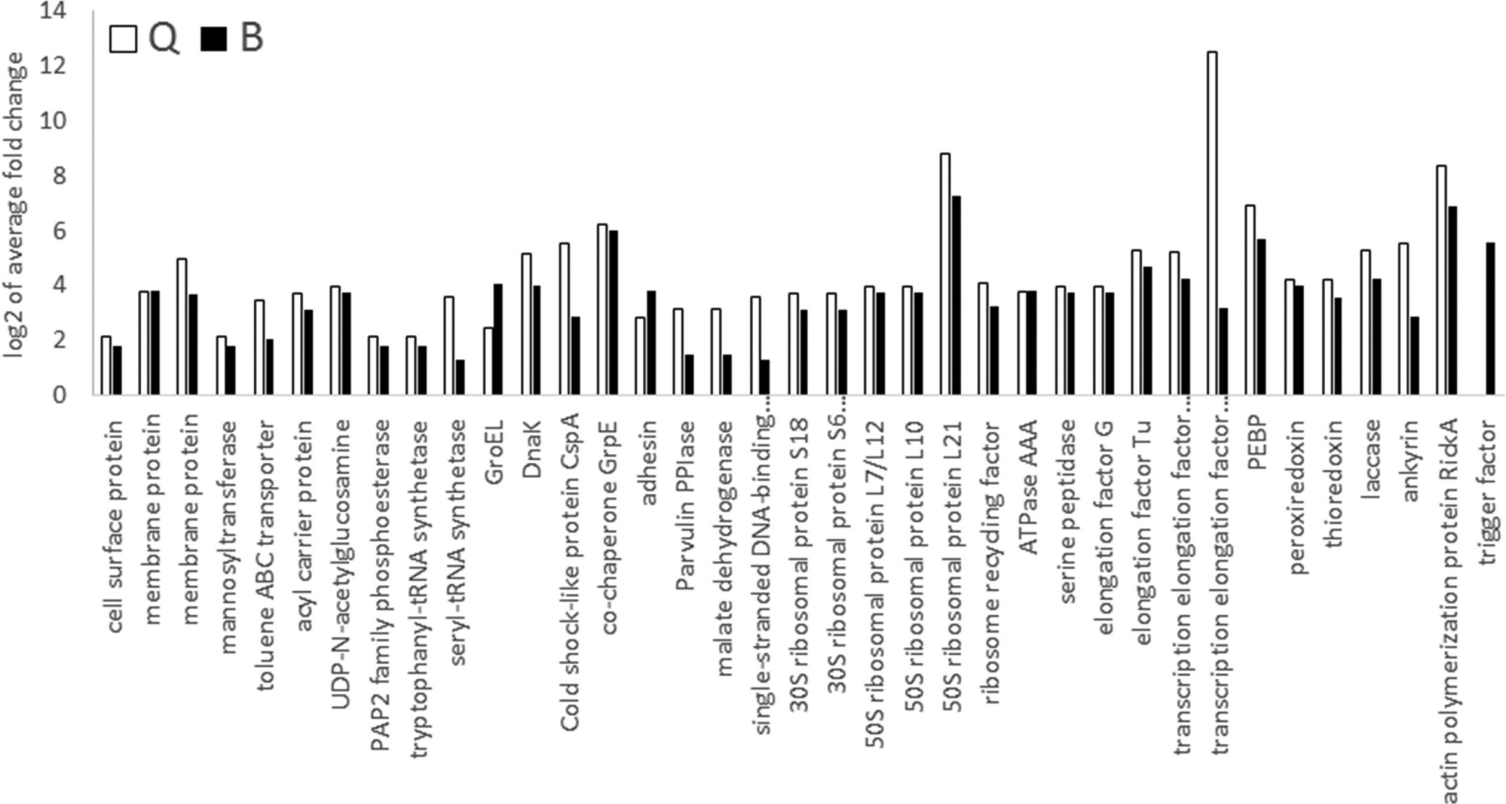
*Rickettsia* proteins found at high quantities in both MEAM1 and MED efficient vector populations. Common bacterial proteins significantly abundant in MEAM1 (black) and MED (white) efficient vectors.

Among the 41 common proteins highly abundant in the efficient vector populations from both species, 37 were bacterial proteins, all from the facultative endosymbiont *Rickettsia. Rickettsia* has been previously implicated in virus transmission. Although each species has a different secondary endosymbiont bacterial composition, *Rickettsia* proteins were the only ones found to have significantly different abundance in the efficient vector populations. In the MEAM1 efficient vector population a total of 53 proteins were significantly upregulated, 37 of which common with the MED efficient vector population. In the MED population only 1 *Rickettsia* protein was not shared with MEAM1. The abundant *Rickettsia* proteins are adhesin and other membrane proteins and transporters, GroEL and chaperonins, transcription and elongation factors, ribosomal proteins, actin polymerization protein, and trigger factor proteins. Fold change of those identified proteins was higher in MED for all proteins but GroEL and adhesin proteins, whose fold change was higher in MEAM1 populations (Fig. 4, full list in Supplementary Table S1).

**Figure 5:**
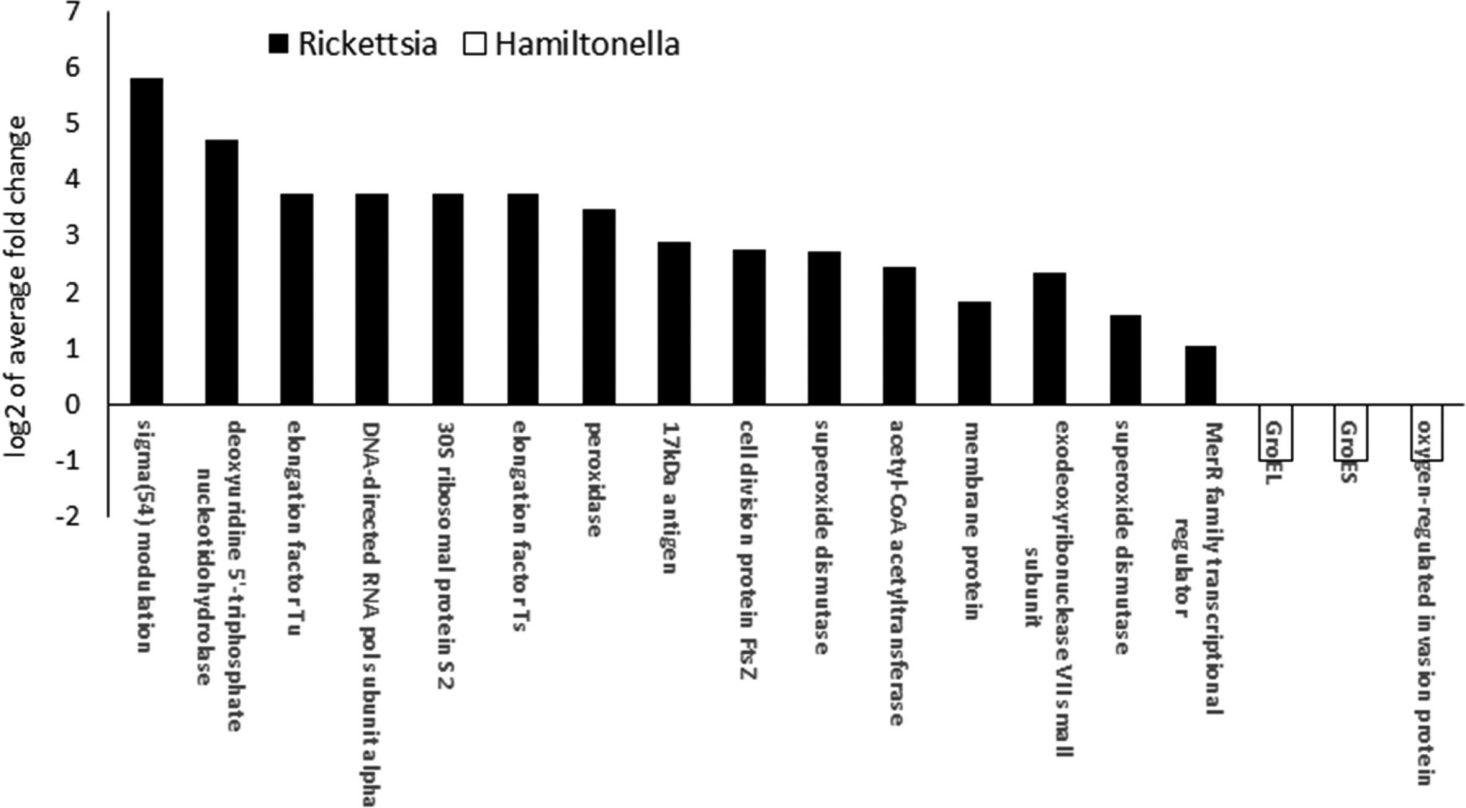
Additional symbiont (*Rickettsia* and *Hamiltonella)* proteins with high and low abundance in the MEAM1 efficient vector population.

In the MEAM1 efficient vector population, an additional 15 *Rickettsia* proteins were significantly more abundant compared to the rest of the MEAM1 populations (Fig. 5, full list in Supplementary Table S1). Six of them are transcription or DNA editing related, 2 are membrane-related proteins, 2 ribosomal proteins, and the rest are uncategorized. One of these proteins is ftsZ, which plays a crucial role in the development of the central cytoskeletal septum during cell division, strengthening our hypothesis that *Rickettsia* is dividing and proliferating more in this efficient vector population [28]. Three *Hamiltonella* proteins were downregulated in the MEAM1 efficient vector compared to the other populations (1.98-fold change). One of them is the *Hamiltonella* GroEL protein, previously mentioned in this section. It is surprising that this protein, previously found to improve TYLCV transmission, has a lower abundance in the efficient TYLCV vector. It was hypothesized that the *Hamiltonella* GroEL aids TYLCV virions to avoid the insect immune system in the whitefly hemolymph [17]. Our results, indicating proliferation of *Rickettsia*, could imply that the immune system of efficient vector populations is a “lenient” one and that therefore TYLCV virions need not bind to *Hamiltonella* GroEL to survive the passage through the hemolymph.

## Discussion

Discovery of protein abundance in both efficient TYLCV vectors compared to the rest of the tested populations resulted in 6 proteins that have significantly different abundances in both efficient vector populations. However, all proteins with significantly different abundances that were common to both efficient vector populations of the 2 species showed different trends: catalase, a PEBP, and cyclophilin were highly abundant in the MED efficient vector while Vitellogenin and an antimicrobial protein, Alo-2, were highly abundant in MEAM1. Several of these proteins were previously reported with regard to virus transmission; cyclophilin, a peptydil prolyl-isomerase, was shown to be linked to CYDV-RPV transmission by the aphid *Schizaphis graminum*. Cyclophilin was found to be upregulated in efficient vector clone lines compared to inefficient vector lines. It was also shown to bind to CYDV-RPV virions [29]. Different isoforms of the protein were shown to segregate between clones with different CYDV-RPV transmission efficiencies [30]. Three cyclophilin genes were identified in *B. tabaci* MEAM1 species: B, D, and G. The expression of Cyclophilin B was shown to be induced upon TYLCV infection, in the whitefly midgut [31]. TYLCV CP and cyclophilin were shown to co-localize in *B. tabaci* midguts and ovaries. Finally, feeding whiteflies with anti-cyclophilin antibodies, a cyclophilin inhibitor, or cyclophilin double-stranded RNA greatly reduced TYLCV transmission rates [31, 32].

Alo-2 is a protein of the Knottin family, a highly diverse protein family with 1 common domain, the knottin fold. Knottin proteins have been extensively studied in arthropods such as *Drosophila* and various Coleopterans with regards to the systemic immune response. Members of the Knottin family have been described to have antifungal and antibacterial functions [33, 34], while no antiviral response has yet been identified. Alo-2 is likely to function in the immune response of *B. tabaci* and therefore its upregulation in the efficient vector is unexpected.

Vitellogenin is a large phospholipoglycoprotein involved in oogenesis and presumed to be a storage nutrient in the yolk. It is hypothesized to function as a hemagglutinating factor and an antibacterial effector in organisms from multiple kingdoms [35]. Wei et al. [36] demonstrated the crucial role of Vitellogenin in transovarial transmission of TYLCV in *B. tabaci* MEAM1 species, thus putting an end to a long-standing debate on the subject [36–39]. Wei et al. have shown that Vitellogenin binds to TYLCV coat protein and aids in the virus translocation into developing eggs inside the ovaries. Interestingly, this study showed that TYLCV was transovarially transmitted to eggs in mature females (11 days after emergence) significantly more efficiently than in young females (1 day after emergence). All samples collected for our study were 1–5 days after emergence, a life stage indicated to have lower TYLCV transovarial transmission efficiency; however we found elevated quantities of Vitellogenin in both MEAM1 and MED efficient vector populations. Interestingly, peptides spanning the entire Vitellogenin sequence were found in high abundances in the MEAM1 efficient vector. In MED, peptides from a certain region of the protein were found to have low abundances, unlike the rest of the protein (Supplementary Fig. S2). This might hint at the existence of different isoforms of Vitellogenin in the 2 species.

Half of the significantly abundant proteins in the MED efficient vector population were identified as PEBPs (Fig. 3). PEBPs were found to be linked to immune response activation against bacterial infection via the Toll immune pathway in *Drosophila melanogaster* [40, 41].

A PEBP was also found to be necessary for HIV1 infection [42]. The recent sequencing of the MEAM1 biotype genome showed that PEBP genes are >10-fold more abundant in the *B. tabaci* MEAM1 genome compared to 15 other arthropod genomes [9]. This finding, along with our data, hints at the important role this gene family plays in whiteflies, where they are likely participating in various processes, including virus transmission.

The significantly different abundances of all these proteins in the MED efficient vector population suggest that its midgut is more permeable and thus TYLCV circulation is more efficient. The gut barrier is known to be the first and often most important barrier for an insect-transmitted pathogen to cross along the transmission pathway especially in the whitefly-begomovirus interaction [43], and this barrier determines the efficiency and specificity of transmission.

In the MEAM1 efficient vector population, we identified 108 proteins with significantly higher abundance, among which Cathepsins B and F were highly represented. A total of 78 proteins were of significantly lower abundance in the MEAM1 efficient vector population that in the other MEAM1 populations. We identified several of them as immune system proteins and known virus transmission inhibitors such as Chondroitin proteoglycan, HSP70, Hdd11 defense protein, and 2 CPAPs. It is therefore expected that those proteins were downregulated in the efficient vector, resulting in observed lower abundances, and demonstrating a “less stringent” immune system in which TYLCV virions have higher chances of making a full passage through the whitefly tissues and ensuring successful transmission.

Among the 41 common proteins highly abundant in the efficient vector populations from both species, 37 were bacterial proteins encoded by *Rickettsia. Rickettsia* is the only shared secondary endosymbiont between MED and MEAM1 in Israel [8]. Of the 9 populations tested in this experiment, 6 were infected with *Rickettsia* (see Table 1); however, no correlation was found between the presence of *Rickettsia* and TYLCV transmission efficiency. This could indicate that infection only is not enough to improve transmission ability; additional genes from the bacterium need to be expressed. Our data also do not suggest that there are higher titers of the bacterium in either of the efficient vector populations.

**Table 1:** Populations collected and used in this study

Population name	Symbiont population composition						Collection site
	*Portiera*	*Hamiltonella*	*Arsenophonus*	*Wolbachia*	*Rickettsia*	*Cardinium*	
**MED populations**							
Q-AWR	+		+	+	+		Ayalon Valley, Israel
fluf	+		+	+	+		Israel
Zadar	+	+	+	+			Zadar, Croatia
Q'-HC	+	+				+	Croatia
MspRQ	+		+	+	+		Israel
**MEAM1 populations**							
Ayalon	+	+			+		Ayalon Valley, Israel
MspRB	+	+					Israel
Tamra	+	+			+		Tamra, Israel
OberRB	+	+			+		Israel

Highly abundant *Rickettsia* proteins include adhesin and other membrane proteins and transporters, GroEL and chaperonins, transcription and elongation factors, ribosomal proteins, actin polymerization protein, and trigger factor proteins. High quantities of proteins from all these groups indicate that the bacteria are propagating and undergoing cell division characteristic of a “log phase” of bacterial growth in the better vector populations.

The present study shows elevated levels of Vitellogenin in efficient vector populations. We previously demonstrated that high levels of Vitellogenin and high fecundity are associated with the presence of *Rickettsia* [44]. Taken together, these results point to another possible effect of this bacterium on TYLCV transmission. The role of bacterial endosymbionts in plant virus transmission is still under debate [45]. We have previously demonstrated the significant effect of the secondary endosymbionts *Rickettsia* and *Hamiltonella* from *B. tabaci* on TYLCV transmission by this insect [17, 46, 47].

## Conclusions

In this study we have produced an extensive proteomic database for *B. tabaci*, a non-model insect, which could be useful for studies related to understanding the biology and ecology of this important insect pest and virus vector. We further demonstrated the possible uses of this database by comparing the proteomic profiles of different vector populations from 2 species and correlated the results with their TYLCV transmission efficiencies.

Our results demonstrate that different molecular pathways in the insect may participate in the transmission of plant viruses, and some might be crucial for the passage of the virus through insect organs. While in MEAM1 species we observed a decline of immune-related genes and virus transmission inhibitors, in MED we observed a wealth of possible target proteins that aid in TYLCV movement within and between cells. Most interestingly, we find that PEBPs, a recently described and highly expanded protein family in whiteflies, have a strong link to TYLCV transmission in MED. The only shared group of proteins between both efficient vector populations of both species and highly abundant in both are proteins encoded by the endosymbiont *Rickettsia*. Utilizing the database we developed in this study we uncovered a high number of proteins that play a role in the transmission of TYLCV, and possibly other Begomoviruses. This is an important step for functional studies in this insect related to its biology and to virus transmission.

## Methods

### Insect collection and rearing in the laboratory

*B. tabaci* populations were collected from various locations in Israel and Croatia (Table 1) and reared on cotton seedlings (*Gossypium hirsutum* L. cv. Acala) in insect-proof cages maintained in growth rooms under standard conditions of 25°C ± 2°C, 60% relative humidity, and a 14-h light/10-h dark photoperiod. Then 3–5 biological replicates containing 200–500 individuals were collected from each population up to a week after adult emergence. Samples were stored at −80°C till samples from all populations were collected.

### Virus transmission assays

To calculate TYLCV transmission efficiencies of whitefly populations, 6–7-day-old adults from each population were given a 48-h acquisition access period on a TYLCV-infected tomato plant. The insects were then used for a 7-day inoculation access period on 4-week-old, non-infected tomato plants, 1 whitefly per plant, in leaf clip cages. Two weeks after inoculation, young leaves were collected from the plants for DNA extraction (using the Dellaporta protocol, detailed in [46]) and PCR for TYLCV detection (using primers listed in [46]). Three replicates of 30 plants each were performed for each population (except the Q-AWR population that was terminated after the first assay owing to technical problems).

### Protein extractions and preparations for MS analysis

Proteins were extracted as described in [48]; samples were ground using a mortar and pestle while kept frozen using liquid nitrogen. A quantity of 1 mL of 10% TCA-acetone, 2% β-mercaptoethanol was added per sample. Samples were then incubated for 16 h at −20°C, then centrifuged at 5,000*g*, 4°C, 30 minutes. Pellets were saved and washed 3 times with cold acetone, dried, and resuspended in 8 M urea in 100 mM ammonium bicarbonate (ABC).

Protein was quantified using a Bradford assay; protein integrity was examined by running 5 µg from each sample on 1D gel with bovine serum albumin as a control, and a Coomassie Brilliant Blue staining.

Three biological replicates were chosen per population. Protein samples then proceeded to reduction, cystein blocking, and trypsin digestion; 50 µg of protein was added to a final volume of 10 mM of dithiothreitol in 100 mM ABC; samples were then incubated at 30°C for 1 h. A final volume of 30 mM of methyl methanethiosulfonate (MMTS) in 100 mM ABC was added and samples were incubated for 1 h at room temperature.

Samples were then diluted to ∼1 M urea with 100 mM ABC and trypsin was added in a 1:50 ratio (trypsin: protein). Samples were incubated for 16 h at 37°C, desalted using Waters Sep Pak SPE cartridges (according to manufacturer's protocol), dried, and kept at −80°C till MS analyses.

### MS runs

The dried tryptic digests were solubilized in 50 µL 0.2% trifluoracetic acid and 2% acetonitrile by vortexing for 10 minutes at 37°C and bath sonication for 5 minutes. The solubilized digests were centrifuged at 10,000g for 5 minutes to pellet any particulates that might cause high-performance liquid chromatography clogging, and the supernatants were carefully removed and placed into autosampler vials. Injections of 3 µL resulted in ∼2 µg total peptide loaded onto the column. The sample order was randomized and blocked by biological replicates. Every third injection was a random.

All MS was performed on an LTQ-Orbitrap-Velos (Thermo Fisher Scientific, Washington, DC, USA). Samples were loaded onto a 150-μm Kasil fritted trap packed with Jupiter C12 90 Å material (Phenomenex, Torrance, CA, USA) to a bed length of 2 cm at a flow rate of 2 µL/min. After loading and desalting using a total volume of 10 µL of 0.1% formic acid plus 2% acetonitrile, the trap was brought online with a pulled fused-silica capillary tip (75-μm i.d.) packed with 40 cm of Reprosil-Pur C18-AQ (3-μm bead diameter, Dr. Maisch) mounted in an in-house–constructed microspray source and placed in line with a Waters Nanoacquity binary UPLC pump plus autosampler. Peptides were eluted off the column using a gradient of 2–35% acetonitrile in 0.1% formic acid over 120 minutes, followed by 35–60% acetonitrile over 10 minutes at a flow rate of 250 nL/min.

The mass spectrometer was operated using data-dependent acquisition mode, where a maximum of 15 MS/MS spectra were acquired per MS spectrum. The resolution for MS was 60,000 at *m*/*z* 400 covering the *m*/*z* range of 400–2,000. MS/MS spectra were acquired using a linear ion trap that provided unit resolution. The automatic gain control target for MS in the orbitrap was 1e6, whereas for MS/MS it was 8,000, and the maximum fill times were 20 and 80 msec, respectively. The MS/MS spectra were acquired using an isolation width of 2 *m*/*z* and a normalized collision energy of 35. The precursor ion threshold intensity was set to 5,000 to trigger an MS/MS acquisition. Furthermore, MS/MS acquisitions were prevented for precursor charge states of 1, or if the charge state could not be discerned from the MS spectrum. Dynamic exclusion (including all isotope peaks) was set for 20 seconds.

Total ion current and base peak chromatograms were analysed to ensure that even amounts of protein extractions were injected from all samples and to study the reproducibility and the spread across the gradient of the technical and biological replicates (see Supplementary Figs S3 and S4). MS data were deposited to the ProteomeXchange consortium via PRIDE [49] with identifier PXD016964.

### MS analysis and data annotations

An initial search of all animal protein sequences on NCBI (monthly) showed ∼1,000 proteins identified per run. A FASTA database of whitefly and whitefly endosymbiont bacterial DNA sequences from NCBI was compiled and used for Mascot searching. Using this as a database, the search was drastically improved, with an average of 3,350.5 peptides being matched per LCMS run, with an average FDR of 0.9%. Percolator [50, 51] was used to correct for multiple hypothesis testing and computing q-values. Mascot files were then loaded into the Progenesis QI program (Nonlinear, Milford,MA,USA) and aligned to a randomly selected reference run. Each and every run was then aligned and problematic regions with low alignment were removed. The data were then analysed 3 times, once for every technical replicate of every biological replicate. Hence the data were analysed as 3 separate experiments, each containing 3 biological replicates for every population and 1 technical replicate of each. Average normalized abundance was calculated for each protein, based on protein features without conflict only, and fold change between the highest and lowest values was calculated. All peaks were then compared and only those showing a >2-fold change in abundance with *P* < 0.05 in MEAM1 and *P* < 0.01 in MED were selected for analysis. Further sifting of the data was done to keep only proteins that had ≥1 unique peptide sequence identified. All 3 final lists of proteins, from the 3 technical replicates, were then compared and only proteins that appeared in ≥2 of the 3 were kept (see Supplementary Table S1).

## Availability of Supporting Data and Materials

MS and raw data reported in this article were deposited to the ProteomeXchange consortium via PRIDE [49] with identifier PXD016964. Other data further supporting this work are openly available in the *GigaScience* repository, GigaDB [52].

## Additional Files

**Table 1:**All Differentialy Abundant Proteins (DAPs). All DAPs in each species and common. Bemisia tabaci and bacterial proteins are listed with the number of peptides identified for each, uniqe and otherwise, as well as score, fold change and NCBI identifier for nucleic acid sequence.

**Figure S1:** Peptide PCAs for selected populations. Two PCA analyses for 3 randomly selected populations (of 9 in the experiment—3 in A and 3 in B). Data for each PCA consisted of quantification of all peptides found in all 3 biological replicates performed for each population and all 3 technical replicates performed per biological replicate

**Figure S2:**  *B. tabaci* complete Vitellogenin amino acids sequence. Highlighted are peptides identified to be of high abundance in the MEAM1 efficient TYLCV vector compared to the rest of the MEAM1 populations. Formatted are the peptides identified to be of low abundance in the MED efficient vector compared to the rest of MED populations.

**Figure S3:** Total ion current (TIC) of 3 selected runs. The TIC is the summed intensity of all ions (all *m*/*z*s) for the entire LCMS run. A and B are duplicate injections of the same sample and C is a biological replicate injection. TIC shows high reproducibility and a good spread across the gradient. The intensity of the second biological replicate (C) seems lower than the first biological replicate (A and B), which could be due to slightly lower concentration. Because the data were normalized for comparison, this is not a problem.

**Figure S4:** Base peak chromatograms of 3 selected runs. Base peak chromatograms for the same 3 runs as in Fig. S3. A and B are duplicate injections of the same sample and C is a biological replicate injection. The base peak chromatogram is the intensity of the most intense *m*/*z* peak during each scan. The base peaks are very reproducible between analytical replicates (A and B) and between biological replicates (A, B compared to C). The retention times are reproducible, many abundant peaks to within 1 minute.

## Abbreviations

ABC: ammonium bicarbonate; CPAP: cuticular proteins analogous to peritrophin; CYDV:*Cereal yellow dwarf virus*; FDR: false discovery rate; HSP70: heat shock protein 70; LCMS: liquid chromatography mass spectrometry; MEAM1: Middle East Asia Minor 1; MED: Mediterranean; MS: mass spectrometry; MS/MS: tandem mass spectroscopy; NCBI: National Center for Biotechnology Information; PCA: principal component analysis; PEBP: phosphatidylethanolamine binding protein; TYLCV: *Tomato yellow leaf curl virus*; TIC: total ion current.

## Competing interests

The authors declare that they have no competing interests.

## Funding

This work was funded by a student travel grant from the United States–Israel Binational Agricultural Research and Development Fund, FundRef http://dx.doi.org/10.13039/100006031, award No. GS-27–14 to Adi Kliot.

## Authors' Contributions

A.K.: Investigation, formal analysis, validation, visualization, writing: original draft, funding acquisition.

M.J.M.: Formal analysis.

R.S.J.: Formal analysis.

G.L.: Resources.

S.K.: Resources.

H.C.: Supervision, writing: review & editing.

M.H.: Methodology, resources, validation, data curation, funding acquisition, writing: review & editing.

M.G.: Funding acquisition, conceptualization, supervision, writing—review & editing.

## Supplementary Material

giaa124_GIGA-D-20-00096_Original_Submission

giaa124_GIGA-D-20-00096_Revision_1

giaa124_GIGA-D-20-00096_Revision_2

giaa124_GIGA-D-20-00096_Revision_3

giaa124_GIGA-D-20-00096_Revision_4

giaa124_Response_to_Reviewer_Comments_Original_Submission

giaa124_Response_to_Reviewer_Comments_Revision_1

giaa124_Response_to_Reviewer_Comments_Revision_2

giaa124_Response_to_Reviewer_Comments_Revision_3

giaa124_Reviewer_1_Report_Original_Submission Pedro Filho Noronha de Souza -- 5/4/2020 Reviewed

giaa124_Reviewer_2_Report_Original_Submission Prem Prakash Das -- 5/7/2020 Reviewed

giaa124_Reviewer_2_Report_Revision_1 Prem Prakash Das -- 8/3/2020 Reviewed

giaa124_Supplemental_Files
