# Supplementary material for: A proteomic approach reveals possible molecular mechanisms and roles for endosymbiotic bacteria in begomovirus transmission by whiteflies
Source: Gigascience. 2020 Nov 13;9(11):giaa124. doi: 10.1093/gigascience/giaa124 (PMC7662926; doi:10.1093/gigascience/giaa124)
Supplement: giaa124_GIGA-D-20-00096_Original_Submission [file giaa124_giga-d-20-00096_original_submission.pdf]

GigaScience

A proteomic approach reveals possible molecular mechanisms and roles for endosymbiotic bacteria in begomovirus transmission by whiteflies

--Manuscript Draft--

**Manuscript Number:**	GIGA-D-20-00096
**Full Title:**	A proteomic approach reveals possible molecular mechanisms and roles for endosymbiotic bacteria in begomovirus transmission by whiteflies
**Article Type:**	Data Note
**Funding Information:**	
**Abstract:**	Background Many plant viruses are vector-borne and depend on arthropods for transmission between host plants. Begomoviruses, the largest, most damaging and emerging group of plant viruses, infect hundreds of plant species and new virus species of the group are discovered each year. Begomoviruses are transmitted by members of the whitefly Bemisia tabaci species complex in a persistent-circulative manner. Tomato yellow leaf curl virus (TYLCV) is one of the most devastating begomoviruses worldwide and causes major losses in tomato crops as well as in many agriculturally important plant species. Different B. tabaci populations vary in their virus transmission abilities; however, the causes for these different abilitites are attributed among others to genetic differences among vector populations, as well as to differences in the bacterial symbiont flora of the insects.<br>Results Here, we performed discovery proteomic analyses in nine whiteflies populations from both Middle East Asia Minor I (MEAM1 formerly known as B biotype) and Mediterranean (MED formerly known as Q biotype) species with different TYLCV transmission abilities. The results provide the first comprehensive list of candidate insect and bacterial symbiont (mainly Rickettsia) proteins associated with virus transmission. A protein database, over-expressed and down downregulated proteins from efficient vector populations from two different B. tabaci species are provided.<br>Conclusions Our data demonstrate that the proteomic signature of better vectors populations, differ considerably when compared to the rest of less good vector populations from the two whitefly species tested in this study. While MEAM1 efficient vector populations has a more lenient immune system, the Q efficient vector populations expressed more proteins possibly implicated in virus passage through cells. Both species show a strong link of the facultative symbiont Rickettsia to virus transmission efficiency.
**Corresponding Author:**	Murad Ghanim<br>Agricultural Research Organization Volcani Center<br>Rishon LeZion, ISRAEL
**Corresponding Author Secondary Information:**	
**Corresponding Author's Institution:**	Agricultural Research Organization Volcani Center
**Corresponding Author's Secondary Institution:**	
**First Author:**	Adi Kliot
**First Author Secondary Information:**	
**Order of Authors:**	Adi Kliot
	Richard Johnson
	Michael MacCoss
	Svetlana Kontsedalov
	Galina Lebedev
	Henryk Czosnek
	Michelle Heck
	Murad Ghanim

Powered by Editorial Manager® and ProduXion Manager® from Aries Systems Corporation

Order of Authors Secondary Information:	
**Additional Information:**	
**Question**	**Response**
Are you submitting this manuscript to a special series or article collection?	No
**Experimental design and statistics**<br><br>Full details of the experimental design and statistical methods used should be given in the Methods section, as detailed in our Minimum Standards Reporting Checklist. Information essential to interpreting the data presented should be made available in the figure legends.<br><br>Have you included all the information requested in your manuscript?	Yes
**Resources**<br><br>A description of all resources used, including antibodies, cell lines, animals and software tools, with enough information to allow them to be uniquely identified, should be included in the Methods section. Authors are strongly encouraged to cite Research Resource Identifiers (RRIDs) for antibodies, model organisms and tools, where possible.<br><br>Have you included the information requested as detailed in our Minimum Standards Reporting Checklist?	Yes
**Availability of data and materials**<br><br>All datasets and code on which the conclusions of the paper rely must be either included in your submission or deposited in publicly available repositories (where available and ethically appropriate), referencing such data using a unique identifier in the references and in the "Availability of Data and Materials" section of your manuscript.<br><br>Have you have met the above requirement as detailed in our Minimum Standards Reporting Checklist?	Yes

Powered by Editorial Manager® and ProduXion Manager® from Aries Systems Corporation

Manuscript
Click here to access/download;Manuscript;TYLCV proteomics submit version MG.docx

A proteomic approach reveals possible molecular mechanisms and roles for endosymbiotic bacteria in begomovirus transmission by whiteflies

Adi Kliot,[a,b,c] Richard S Johnson,[d] Michael J MacCoss,[d] Svetlana Kontsedalov,[a] Galina Lebedev,[a] Henryk Czosnek,[b] Michelle Heck,[d] Murad Ghanim,[a*]

[a] Department of Entomology, The Volcani Center, Rishon LeZion, Israel

[b] Institute of Plant Sciences and Genetics in Agriculture, Robert H. Smith Faculty of Agriculture, Food and Environment, Hebrew University of Jerusalem, Rehovot, Israel

[c] Earlham Institute, Norwich, UK

[d] University of Washington

[e] USDA-Agricultural Research Service, Boyce Thompson Institute for Plant Research, Department of Plant Pathology and Plant-Microbe Biology, Cornell University, Ithaca, New York, USA

* Corresponding author

Email list:
Adi Kliot: adiaaa@gmail.com
Michael MacCoss: maccoss@uw.edu
Richard Johnson: rj8@uw.edu
Svetlana Kontsedalov: nasvetla@yahoo.com
Galina Lebedev: galinal@volcani.agri.gov.il
Henryk Czosnek: hanokh.czosnek@mail.huji.ac.il
Murad Ghanim: ghanim@volcani.agri.gov.il
Michelle Heck: mlc68@cornell.edu

Abstract

Background Many plant viruses are vector-borne and depend on arthropods for transmission between host plants. Begomoviruses, the largest, most damaging and emerging group of plant viruses, infect hundreds of plant species and new virus species of the group are discovered each year. Begomoviruses are transmitted by members of the whitefly *Bemisia tabaci* species complex in a persistent-circulative manner. *Tomato yellow leaf curl virus* (TYLCV) is one of the most devastating begomoviruses worldwide and causes major losses in tomato crops as well as in many agriculturally important plant species. Different *B. tabaci* populations vary in their virus transmission abilities; however, the causes for these different abilitites are attributed among others to genetic differences among vector populations, as well as to differences in the bacterial symbiont flora of the insects.

Results Here, we performed discovery proteomic analyses in nine whiteflies populations from both Middle East Asia Minor I (MEAM1 formerly known as B biotype) and Mediterranean (MED formerly known as Q biotype) species with different TYLCV transmission abilities. The results provide the first comprehensive list of candidate insect and bacterial symbiont (mainly *Rickettsia)* proteins associated with virus transmission. A protein database, over-expressed and down downregulated proteins from efficient vector populations from two different *B. tabaci* species are provided.

Conclusions Our data demonstrate that the proteomic signature of better vectors populations, differ considerably when compared to the rest of less good vector populations from the two whitefly species tested in this study. While MEAM1 efficient vector populations has a more lenient immune system, the Q efficient vector populations expressed more proteins possibly implicated in virus passage through cells. Both species show a strong link of the facultative symbiont *Rickettsia* to virus transmission efficiency.

Keywords

Bemisia tabaci, proteome, TYLC, transmission, bacterial symbiont

Data Description

The whitefly *Bemisia tabaci* is a serious threat to worldwide agriculture, yet an extensive analysis of its proteomic profile has yet been performed. The data we collected in this study represents the most extensive proteomic dataset available for this insect, or any hemipteran insect to this date. We extracted all proteins for whole insects pooled from various populations and two different species, digested them into peptides and ran them on a mass spectrometer. Three biological replicates were collected per population and three technical replicates were run at random order per biological replicate. Data is available through ProteomeXchange with identifier PXD016964 and will be a valuable tool for future research of *B. tabaci* proteins involved in virus transmission and other traits.

Potential implications

The data provided here represent the first large scale discovery proteomics data set created for *Bemisia tabaci* MEAM1 and MED species, both worldwide pests of extremely economic importance. This data was used to mine different protein expression patterns correlated with virus transmission ability. The nine populations used in this study harbor different bacterial symbionts and have varying levels of resistance to insecticides. This dataset and the identified protein patterns provide basis to study other differences at the protein level. The dataset was searched against hundreds of thousands of available whitefly sequences in the public databases, however they were not searched against the published B and Q genomes since

those exhibited tremendous differences at the assembly level and have yet to be well-annotated. We thus preferred to compare the dataset we generated against available whitefly datasets, and with other insect species for which better genome sequences are available. In the future, the dataset provided here may be searched against the assembled genomes of both studied species.

Background

Since first described more than a 100 years ago, the whitefly *Bemisia tabaci* has become an agricultural pest distributed on a worldwide scale. Its importance stems from its extreme invasiveness with international commodity trade, rapidly occupying new niches and displacing local populations, and now considered one of the most invasive species worldwide. *B. tabaci* causes direct cosmetic damage to various crops during feeding, and by the attraction of sooty mold fungus to its sugar-rich honeydew secretions [1]. Most of the damage caused by *B. tabaci* is due to virus transmission. *B. tabaci* is a vector for over 100 different viruses, primarily old and new world Begomoviruses of the family Geminiviridae but recently new viruses that belong to the Potyviridae, Closteroviridae, Luteoviridae and Betaflexiviridae were also reported to be vectored by the insect [2-4]. *B. tabaci* is a complex of morphologically indistinguishable species. However, based on sequence polymorphism in defined mitochondrial genes, it is now agreed that *B. tabaci* comprises 11 species groups, each includes some species-complex members, previously termed as biotypes [5, 6]. The two most highly polyphagous and invasive species in this complex are the Middle East Asia

Minor 1 (MEAM1 formerly known as the B biotype), and Mediterranean (MED formerly know as the Q biotype), which occupied much of our attention in the past 20 years [7, 8]. Surveys conducted over the years in Israel have reported the presence of only those two species [9].

Recently, the genomes of both MEAM1 and MED have been sequenced and published [10, 11] creating a wealth of new resources for genetic and molecular studies. *B. tabaci* genomes, which are still being annotated, is highly divergent from that of previously sequenced hemipteran species and shows vast expansions in gene families related to metabolism and insecticide resistance [10].

Mass spectrometry based proteomic approaches have become a prevalent tool in research of various biological systems - from humans to arthropods. Recent studies performed on arthropods and entomopathogenic viruses were able to isolate and identify viral structural proteins and virions from both insect cell cultures and hemolymph [12, 13]. Proteomic studies, comparing efficient and non-efficient virus vector clone lines in aphids were able to identify protein markers linked to transmission ability: in the greenbug aphid, *Schizaphis graminum,* and the *Cereal yellow dwarf virus*-RPV (CYDV-RPV) [14], and in the English grain aphid, *Sitobion avenae* that transmits *Barley yellow dwarf virus*-PAV (BYDV-PAV) [15]. Proteomic studies conducted with *B. tabaci* thus far have focused on targeting proteins or genes for the development of new insecticides [16] or for studying insecticide resistance mechanisms [17].

In this manuscript we performed a discovery mass spectrometry analysis using nine populations from the B and Q species collected in Israel and Croatia and varying in their *Tomato yellow leaf curl virus* (TYLCV) transmission ability. We compared the proteomic profiles between efficient TYLCV vector populations within each species and between the two species. We were able to identify previously undescribed proteins from *B. tabaci*, some

of which are important for virus transmission. Such candidate proteins shed more light on the molecular mechanisms that underlay the interactions taking place during TYLCV transmission by *B. tabaci*.

Analyses

TYLCV Transmission assays

To characterize our selected populations with regard to their TYLCV transmission abilities we performed several transmission experiments. We identified a gradient of transmission abilities, with MEAM1 being in general a better vector for the virus compared to Q populations (figure 1 A and B), and those results are consistent with previously published results from Israel [18]. We identified MspRQ as the most efficient vector population of the MED species (figure 1 A) and ObeRB as the most efficient MEAM1 species TYLCV vector (figure 1 B).

Proteomic analysis

We used shotgun proteomics to compare the protein expression profile of the nine different populations of the two different *B. tabaci* species collected in Israel (figure 1). Data for each population composed of 3 biological replicates and 3 technical replicates per biological one. A PCA made of all data showed low percentage of variance originating from the biological replicates, proving high reproducibility of the technical and biological replicates (figure S1, supplementary data).

We were able to identify on average 3,350 proteins from 2,510 protein families with an average FDR of 0.9% in each replicate. We then compared the expression levels of all peptides and proteins in order to identify proteins that are differentially expressed in each

TYLCV efficient vector population compared to the other populations of that species. We found that the general level of variability was much higher between the different MED populations than between the MEAM1 populations. We limited our analysis to up to 15,000 peptides showing >2-fold change in abundance. In the MEAM1 population peptides, we used only peptides with P values <0.05; this approach produced too many results in MED, therefore we reduced our analysis to peptides with P values of <0.01. This coincides with the findings showing that while MEAM1 and MED derived from the same ancestral species, during speciation, MEAM1 remained stable while MED continued to separate into more species such as MED, J, L and others [5]. Therefore, while MEAM1 populations are more unified in their proteomic profiles, Q populations are more erratic and show higher variance.

Proteins differentially expressed in both B and Q efficient TYLCV vector populations

Proteins differentially expressed in efficient TYLCV vectors from MED and MEAM1 species were compared to all other populations. Three proteins were found to be over-expressed in both; one protein of unknown function (and no known domains), a 40S ribosomal protein and an uncharacterized protein containing a sec7 domain (Figure 2). These proteins currently cannot indicate a common characteristic related to efficient TYLCV vectoring in both species.

In addition, we found five proteins that are significantly differentially expressed in the two populations, compared to all others, but also differentially expressed between the two. A catalase, a Phosphatidylethanolamine binding protein (PEBP) and Cyclophilin were over-expressed in the MED efficient-vector but down-regulated in the MEAM1 efficient-vector, while Vitellogenin and the antimicrobial protein Alo-2 were over-expressed in B while down regulated in Q efficient vectors (figure 2).

Proteins differentially expressed in B biotype efficient vector population

We examined each efficient vector population compared to other populations of the same specie and identified several interesting candidate proteins with possible functional roles in virus transmission (Figure 3). Out of 108 significantly up-regulated proteins in the efficient biotype MEAM1 vector, the proteins with more than one peptide identified with the highest expression levels were: a eukaryotic translation initiation factor 3, cathepsins B and F and a viral-A inclusion protein. Most of them were previously found to be related to pathogen transmission and infection. However, they were not found to be linked to virus acquisition and transmission by insect vectors.

We found 85 proteins with amounts significantly lower in the MEAM1 species efficient-vector compared to all other B populations (selected proteins shown in figure 3). Of these we found chondroitin proteoglycan, HSP70, Hdd11 defense protein and two cuticular proteins analogous to peritrophin (CPAP).

Proteins differentially expressed in MED biotype efficient vector population

Among the 41 significantly up regulated proteins in the MED efficient-vector compared with all other MED populations, 20 were identified as PEBPs (figure 4). Alignment of the DNA and amino acids sequences of those 20 candidates showed low sequence identity, implying that these peptides belong to different proteins of the same protein family. The MED efficient-vector population is resistant to the neonicotinoid insecticide Acetamiprid. Another MED population with similar neonicotinoid resistance also showed up regulation of a large number of PEPBs but only 3 of which are common to those up-regulated in the efficient-vector population, leaving 17 exclusively up-regulated in the efficient-vector population. No common PEBPs were differentially expressed in the MEAM1 biotype Acetamiprid-resistant population. Down-regulated proteins in the efficient TYLCV vector population included mitochondrial ribosomal and cytochrome b proteins, metabolism related enzymes such as

methionine aminopeptidase 1 and adenylate kinase 3, a heat shock factor binding protein, tubulin folding protein and more (Figure 4).

Bacterial proteins differentially expressed in TYLCV efficient-vector populations

Among the 41 common proteins highly expressed in the efficient vector populations from both specie, 37 were bacterial proteins, all from the facultative endosymbiont *Rickettsia*. *Rickettsia* has been previously implicated in virus transmission. Although each species has a different secondary endosymbiont bacterial composition, only *Rickettsia* proteins were differentially expressed in each efficient vector population. In the MEAM1 efficient-vector population a total of 53 proteins were significantly up-regulated; 37 of which common with the MED efficient-vector population, in the MED population only one *Rickettsia* protein was not shared with MEAM1. The up-regulated *Rickettsia* proteins are adhesin and other membrane proteins and transporters, GroEL and chaperonins, transcription and elongation factors, ribosomal proteins, actin polymerization protein and trigger factor proteins. Fold change of those identified proteins was higher in MED for all proteins but GroEL and adhesin proteins, whose fold change were higher in MEAM1 populations (figure 5).

In the MEAM1 efficient vector population, additional 15 *Rickettsia* proteins were significantly up regulated compared to the rest of the MEAM1 populations (Figure 6). Six of them are transcription or DNA editing related, two are membrane related proteins, two ribosomal proteins and the rest are uncategorized. One of these proteins is ftsZ, which has a crucial role in the development of the central cytoskeletal septum during cell division, strengthening our hypothesis that *Rickettsia* is dividing and proliferating more in this efficient vector population [26]. Three *Hamiltonella* proteins were down-regulated in the MEAM1 efficient vector compared to the other populations (1.98-fold change). One of them is the *Hamiltonella* GroEL protein, previously mentioned in this section. It is very surprising that

this protein, previously found to improve TYLCV transmission is down-regulated in the efficient TYLCV vector. It was hypothesized that the *Hamiltonella*'s GroEL helps the TYLCV virions to avoid the insect's immune system in the whitefly's hemolymph [18]. Our results, indicating proliferation of *Rickettsia,* could imply that the efficient vector populations' immune system is a "lenient" one, therefore TYLCV virions need not bind to *Hamiltonella*'s GroEL in order to survive the passage through the hemolymph.

Discussion

In this study, we performed the first large-scale proteomics discovery approach to identify protein expression patterns associated with virus vectoring ability by the whitefly *B. tabaci* MEAM1 and MED species. The analyses identified shared expression patterns associated with the ability of both biotypes to transmit TYLCV. The analyses further demonstrated the possible involvement of endosymbiotic bacterial proteins in the vectoring ability, confirming previous reports regarding the pivotal role of endosymbiotic bacteria in virus transmission [18, 19]. Following the examination of protein expression patterns in both efficient TYLCV vectors compared to the rest of the tested populations, we found six proteins that are significantly differentially expressed in both efficient vector populations. Catalase, Phosphatidylethanolamine binding protein (PEBP) and Cyclophilin were over-expressed in the MED efficient-vector while Vitellogenin and an antimicrobial protein Alo-2, were over expressed in MEAM1 (figure 2). Several of these proteins were previously reported with regard to virus transmission; PEBP is a gene family highly conserved with many proposed functions from lipid and cell membrane binding to signal transduction and transcription factors [20]. The Human Immunodeficiency Virus type 1 (HIV-1) was found to produce its own Cyclophillin and a PEBP named Pin1; Cyclophillin was shown to be

required for initiation of reverse transcription however the function of Pin1 remains uncertain [21]. In arthropods, a proteomic survey of *Drosophila melanogaster* hemolymph after exposure to different pathogens discovered a rise in PEBP levels after exposure to both Gram positive and negative bacteria. This result combined with previous research hypothesizing that *Drosophila* PEBP plays a role in signal transduction led to postulate that PEBPs might act in the activation of the Toll immune pathway [22]. Reumer et al. found a specific PEBP whose constitutive up-regulation made *Drosophila* larvae more resistant to bacterial infection while a knock out made them hyper sensitive [23]. The recent sequencing of the MEAM1 biotype genome showed that PEBP genes are more than 10-fold more abundant in the *B. tabaci* MEAM1 genome compared to 15 other arthropod genomes [10]. This finding, along with our data hints on the important role this gene family has in whiteflies, where they are likely participating in various processes, likely including virus transmission.

Cyclophilin, a peptydil prolyl-isomerase, was shown to be linked to CYDV-RPV transmission in *Rhopalosiphum padi* aphids. Cyclophilin was found to be up-regulated in efficient-vector clone lines compared to inefficient-vector lines. It was also shown to bind to CYDV-RPV virions [24]. Different isoforms of the protein were shown to segregate between clones with different CYDV-RPV transmission efficiencies [25]. Three cyclophilin genes were identified in *B. tabaci* MEAM1 species. The expression of one of them was shown to be induced upon TYLCV infection, in the whitefly midgut [26]. TYLCV CP and cyclophilin were shown to co-localize in *B. tabaci* midguts and ovaries. Finally, feeding whiteflies with cyclophilin antibodies, a cyclophilin inhibitor Cyclosporin A or dsRNA of cyclophilin greatly reduced TYLCV transmission rates [26, 27].

The over expression of these two proteins in the efficient-vector populations confirmed the validity of our approach regarding the MED efficient-vector population. It was surprising, however, that they were significantly down regulated in the MEAM1 efficient-

vector population. Alo-2 is a protein of the Knottin family, a highly diverse protein family with one common domain; the knottin fold. Knottin proteins are extensively studied in arthropods such as *Drosophila* and various Coleopterans with regards to the systemic immune response. Members of the Knottin family have been described to have antifungal and antibacterial functions [28, 29], while no antiviral function was yet identified. Alo-2 is likely to function in the immune response of *B. tabaci* and therefore it's upregulation in the efficient vector is unexpected.

Vitellogenin, a large phospholipoglycoprotein involved in oogenesis and presumed to be a storage nutrient in the yolk was highly upregulated in both male and juvenile fish from various species. This later finding led to the conclusion that it functions as a hemaglutinating factor and an antibacterial effector in organisms from multiple kingdoms [30, 31].

Wei et al. (2017), demonstrated the crucial role of Vitellogenin in transovarial transmission of TYLCV in *B. tabaci* MEAM1 species, thus putting an end to a long-standing debate on the subject [32-35]. Wei et al. have shown that Vitellogenin binds to TYLCV coat protein and aids virus translocation into developing eggs in the ovaries. Interestingly, this study showed that TYLCV was transovarially transmitted to eggs in mature females (11 days after emergence) significantly more efficiently than in young females (1 day after emergence). All samples collected for our study were 1-5 days after emergence, a life stage indicated to have lower TYLCV transovarial transmission efficiency, however we find elevated levels of Vitellogenin in both MEAM1 and MED efficient vector populations. Interestingly, we found in the current study that peptides spanning the entire vitellogenin sequence were up regulated in the MEAM1 efficient vector while in the MED, peptides from a certain region of the protein only were found to be down regulated, not the entire protein (figure S2). This could hint to the existence of different isoforms of vitellogenin in the two species, with different

functional roles.

When each efficient vector population was compared with other populations within the same specie we found a eukaryotic translation initiation factor 3, Cathepsins B and F, and a viral-A inclusion protein. Most of which were studied before in relation to pathogen transmission and infection. Cathepsins are a large family of proteases, in arthropods they are primarily expressed in the digestive system. Both Cathepsins identified in this research- B and F are Cystein proteases. Although phloem-sap does not contain protein levels high enough to fulfill the dietary needs of phloem feeding insects such as aphids and whiteflies, Cathepsins have been identified in several aphids [36]. The Cathepsin B gene family in the pea aphid, *Acyrthosiphon pisum*, was found to include 28 different genes indicating the presence of a positive selection pressure leading to the expansion of this family. It is postulated that these proteins might be excreted into the plant phloem or that they may assist in resistance to plant defensive secondary metabolites found in the plant sap [37].

We found 85 proteins with concentrations significantly lower in the MEAM1 efficient-vector compared to all other BMEAM1 populations (selected proteins shown in figure 3). Of these proteins we found Chondroitin proteoglycan, HSP70, Hdd11 defense protein and two cuticular proteins analogous to peritrophin (CPAP). All of which were previously studied in relation to virus transmission or immune responses. Except for Chondroitin proteoglycan, all the other four proteins are known as virus transmission inhibitors; Hdd11 and CPAP are related to immune system and HSP70 was previously shown in whiteflies to inhibit TYLCV passage through the insect midgut epithelial cells [38-40]. It is therefore logical that those proteins were down regulated in the efficient-vector, demonstrating a more “relaxed” immune system in which TYLCV virions have more chances of making a full passage through the whitefly tissues and insure successful transmission.

However, Chondroitin proteoglycan was previously described with regard to Malaria infection; a population of Chondroitin sulfate proteoglycans were located on the microvilli epithelial cells of the apical midgut, the salivary glands and ovaries of the arthropod vector *Anopheles gambiae* [41]. *In vitro* experiments showed recognition of the protein by *Plasmodium falciparum* ookinetes. In addition, RNAi-mediated silencing of Chondroitin proteoglycan significantly lowered the percentage of matured ookinetes in live mosquitoes [42]. Research performed in *Bombyx mori* showed that Baculoviruses bind and digest Chondroitin proteoglycans in order to destroy the peritrophic membrane (PM) and enter the midgut epithelial cells of the insect host [43]. These results may imply a role of chondroitin proteoglycans in virus binding to the midgut membrane, such an association has not been shown for TYLCV-*B. tabaci* and this down regulation of the protein in the B efficient-vector makes it less likely.

Among the 41 significantly up regulated proteins in the MED efficient-vector compared to all other tested MED populations, 20 were recognized as PEBPs (figure 4). PEBPs were found to be linked to immune response activation against bacterial infection in *Drosophila*, however, it was also found to be necessary for HIV1 infection [22, 23]. Other prominent up regulated proteins include a protein with a RUN and FYVE domain, a vesicle associated membrane protein, glutathione peroxidase and mucin-2 like protein. All these proteins were previously linked to different pathogens' membrane binding and cell invasion abilities. FYVE domain functions in membrane trafficking [44]. A FYVE containing phosphatidylinositol-3-phosphate in mammals was found to be a binding site initiating endocytosis and cell invasion of *Vesicular stomatitis virus*. Inhibition of the FYVE domain of the protein inhibited infection [45]. In arthropods, a FYVE domain containing a zinc-finger was found up regulated in *Litopenaeus vannamei* shrimp resistant to *Taura syndrome virus*, although less than 2-fold compared to the susceptible [46]. This protein along with the vesicle

associated membrane protein may act in the movement of TYLCV virions through *B. tabaci* midgut and salivary gland cells.

A mucin-like protein was associated with the passage of *Plasmodium* through the guts of the mosquito *Aedes aegypti* [47]. It is also a possible target protein of Baculoviruses while crossing the plasma membrane of the arthropod host [48]. Down regulated proteins in this population included Cytochrome b, which was found to be down regulated in *Anopheles gambiae* midguts after acquisition of O'nyong-nyong Virus [49]. The significantly different levels of all these proteins in this population versus the rest of the MED populations in this study suggest that it has a midgut specificity; it is more permeable and thus TYLCV circulation is more efficient. The gut barrier was found to be the first and often most important barrier for an insect- transmitted pathogen to cross, especially in the whitefly-begomovirus interaction [50].

Among the 41 common proteins highly expressed in the efficient vector population from both species, 37 were bacterial proteins encoded by *Rickettsia*. *Rickettsia* is the only shared secondary endosymbiont between MED and MEAM1 in Israel [9]. Although not all populations tested in this experiment harbor *Rickettsia*, its protein levels were higher in the two efficient vector populations, compared to the other populations. This result indicates that the general level of *Rickettsia* is higher in the MEAM1 efficient vector than in the rest of the populations while in MED it might not be the case. There might not be higher titers of the bacterium in neither of the efficient vector populations, however, the bacterium might be indirectly regulating processes in both populations. The up-regulated *Rickettsia* proteins are adhesin and other membrane proteins and transporters, GroEL and chaperonins, transcription and elongation factors, ribosomal proteins, actin polymerization protein and trigger factor proteins. High levels of proteins from all these groups indicates that the bacteria are

propagating and undergoing cell division characteristic of a 'log phase' of bacterial growth. For example; *Escherichia coli* Trigger factor protein was found to act as a ribosomal mediated chaperone critical for protein folding along with DnaK which is related to protein excretion and also found to function as a peptidyl-prolyl cis-trans isomerase (cyclophilin) *in vitro* [51, 52]. In *E. coli*, Trigger factor binds to the 50S ribosome at the N terminal of the L23 subunit [53]. We found subunits L7,10,12,21 up regulated but not L23, however, combined with the rise in DnaK and trigger factor, our data implies a certain upregulation in protein assembly. Other chaperone proteins, including GrpE, identified in our data, were shown to be up-regulated during cell division [54]. The highly upregulated Actin polymerization protein we identified is related to the cytoskeletal reassembly during cell division [55]. Finally, the up-regulation of several cell surface and membrane proteins as well as adhesin indicates that *Rickettsia* interact with its host cells, perhaps infecting more of them [56].

The proteins with highest fold change are a trigger factor (Infinitely different in MED- meaning it was not found in certain MED populations), DnaK, Cold shock protein CspA, co-chaperone GrpE, a membrane protein, PEBP, a 50S ribosomal protein, a transcription elongation factor GreA (extremely up regulated in MED) and an actin polymerization protein RickA (Figure 5).

The role of bacterial endosymbionts in plant virus transmission is still under debate [64]. However, we have previously demonstrated that TYLCV transmission depends upon *Hamiltonella defensa*, a secondary symbiont of *B. tabaci*. TYLCV virions were shown to bind to *Hamiltonella* GroEL ensuring safe movement in the insect hemolymph thus evading the immune system of the vector on the transmission pathway [18]. The presence of such a high number of up-regulated *Rickettsia* GroEL and chaperone proteins, specifically the trigger factor that is highly up regulated in MED, may indicate a possible substitution of

Hamiltonella GroEL, specifically in the MED efficient-vector, which does not harbor *Hamiltonella*, but still retains high vectoring abilities. Previous research found no interaction between TYLCV and *Rickettsia* GroEL [18]. However, there could be an interaction between TYLCV and other *Rickettsia* chaperonins. It should be noted that the only other *Rickettsia* protein up regulated in MED is an additional trigger factor.

Conclusions

The results obtained in this study indicate that making a better vector population in each species of *B. tabaci* is influenced by many factors. In MEAM1 species, among those factors are the down-regulation of immune-related virus transmission inhibitors, while in MED it is derived from up-regulation of possible target proteins of the virus in the midgut. Surprisingly, most common proteins differentially expressed in both efficient vector populations showed different expression patterns between the two species. Among only 4 proteins up-regulated in both populations, three are of unknown function, although one has a known sec7 domain, and the fourth is a ribosomal protein. The only group of proteins common to both efficient vector populations of both species was the one encoded by *Rickettsia*, and those proteins were up-regulated in both species. We have previously shown, with another population of *B. tabaci* MEAM1 species, that *Rickettsia* is implicated in making *B. tabaci* a better vector for TYLCV. In this study, we showed that the presence of *Rickettsia* increased the transmission rates by two fold, and enhanced both acquisition and retention of the virus [19]. Our current study shows in efficient vector populations elevated levels of vitellogenin, a protein recently implicated in transovarial transmission of TYLCV. We previously demonstrated high levels of vitellogenin and high fecundity associated with the presence of *Rickettsia* (Brumin & Ghanim, unpublished data). Taking together, these results point out to another possible effect of this bacterium on TYLCV transmission. Six of the nine populations tested in this

experiment were infected with *Rickettsia* (see table 1), however, no correlation was found between the presence of *Rickettsia* and TYLCV transmission efficiency, as previously published for another secondary symbiont of *B. tabaci, Hamiltonella* [18]. This could indicate that infection with the bacterium is not enough to improve transmission ability; the expression of additional genes from the bacterium are needed to impact virus transmission.

Methods

Insect collections and rearing in the lab

B. tabaci populations were collected from various locations in Israel and Croatia (table 1) and reared on cotton seedlings (*Gossypium hirsutum* L. cv. Acala) in insect proof cages maintained in growth rooms under standard conditions of 25°C±2°C, 60% relative humidity, and a 14-h light/10-h dark photoperiod. Three to five replicates containing 200-500 individuals were collected from each population up to a week after adult emergence. Samples were placed at -80°C till samples from all populations were collected.

Virus transmission assays

In order to calculate TYLCV transmission efficiencies of whitefly populations, 6-7 days old adults from each population were given a 48-h acquisition access period (AAP) on a TYLCV-infected tomato plant. The insects were then used for a 7-day inoculation access period (IAP) on 4-week-old, non-infected tomato plants, one whitefly per plant- in leaf clip cages. Two weeks post inoculation, young leaves were collected from the plants for DNA extraction (using the Dellaporta protocol, detailed in [57]) and PCR for TYLCV detection (using primers listed in [18]). Three replicates of 30 plants were performed for each

population (except the Q-AWR population that was terminated after the first assay due to technical problems).

Protein extractions and preparations for MS analysis

Protein were extracted as described in [58]; samples were grinded using a mortar and pestle while kept frozen using liquid nitrogen. 1ml of 10% TCA-acetone, 2% β-mercaptoethanol was added per sample. Samples were then incubated for 16 h at -20°C, then centrifuged at 5000 x *g*, 4°C, 30 minutes. Pellets were saved and washed 3 times with cold acetone, dried and re-suspended in 8M urea in 100mM ABC.

Protein was quantified using a Bradford assay, protein integrity was examined by running 5µg from each sample on 1D gel with BSA as a control, and a Coomassie Brilliant Blue staining.

Protein samples then proceeded to reduction, Cystein blocking and Trypsin digestion- 50 µg of protein was added to a final volume of 10 mM of DTT in 100 mM ABC, samples were then incubated at 30°C for an hour. A final volume of 30 mM of MMTS in 100 mM ABC was added and samples were incubated for one hour in room temperature.

Samples were then diluted to ~1M urea with 100 mM ABC and Trypsin was added in a 1:50 ratio (Trypsin:protein). Samples were incubated for 16 h at 37°C, desalted using Waters Sep Pak SPE cartridges (according to manufacturer's protocol), dried and kept at -80°C till MS analyses.

MS runs

Dried protein pellets were sonicated for five minutes in 0.2% TFA 2% acetonitrile, strongly vortexed one minute, and vortexed further at 1,200 rpm at 37°C for at least 10 minutes. Samples given a hard spin to pellet particles prior to placing in auto-sampler vials, and 3 µl

injected (i.e., 50 µg/ 75 µl x 3 µl loaded to trap). Jupiter C12 5-micron beads were used to make a 2 cm x 150 micron trap prior to a 30 cm x 75 micron Dr Maisch 3 micron C18 packed tip. Samples were analyzed on a ThermoScientific Orbitrap in the MacCoss lab (University of Washington) using a top 10 DDA method. Samples were analyzed in triplicate and randomized with a blank run every third injection. MS data were deposited to the ProteomeXchange consortium via PRIDE [59] with identifier PXD016964.

MS analysis and data annotations

An initial search of all animal protein sequences on NCBI (monthly) showed approximately 1,000 proteins identified per run. A FASTA database of whitefly and whitefly endosymbiont bacterial DNA sequences from NCBI was compiled and used for Mascot searching. Using this as a database, the search was drastically improved, with an average of 3350.5 peptides being matched per LCMS run, with an average FDR of 0.9%. Percolator [60,61] was used for correcting for multiple hypothesis testing and computing q-values. Mascot files were then loaded into the Progenesis QI program (Nonlinear) and aligned to a randomly selected reference run. Each and every run was then aligned and problematic regions with low alignment were removed. The data was then analyzed 3 times, once for every technical replicate of every biological replicate. Hence the data was analyzed as three separate experiments, each containing three biological replicates for every population and one technical replicate of each. Average normalized abundance was calculated for each protein, based on protein features without conflict only, and fold change between the highest and lowest values was calculated. All peaks were then compared and only those showing a >2-fold in expression with P values <0.05 in B and <0.01 in Q were selected for analysis. Further sifting of the data was done to keep only proteins that had at least one unique peptide

sequence identified. All three final list of proteins, from the three technical replicates, were then compared and only proteins that appeared in at least two of the three were kept.

Funding

This work was funded by a Binational Agricultural Research and Development (BARD) travel grant to Adi Kliot.

Authors' contributions

AK- Investigation, Formal analysis, Validation, Visualization, Writing- original draft, Funding Acquisition

MM- Formal analysis

RJ- Formal analysis

GL- Resources

SK- Resources

HC- Supervision, Writing- review & editing

MG- Conceptualization, Supervision, Writing- review & editing

MH- Methodology, Resources, Validation, Data Curation, Funding Acquisition, Writing- review & editing

References

1. Oliveira M, Henneberry T and Anderson P. History, current status, and collaborative research projects for *Bemisia tabaci*. Crop protection. 2001;20 9:709-23.
2. Jones DR. Plant viruses transmitted by whiteflies. European Journal of Plant Pathology. 2003;109 3:195-219.
3. Navas-Castillo J, Fiallo-Olivé E and Sánchez-Campos S. Emerging virus diseases transmitted by whiteflies. Annual Review of Phytopathology. 2011;49:219-48.
4. Ghosh S, Kanakala S, Lebedev G, Kontsedalov S, Silverman D, Alon T, et al. Transmission of a new polerovirus infecting pepper by the whitefly Bemisia tabaci. Journal of virology. 2019:JVI. 00488-19.
5. De Barro PJ, Liu S-S, Boykin LM and Dinsdale AB. Bemisia tabaci: a statement of species status. Annual review of entomology. 2011;56:1-19.
6. Liu S-s, Colvin J and De Barro PJ. Species Concepts as Applied to the Whitefly *Bemisia tabaci* Systematics: How Many Species Are There? Journal of Integrative Agriculture. 2012;11 2:176-86.
7. Gennadius P. Disease of tobacco plantations in the Trikonia. The aleurodid of tobacco. Ellenike Georgia. 1889;5:1-3.
8. Brown J, Frohlich D and Rosell R. The sweetpotato or silverleaf whiteflies: biotypes of Bemisia tabaci or a species complex? Annual review of entomology. 1995;40 1:511-34.
9. Chiel E, Gottlieb Y, Zchori-Fein E, Mozes-Daube N, Katzir N, Inbar M, et al. Biotype-dependent secondary symbiont communities in sympatric populations of Bemisia tabaci. Bulletin of Entomological Research. 2007;97 04:407-13.
10. Chen W, Hasegawa DK, Kaur N, Kliot A, Pinheiro PV, Luan J, et al. The draft genome of whitefly Bemisia tabaci MEAM1, a global crop pest, provides novel

insights into virus transmission, host adaptation, and insecticide resistance. BMC biology. 2016;14 1:110.

11. Xie W, Chen C, Yang Z, Guo L, Yang X, Wang D, et al. Genome sequencing of the sweetpotato whitefly Bemisia tabaci MED/Q. GigaScience. 2017;6 5:1-7.

12. Franco CF, Mellado MCM, Alves PM and Coelho AV. Monitoring virus-like particle and viral protein production by intact cell MALDI-TOF mass spectrometry. Talanta. 2010;80 4:1561-8.

13. Tsai J-M, Wang H-C, Leu J-H, Hsiao H-H, Wang AH-J, Kou G-H, et al. Genomic and proteomic analysis of thirty-nine structural proteins of shrimp white spot syndrome virus. Journal of virology. 2004;78 20:11360-70.

14. Cilia M, Peter KA, Bereman MS, Howe K, Fish T, Smith D, et al. Discovery and targeted LC-MS/MS of purified polerovirus reveals differences in the virus-host interactome associated with altered aphid transmission. 2012, PLoS One, 7:e48177.

15. Papura D, Jacquot E, Dedryver C, Luche S, Riault G, Bossis M, et al. Two-dimensional electrophoresis of proteins discriminates aphid clones of Sitobion avenae differing in BYDV-PAV transmission. Archives of virology. 2002;147 10:1881-98.

16. Mishra M, Saurabh S, Maurya R, Mudawal A, Parmar D and Singh PK. Proteome analysis of Bemisia tabaci suggests specific targets for RNAi mediated control. Journal of proteomics. 2016;132:93-102.

17. Yang N, Xie W, Yang X, Wang S, Wu Q, Li R, et al. Transcriptomic and Proteomic Responses of Sweetpotato Whitefly, *Bemisia tabaci*, to Thiamethoxam. PLoS ONE. 2013;8 5:e61820. doi:10.1371/journal.pone.0061820.

18. Gottlieb Y, Zchori-Fein E, Mozes-Daube N, Kontsedalov S, Skaljac M, Brumin M, et al. The transmission efficiency of tomato yellow leaf curl virus by the whitefly

Bemisia tabaci is correlated with the presence of a specific symbiotic bacterium species. Journal of virology. 2010;84 18:9310-7.

19. Kliot A, Cilia M, Czosnek H and Ghanim M. Implication of the Bacterial Endosymbiont Rickettsia spp. in Interactions of the Whitefly Bemisia tabaci with Tomato yellow leaf curl virus. Journal of virology. 2014;88 10:5652-60.

20. Banfield MJ, Barker JJ, Perry AC and Brady RL. Function from structure? The crystal structure of human phosphatidylethanolamine-binding protein suggests a role in membrane signal transduction. Structure. 1998;6 10:1245-54.

21. Ott DE, Coren LV, Johnson DG, Kane BP, Sowder RC, Kim YD, et al. Actin-binding cellular proteins inside human immunodeficiency virus type 1. Virology. 2000;266 1:42-51.

22. Levy F, Rabel D, Charlet M, Bulet P, Hoffmann JA and Ehret-Sabatier L. Peptidomic and proteomic analyses of the systemic immune response of Drosophila. Biochimie. 2004;86 9:607-16.

23. Reumer A, Bogaerts A, Van Loy T, Husson SJ, Temmerman L, Choi C, et al. Unraveling the protective effect of a Drosophila phosphatidylethanolamine-binding protein upon bacterial infection by means of proteomics. Developmental & Comparative Immunology. 2009;33 11:1186-95.

24. Yang X, Thannhauser T, Burrows M, Cox-Foster D, Gildow FE and Gray SM. Coupling genetics and proteomics to identify aphid proteins associated with vector-specific transmission of polerovirus (Luteoviridae). Journal of virology. 2008;82 1:291-9.

25. Tamborindeguy C, Bereman MS, DeBlasio S, Igwe D, Smith DM, White F, et al. Genomic and proteomic analysis of Schizaphis graminum reveals cyclophilin proteins

are involved in the transmission of Cereal yellow dwarf virus. PloS one. 2013;8 8:e71620.

26. Kanakala S and Ghanim M. Implication of the whitefly Bemisia tabaci cyclophilin B protein in the transmission of Tomato yellow leaf curl virus. Frontiers in plant science. 2016;7:1702.

27. Kanakala S, Kontsedalov S, Lebedev G and Ghanim M. Plant-Mediated Silencing of the Whitefly Bemisia tabaci Cyclophilin B and Heat Shock Protein 70 impairs insect development and virus transmission. Frontiers in physiology. 2019;10:557.

28. Ntwasa M, Goto A and Kurata S. Coleopteran antimicrobial peptides: prospects for clinical applications. International journal of microbiology. 2012;2012.

29. Balmand S, Vallier A, Vincent-Monégat C, Vigneron A, Weiss-Gayet M, Rochat D, et al. Antimicrobial peptides keep insect endosymbionts under control. Science. 2011;334 6054:362-5.

30. Zhang S, Sun Y, Pang Q and Shi X. Hemagglutinating and antibacterial activities of vitellogenin. Fish & shellfish immunology. 2005;19 1:93-5.

31. Suzuki S, Oshima K, Kakizawa S, Arashida R, Jung H-Y, Yamaji Y, et al. Interaction between the membrane protein of a pathogen and insect microfilament complex determines insect-vector specificity. Proceedings of the National Academy of Sciences. 2006;103 11:4252-7.

32. Rubinstein G and Czosnek H. Long-term association of tomato yellow leaf curl virus with its whitefly vector Bemisia tabaci: effect on the insect transmission capacity, longevity and fecundity. Journal of General Virology. 1997;78 10:2683-9.

33. Cohen S and Nitzany F. Transmission and host range of the tomato yellow leaf curl virus. Phytopathology. 1966;56 10:1127-31.

34. Ghanim M, Morin S, Zeidan M and Czosnek H. Evidence for Transovarial Transmission of Tomato Yellow Leaf Curl Virus by Its Vector, the WhiteflyBemisia tabaci. Virology. 1998;240 2:295-303.

35. Wei J, He Y-Z, Guo Q, Guo T, Liu Y-Q, Zhou X-P, et al. Vector development and vitellogenin determine the transovarial transmission of begomoviruses. Proceedings of the National Academy of Sciences. 2017;114 26:6746-51.

36. Zhu-Salzman K and Zeng R. Insect Response to Plant Defensive Protease Inhibitors. Annual review of entomology. 2015;60:233-52.

37. Rispe C, Kutsukake M, Doublet V, Hudaverdian S, Legeai F, Simon J-C, et al. Large gene family expansion and variable selective pressures for cathepsin B in aphids. Molecular biology and evolution. 2008;25 1:5-17.

38. Götz M, Popovski S, Kollenberg M, Gorovits R, Brown JK, Cicero JM, et al. Implication of *Bemisia tabaci* heat shock protein 70 in begomovirus-whitefly interactions. Journal of virology. 2012;86 24:13241-52.

39. Bao Y-Y, Wang Y, Wu W-J, Zhao D, Xue J, Zhang B-Q, et al. De novo intestine-specific transcriptome of the brown planthopper *Nilaparvata lugens* revealed potential functions in digestion, detoxification and immune response. Genomics. 2012;99 4:256-64.

40. Wang L, Li F, Wang B and Xiang J. A new shrimp peritrophin-like gene from Exopalaemon carinicauda involved in white spot syndrome virus (WSSV) infection. Fish & shellfish immunology. 2013;35 3:840-6.

41. Sinnis P, Coppi A, Toida T, Toyoda H, Kinoshita-Toyoda A, Xie J, et al. Mosquito heparan sulfate and its potential role in malaria infection and transmission. Journal of Biological Chemistry. 2007;282 35:25376-84.

42. Dinglasan RR, Alaganan A, Ghosh AK, Saito A, van Kuppevelt TH and Jacobs-Lorena M. Plasmodium falciparum ookinetes require mosquito midgut chondroitin sulfate proteoglycans for cell invasion. Proceedings of the National Academy of Sciences. 2007;104 40:15882-7.

43. Sugiura N, Ikeda M, Shioiri T, Yoshimura M, Kobayashi M and Watanabe H. Chondroitinase from baculovirus *Bombyx mori* nucleopolyhedrovirus and chondroitin sulfate from silkworm *Bombyx mori*. Glycobiology. 2013:cwt082.

44. Leevers SJ, Vanhaesebroeck B and Waterfield MD. Signalling through phosphoinositide 3-kinases: the lipids take centre stage. Current opinion in cell biology. 1999;11 2:219-25.

45. Le Blanc I, Luyet P-P, Pons V, Ferguson C, Emans N, Petiot A, et al. Endosome-to-cytosol transport of viral nucleocapsids. Nature cell biology. 2005;7 7:653-64.

46. Sookruksawong S, Sun F, Liu Z and Tassanakajon A. RNA-Seq analysis reveals genes associated with resistance to Taura syndrome virus (TSV) in the Pacific white shrimp *Litopenaeus vannamei*. Developmental & Comparative Immunology. 2013;41 4:523-33.

47. Berois M, Romero-Severson J and Severson D. RNAi knock-downs support roles for the mucin-like (AeIMUC1) gene and short-chain dehydrogenase/reductase (SDR) gene in *Aedes aegypti* susceptibility to *Plasmodium gallinaceum*. Medical and veterinary entomology. 2012;26 1:112-5.

48. Rohrmann GF. The baculovirus replication cycle: Effects on cells and insects. 2013.

49. Rider MA, Zou J, Vanlandingham D, Nuckols JT, Higgs S, Zhang Q, et al. Quantitative Proteomic Analysis of the *Anopheles gambiae* (Diptera: Culicidae) Midgut Infected With O'nyong–Nyong Virus. Journal of medical entomology. 2013;50 5:1077-88.

50. Pan L, Chen Q, Guo T, Wang X, Li P, Wang X, et al. Differential efficiency of a begomovirus to cross the midgut of different species of whiteflies results in variation of virus transmission by the vectors. Science China Life Sciences. 2018:1-12.

51. Deuerling E, Schulze-Specking A, Tomoyasu T, Mogk A and Bukau B. Trigger factor and DnaK cooperate in folding of newly synthesized proteins. Nature. 1999;400 6745:693-6.

52. Teter SA, Houry WA, Ang D, Tradler T, Rockabrand D, Fischer G, et al. Polypeptide flux through bacterial Hsp70: DnaK cooperates with trigger factor in chaperoning nascent chains. Cell. 1999;97 6:755-65.

53. Hesterkamp T, Hauser S, Lütcke H and Bukau B. *Escherichia coli* trigger factor is a prolyl isomerase that associates with nascent polypeptide chains. Proceedings of the National Academy of Sciences. 1996;93 9:4437-41.

54. Cohen DP, Renes J, Bouwman FG, Zoetendal EG, Mariman E, de Vos WM, et al. Proteomic analysis of log to stationary growth phase Lactobacillus plantarum cells and a 2-DE database. Proteomics. 2006;6 24:6485-93.

55. Bramhill D. Bacterial cell division. Annual review of cell and developmental biology. 1997;13 1:395-424.

56. Klemm P and Schembri MA. Bacterial adhesins: function and structure. International Journal of Medical Microbiology. 2000;290 1:27-35.

57. Cilia M, Fish T, Yang X, McLaughlin M, Thannhauser T and Gray S. A comparison of protein extraction methods suitable for gel-based proteomic studies of aphid proteins. Journal of biomolecular techniques: JBT. 2009;20 4:201.

58. Brosch M, Yu L, Hubbard T and Choudhary J. Accurate and sensitive peptide identification with Mascot Percolator. Journal of proteome research. 2009;8 6:3176-81.

59. Perez-Riverol Y, Csordas A, Bai J, Bernal-Llinares M, Hewapathirana S, Kundu DJ, Inuganti A, Griss J, Mayer G, Eisenacher M and Pérez E. The PRIDE database and related tools and resources in 2019: improving support for quantification data. Nucleic acids research. 2018;47(D1):D442-50.

60. Käll L, Canterbury JD, Weston J, Noble WS and MacCoss MJ. Semi-supervised learning for peptide identification from shotgun proteomics datasets. Nature methods. 2007;4 11:923-5.

61. Spivak M, Weston J, Bottou L, Käll L and Noble WS. Improvements to the percolator algorithm for Peptide identification from shotgun proteomics data sets. Journal of proteome research. 2009;8 7:3737-45.

Figure Legends

Figure 1. TYLCV Transmission abilities of MED (A) and MEAM1 (B) biotype populations used in this study. MspRQ and OberRB are the populations with the highest transmission efficiency in each species. Numbers above columns represent the number of plants tested for virus transmission with whiteflies from each population.

Figure 2. Proteins of differential expression common to both efficient vector populations show opposite expression levels. Common proteins significantly differentially expressed in MEAM1 (dark gray) and MED (light gray) efficient vectors.

Figure 3. Top 20 differentially expressed proteins in OberRB. The 20 proteins with significantly low expression levels and the 20 proteins with significantly high expression levels in the MEAM1 efficient vector population compared to all other MEAM1 populations.

Figure 4. Top 20 differentially expressed proteins in MspRQ. The 20 proteins with significantly low expression levels and the 20 proteins with significantly high expression levels in the MED efficient vector population compared to the other MED populations.

Figure 5. *Rickettsia* proteins are expressed at high levels in both B and MED efficient vector populations. Common bacterial proteins significantly over expressed in MEAM1 (black) and MED (white) efficient vectors. * indicates expression levels infinitely higher than other populations (protein not identified at all in certain populations).

Figure 6. Additional *Rickettsia* proteins upregulated in the MEAM1 efficient vector population.

Figure S1. Peptide PCAs for select populations. Two PCA analyses for three randomly selected populations (of 9 in the experiment- three in A and another three in B). Data for each PCA consisted of quantification of all peptides found in all three biological replicates done for each population and all three technical replicates done per biological replicate.

Figure S2. *B. tabaci* complete Vitellogenin amino acids sequence. Highlighted are peptides identified to be up-regulated in the B efficient TYLCV vector compared to the rest of the B populations. Formatted are the peptides identified to be down-regulated in the Q efficient vector compared to the rest of Q.

Table 1. Populations collected and used in this study

	Symbiont populations composition						
**population name**	**P**	**H**	**A**	**W**	**R**	**C**	**collection site**
**Q populations**							
Q-AWR	+		+	+	+		Ayalon valley, Israel
fluf	+		+	+	+		Israel
Zadar	+	+	+	+			Zadar, Croatia
Q'-HC	+	+				+	Croatia
MspRQ	+		+	+	+		Israel
**B populations**							
Ayalon	+	+			+		Ayalon Valley, Israel
MspRB	+	+					Israel
Tamra	+	+			+		Tamra, Israel
ObeRB	+	+			+		Israel

Figure 1

Click here to access/download;Figure;Figure 1.tif

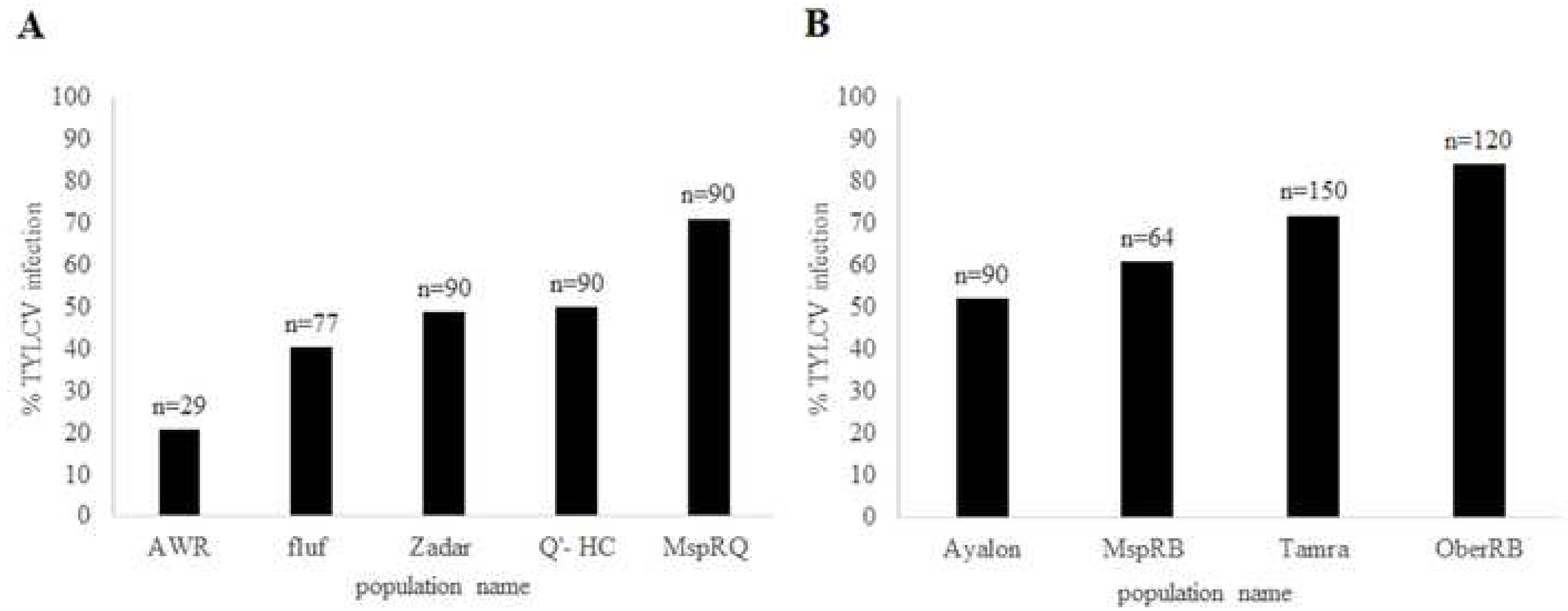

Figure 2

Click here to access/download;Figure;Figure 2.tif

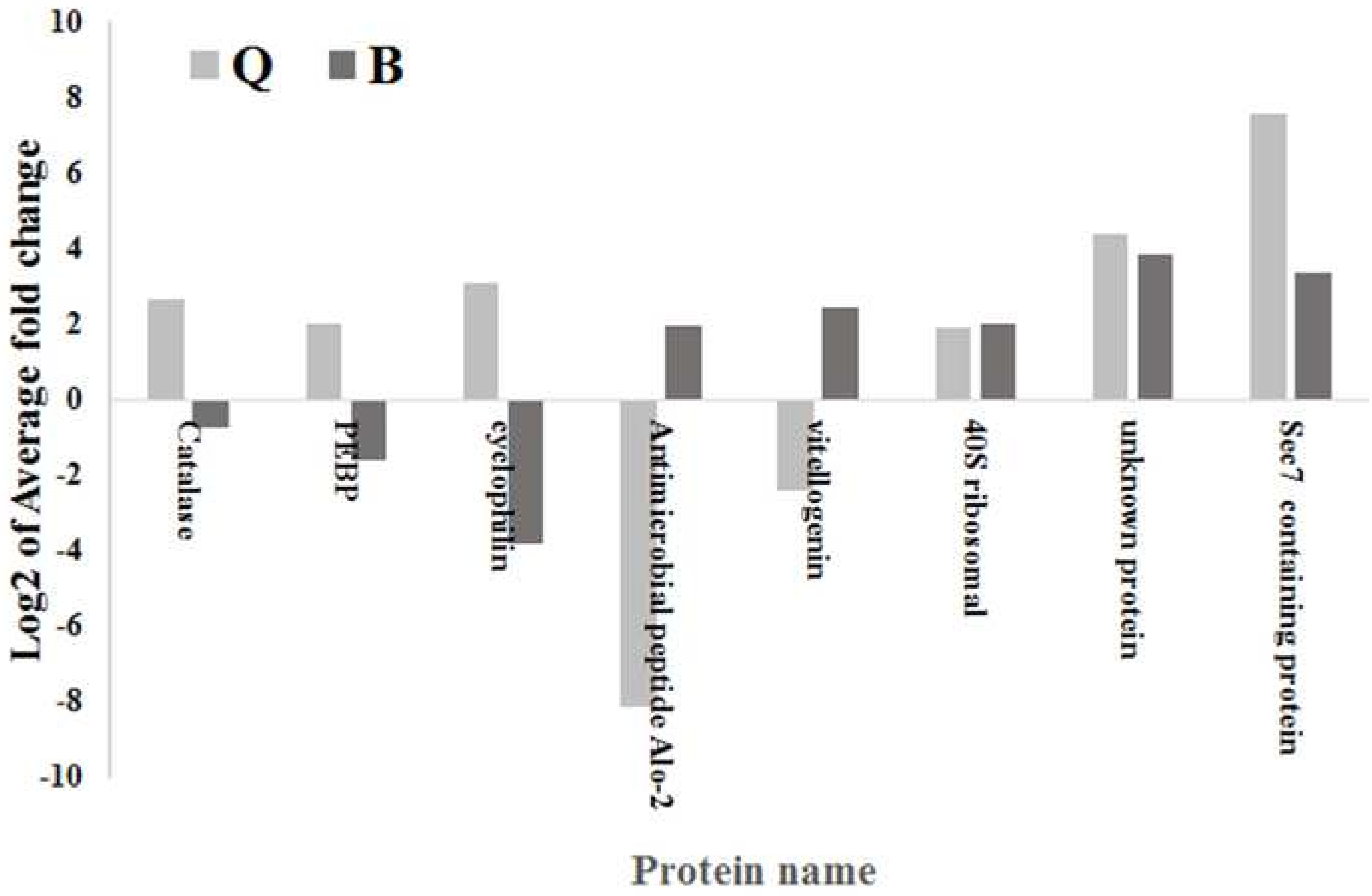

Figure 3

Click here to access/download;Figure;Figure 3.tif

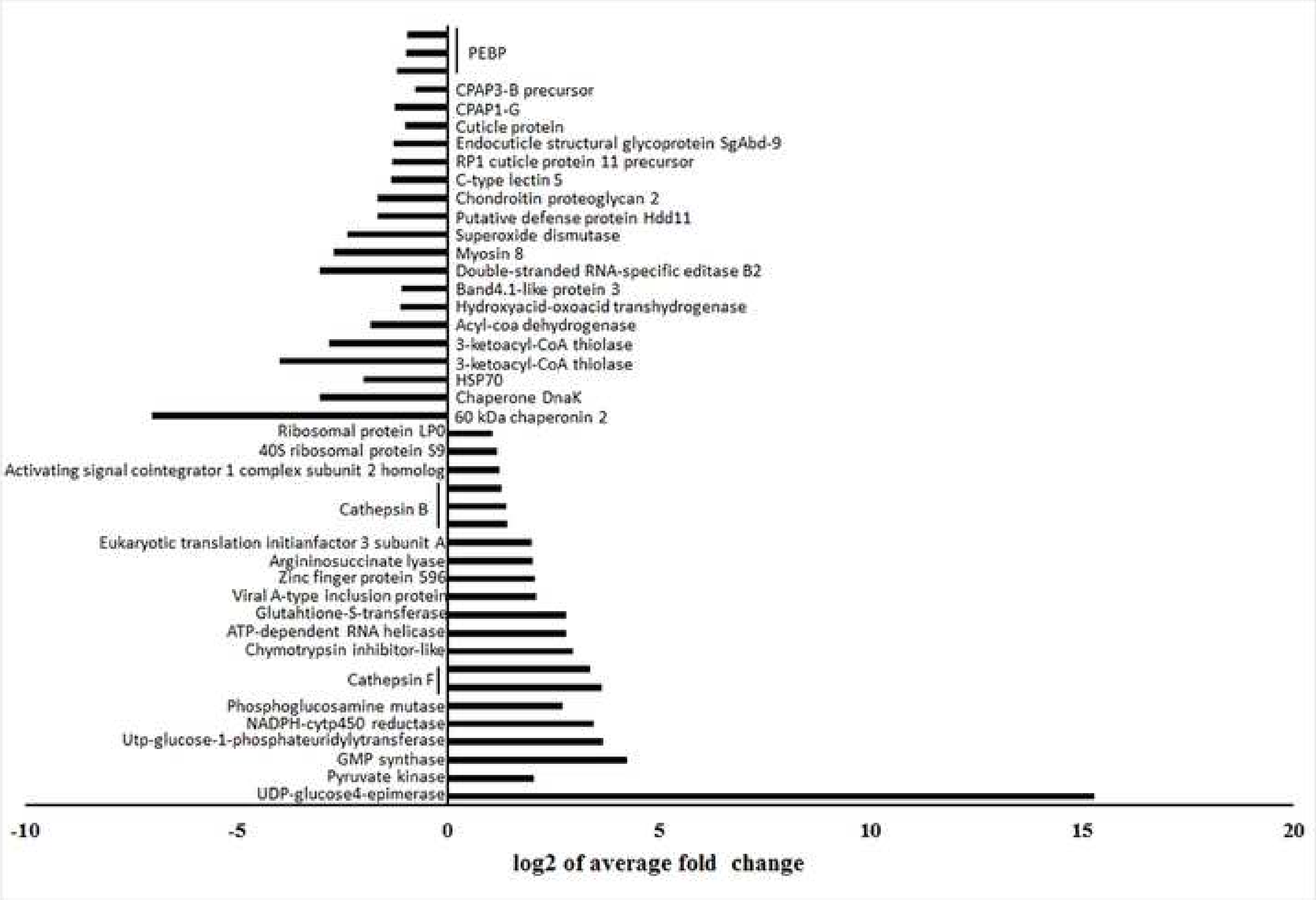

Figure 4

Click here to access/download;Figure;Figure 4.tif

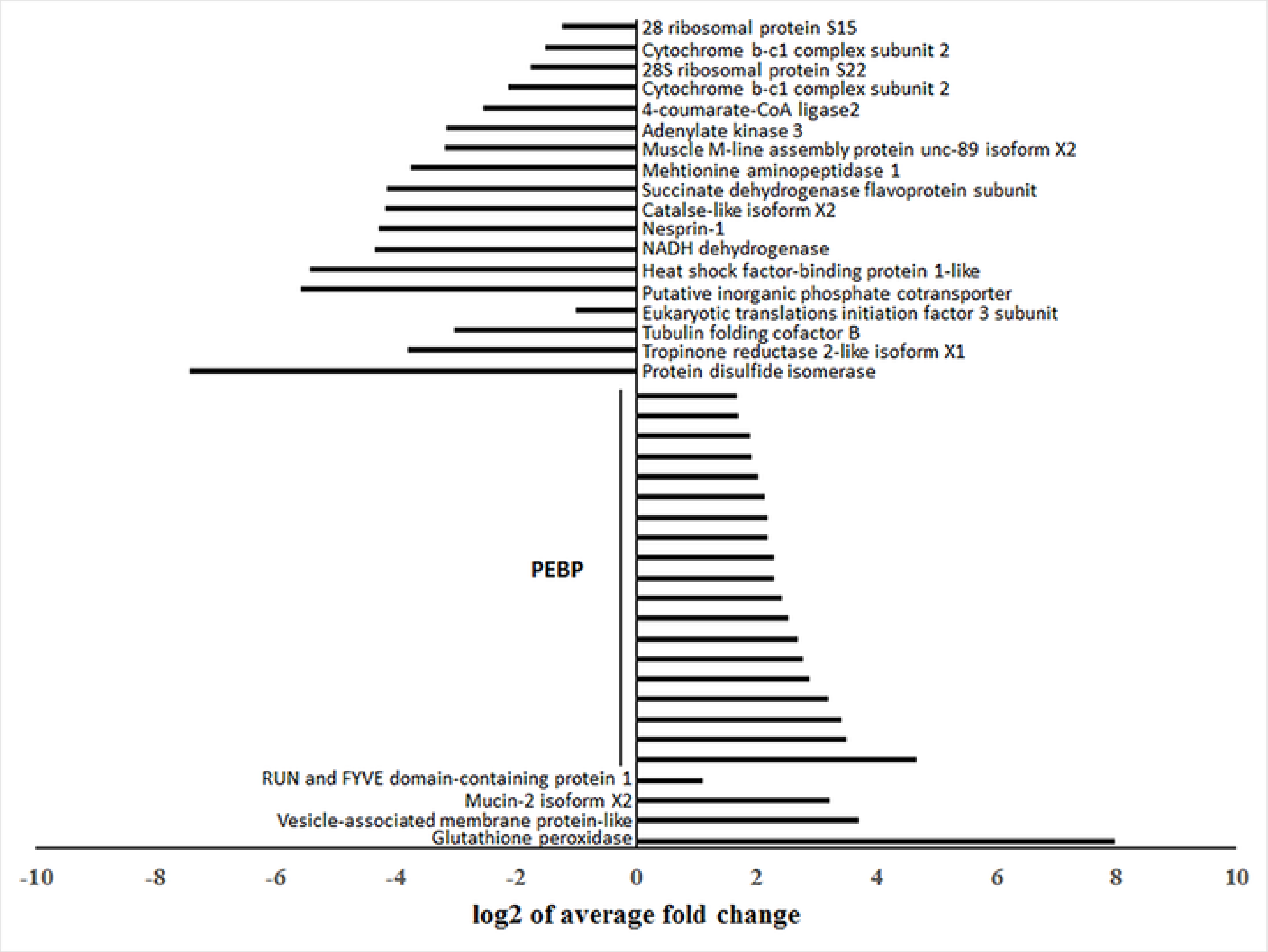

Figure 5

Click here to access/download;Figure;Figure 5.tif

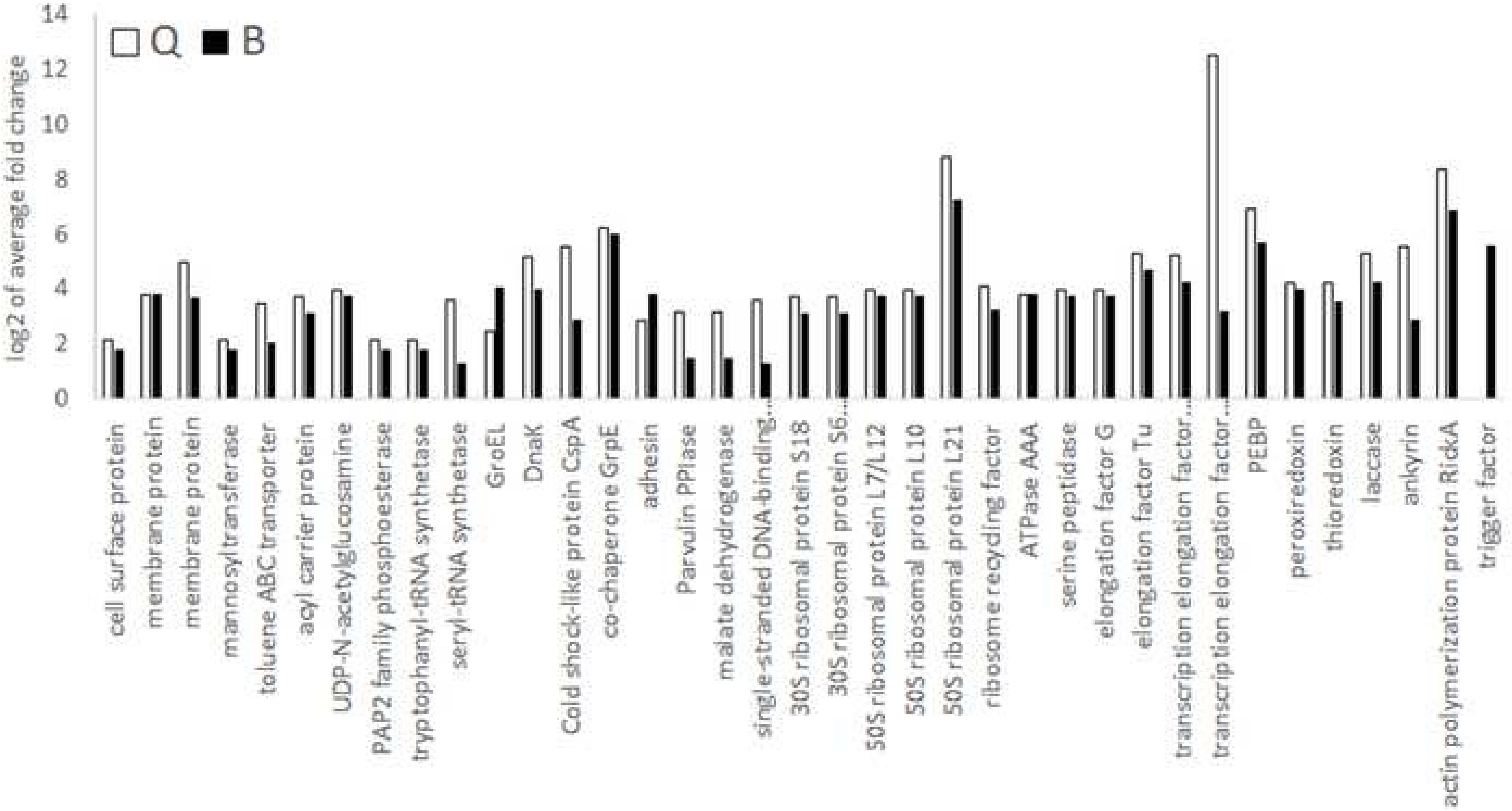

Figure 6

Click here to access/download;Figure;Figure 6.tif

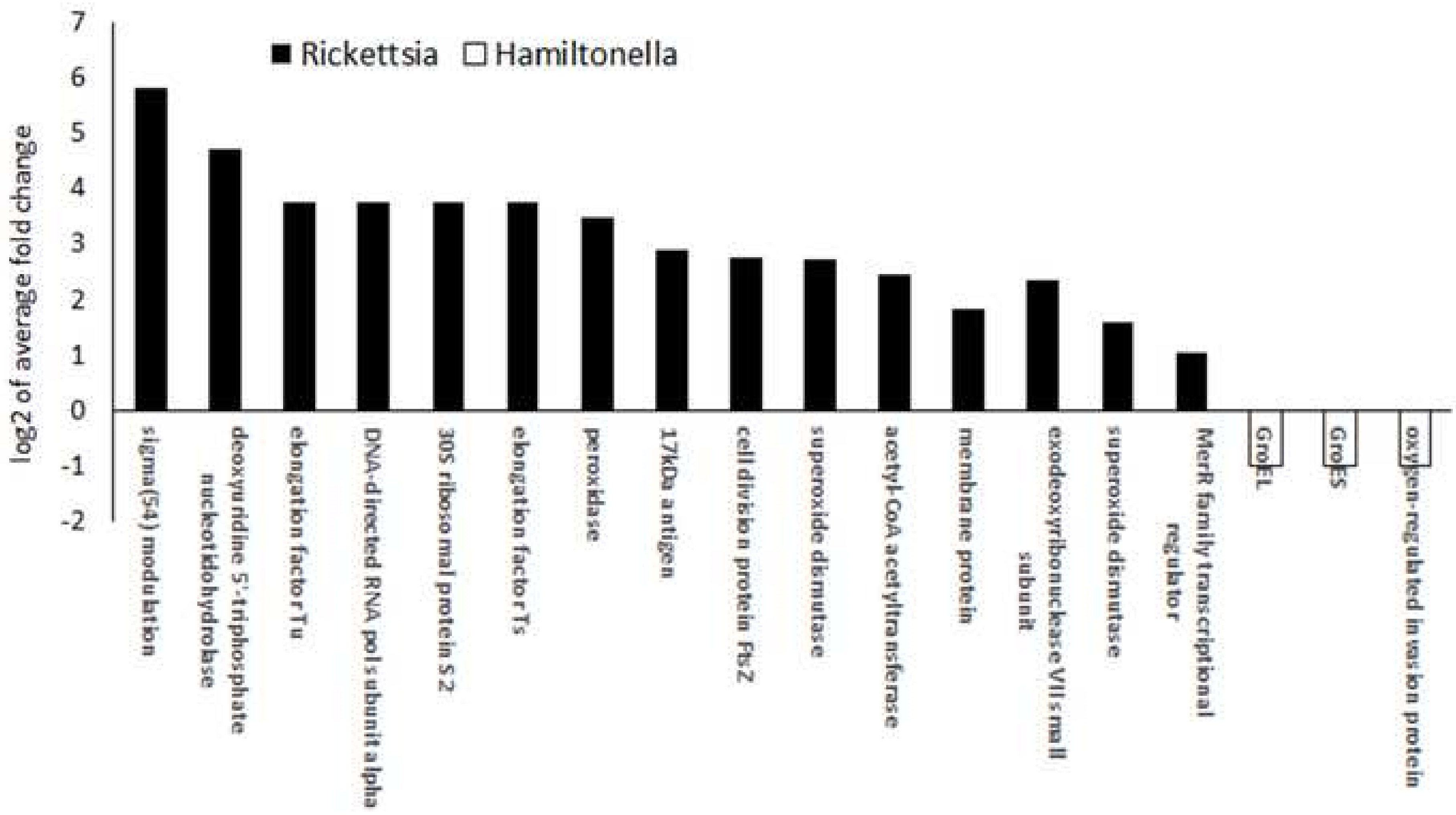

Supplementary figures

Click here to access/download
Supplementary Material
Supplementary figures.pdf

Cover letter

Click here to access/download;Personal Cover;cover letter.pdf

מדינת ישראל / משרד החקלאות ופיתוח הכפר
State of Israel / Ministry of Agriculture and Rural Development

Agricultural Research Organization
The Volcani Center
Institute of Plant Protection
Department of Entomology

Prof. Murad Ghanim

מינהל המחקר החקלאי
מרכז וולקני
המכון להגנת הצומח
המחלקה לאנטומולוגיה

פרופ' מוראד גאנם

1.4.2020

GigaScience Editorial,

Dear editors,

We are submitting a manuscript entitled "A proteomic approach reveals possible molecular mechanisms and roles for endosymbiotic bacteria in begomovirus transmission by whiteflies" to be considered for publication in GigaScience. The manuscript reports the first largest discovery proteomic study for the non-model insect pest, the whitefly *Bemisia tabaci.* This insect is known as one of the most invasive species worldwide and probably the most important vector of plant viruses, which transmits more than 100 plant viruses, some are devastating and cause direct and indirect losses estimated by billions of dollars annually.

Our manuscript used large-scale proteomic approaches to identify insect proteins associated with elevated virus transmission abilities, using very large datasets, thus we think it is suitable to be considered for publication in GigaScience. Additionally, GigaScience is the only journal that can house such a large proteomic dataset which spans a large experimental setup that includes two insect species and nine strains, each with three biological replicates and three Mass Spectrometry technical runs. The results presented and provided with this manuscript are also biologically relevant and addresses an important question in the vector biology field, and provides a list of potential insect and endosymbiotic bacteria candidate proteins that seem to have roles in the ability of good vector populations to transmit begomoviruses. Thus, the manuscript provides both a large proteomic dataset and biologically relevant results.

The authors of this manuscript declare no competing interests, and confirm that all all authors have approved the manuscript for submission, and that the content of the manuscript has not been published, or submitted for publication elsewhere.

We hope our manuscript will be found suitable for review and publication in GigaScience,

Sincerely,
Murad Ghanim

P.O. Box 6, Bet-Dagan 50250 ISRAEL – 50250 ת"ד 6, בית דגן
Tel/Fax +972-3-9683911 טל"/פקס:
WWW: http://www.agri.gov.il/en/people/759.aspx
