# Supplementary material for: A proteomic approach reveals possible molecular mechanisms and roles for endosymbiotic bacteria in begomovirus transmission by whiteflies
Source: Gigascience. 2020 Nov 13;9(11):giaa124. doi: 10.1093/gigascience/giaa124 (PMC7662926; doi:10.1093/gigascience/giaa124)
Supplement: giaa124_GIGA-D-20-00096_Revision_2 [file giaa124_giga-d-20-00096_revision_2.pdf]

GigaScience

A proteomic approach reveals possible molecular mechanisms and roles for endosymbiotic bacteria in begomovirus transmission by whiteflies

--Manuscript Draft--

**Manuscript Number:**	GIGA-D-20-00096R2
**Full Title:**	A proteomic approach reveals possible molecular mechanisms and roles for endosymbiotic bacteria in begomovirus transmission by whiteflies
**Article Type:**	Data Note
**Funding Information:**	
**Abstract:**	Background Many plant viruses are vector-borne and depend on arthropods for transmission between host plants. Begomoviruses, the largest, most damaging and emerging group of plant viruses, infect hundreds of plant species and new virus species of the group are discovered each year. Begomoviruses are transmitted by members of the whitefly Bemisia tabaci species complex in a persistent-circulative manner. Tomato yellow leaf curl virus (TYLCV) is one of the most devastating begomoviruses worldwide and causes major losses in tomato crops as well as in many agriculturally important plant species. Different B. tabaci populations vary in their virus transmission abilities; however, the causes for these variations are attributed among others to genetic differences among vector populations, as well as to differences in the bacterial symbionts housed within B. tabaci.<br>Results Here, we performed discovery proteomic analyses in nine whiteflies populations from both Middle East Asia Minor I (MEAM1 formerly known as B biotype) and Mediterranean (MED formerly known as Q biotype) species. We analysed our proteomic results based on the different TYLCV transmission abilities of the various populations included in the study. The results provide the first comprehensive list of candidate insect and bacterial symbiont (mainly Rickettsia) proteins associated with virus transmission.<br>Conclusions Our data demonstrate that the proteomic signature of better vectors populations, differ considerably when compared to less efficient vector ones in the two whitefly species tested in this study. While MEAM1 efficient vector populations has a more lenient immune system, the Q efficient vector populations has higher abundance of proteins possibly implicated in virus passage through cells. Both species show a strong link of the facultative symbiont Rickettsia to virus transmission.
**Corresponding Author:**	Murad Ghanim<br>Agricultural Research Organization Volcani Center<br>Rishon LeZion, ISRAEL
**Corresponding Author Secondary Information:**	
**Corresponding Author's Institution:**	Agricultural Research Organization Volcani Center
**Corresponding Author's Secondary Institution:**	
**First Author:**	Adi Kliot
**First Author Secondary Information:**	
**Order of Authors:**	Adi Kliot
	Richard Johnson
	Michael MacCoss
	Svetlana Kontsedalov
	Galina Lebedev
	Henryk Czosnek
	Michelle Heck

Powered by Editorial Manager® and ProduXion Manager® from Aries Systems Corporation

	Murad Ghanim
**Order of Authors Secondary Information:**	
**Response to Reviewers:**	We thank the reviewers and editor for their valuable and constructive comments. We have modified the manuscript based on the comments raised, converted the paper to a note as instructed. The list of comments that were addressed are given below (C) and our responses are given underneath (A):<br>Response to reviewer 1:<br>C: The result section under abstract- "A protein database, over-expressed and down downregulated proteins from efficient vector populations from two different B. tabaci species are provided."<br>There should be uniformity in the words used in the sentence, either up/down or over/under. The use of word "expressed" or "regulated" can't be used directly in proteomics study without supported by some biological results. As far as selection of words to explain identified proteins, I would suggest use Increased/ decreased abundant proteins.<br>A: We have changed all references to protein quantities in the manuscript to "higher / lower abundance".<br><br>C: "Data is available through ProteomeXchange with identifier PXD016964…" - I was not able to see the dataset as reviewer.<br>A: We apologise for issues with PRIDE. Those have now been fixed and the data is now available.<br><br>C: I suggest author should also provide increased/ decreased abundant proteins lists from nine whiteflies populations from both Middle East Asia Minor I (MEAM1 formerly known as B biotype) and Mediterranean (MED formerly known as Q biotype) species as supplementary files.<br><br>A: We have changed all references to protein quantities in the manuscript to "higher / lower abundance".<br><br>C: We then compared the expression levels of all peptides and proteins in order to identify proteins that are differentially expressed in each…"<br>Same as above, expression is not the correct term to use. You can write- We then compared the abundance levels of all peptides and proteins in order to identify proteins that are differentially abundant in each… Please change accordingly throughout in manuscript.<br><br>A: We have changed all references to protein quantities in the manuscript to "higher / lower abundance".<br><br>C: same as above. Please change to Proteins differentially abundant… and in subsequent places.<br><br>A: We have changed all references to protein quantities in the manuscript to "higher / lower abundance".<br><br>C: A spelling mistakes "specie"<br><br>A: mistake has been fixed.<br><br>C: The sentence "The over expression of these two proteins in the efficient-vector populations confirmed the validity of our approach regarding the MED efficient-vector population."<br>Author is referring two proteins, what are those proteins?<br><br>A: The sentence is referring to the low abundances of HSP70 and cyclophilin in MED. The reviewer is right to point out this sentence that was moved out of context in our discussion. The discussion has now been extensively edited and the above sentence has been deleted.

C: Protein were extracted as described in [58]- Wrong reference, it should be 57. Please check other references.
A: Reference was indeed wrong and has been updated.

C: “Pellets were saved and washed 3 times with cold acetone, dried and re-suspended in 8M urea in 100mM ABC.
Protein was quantified using a Bradford assay, protein integrity was examined by running 5µg from each sample on 1D gel …”
Author used Bradford assay of proteins dissolved in 8M urea in 100mM ABC. As it is known that Urea is incompatible with Bradford assay. So, I will suggest them to add gel pictures in manuscript.

A: Unfortunately, we could not get hold of all gel images in time for this deadline, however, we added figures S4 and S5 to the manuscript supplementary data in which we show the total ion current and base peak chromatograms for 3 samples; 2 technical replicates of the same biological replicate and one more biological replicate from the same population. These show high reproducibility among samples and good spread across the gradient.

C: please add final concentration or total amount of trypsin added to reaction mixture.

A: From the updated method section: “Samples were then diluted to ~1M urea with 100 mM ABC and Trypsin was added in a 1:50 ratio (Trypsin:protein).”

C: How was the elution schedule done? Was it a continuous gradient or stepwise?

A: From the updated method section: “Peptides were eluted off the column using a gradient of 2-35% acetonitrile in 0.1% formic acid over 120 minutes, followed by 35-60% acetonitrile over 10 minutes at a flow rate of 250 nl/min.”

C: Which was the data acquisition ion mode (positive?) set in the MS?

A: From the updated method section: “The resolution for MS was 60,000 at m/z 400 covering the m/z range of 400-2000. MS/MS spectra were acquired using a linear ion trap that provided unit resolution. The automatic gain control targets for MS in the orbitrap was 1e6, whereas for MS/MS it was 8000, and the maximum fill times were 20 and 80 msec, respectively. The MS/MS spectra were acquired using an isolation width of 2 m/z and a normalized collision energy (NCE) of 35.”

C: Which was the charge range of the selected peptides for MS?

A: From the updated method section: “The precursor ion threshold intensity was set to 5000 in order to trigger an MS/MS acquisition. Furthermore, MS/MS acquisitions were prevented for precursor charge states of 1, or if the charge state could not be discerned from the MS spectrum. Dynamic exclusion (including all isotope peaks) was set for 20 seconds. “

C: What is the model name of MS system?

A: From the updated method section: “All mass spectrometry was performed on a LTQ-Orbitrap-Velos (Thermo Fisher Scientific).”

C: How did author calculated abundance? please highlight.

A: From the updated method section: “Mascot files were then loaded into the Progenesis QI program (Nonlinear) and aligned to a randomly selected reference run.” “Average normalized abundance was calculated for each protein, based on protein features without conflict only, and fold change between the highest and lowest values was calculated. All peaks were then compared and only those showing a >2-fold in expression with P values <0.05 in MEAM1 and <0.01 in MED were selected for analysis.”

Powered by Editorial Manager® and ProduXion Manager® from Aries Systems Corporation

To clarify- per set of biological replicates, a random selected sample (selected based on run score calculated by Progenesis QI program, usually the run with the highest score on that set) was chosen as a reference run. Peak intensities for all proteins were calculated based on the reference run.

C: Describe about P H A W R C labelling in footnote.

A: From the updated table legend: “Symbiont Legend: P- Portiera, H- Hamiltonella, A- Arsenophonus, W- Wolbachia, R- Rickettsia, C- Cardinium.”

Response to reviewer 2:
C: The English written needs to be deeply improved.

A: the manuscript has undergone extensive editing and English proofing.

C: The discussion is no good. It seems only a description. The manuscript is too descriptive. Proteomics works are not like that!

A: We thank the reviewer for pointing this out and we agree
The discussion has been modified to be less ‘listy’, however many of the data we found important is still included. We tried editing and turning the discussion into a more ;story telling ‘ section drawing conclusions from the data in the results.

C: The authors have to make sense in all results they found out. In my opinion the discussion should be rewritten in a better way.

A: As written above, we rewrote and edited our discussion and hope it is now more fitting.

C: I suggest some additional experiments of qPCR to try to corroborates both RNA expression and protein data.

A: This manuscript is now submitted as a data note and the editor mentioned that there is no need for qPCR verification experiments in a data note. We also think that qPCRs will indicate the RNA levels and might not reflect the proteomic profile obtained in our results.

C: The conclusion is a merely short communication of the results. It has to be done again.

A: The conclusions have also been rewritten.

C: Where is the protein table? I need to all identified proteins.

A: We apologize for excluding this in the first submission. Supplementary table 1 is now added and includes all proteins that showed significantly different abundances in all 3 sets of biological replicates.

C: The MS runs is badly described! There are many question about MS analysis? How many runs were done for each sample? How many biological samples were used? Three? Five? How many peptides were used to identify a protein?

A: Three biological replicates were run per population and three technical runs for each biological replicate. We have rewritten the MS runs part of the methods and revised other parts of the methods’ section as well.
From the updated methods’ section: “Further sifting of the data was done to keep only

Powered by Editorial Manager® and ProduXion Manager® from Aries Systems Corporation

	proteins that had at least one unique peptide sequence identified.”
**Additional Information:**	
**Question**	**Response**
Are you submitting this manuscript to a special series or article collection?	No
**Experimental design and statistics**<br><br>Full details of the experimental design and statistical methods used should be given in the Methods section, as detailed in our Minimum Standards Reporting Checklist. Information essential to interpreting the data presented should be made available in the figure legends.<br><br>Have you included all the information requested in your manuscript?	Yes
**Resources**<br><br>A description of all resources used, including antibodies, cell lines, animals and software tools, with enough information to allow them to be uniquely identified, should be included in the Methods section. Authors are strongly encouraged to cite Research Resource Identifiers (RRIDs) for antibodies, model organisms and tools, where possible.<br><br>Have you included the information requested as detailed in our Minimum Standards Reporting Checklist?	Yes
**Availability of data and materials**<br><br>All datasets and code on which the conclusions of the paper rely must be either included in your submission or deposited in publicly available repositories (where available and ethically appropriate), referencing such data using a unique identifier in the references and in the “Availability of Data and Materials” section of your manuscript.	Yes

Have you have met the above requirement as detailed in our Minimum Standards Reporting Checklist?	

Manuscript with track changes

Click here to access/download;Manuscript;TYLCV proteomics track changes final.docx

A proteomic approach reveals possible molecular mechanisms and roles for endosymbiotic bacteria in begomovirus transmission by whiteflies

Adi Kliot,[a,b,c] Richard S Johnson,[d] Michael J MacCoss,[d] Svetlana Kontsedalov,[a] Galina Lebedev,[a] Henryk Czosnek,[b] Michelle Heck,[e] Murad Ghanim,[a*]

[a] Department of Entomology, The Volcani Center, Rishon LeZion, Israel

[b] Institute of Plant Sciences and Genetics in Agriculture, Robert H. Smith Faculty of Agriculture, Food and Environment, Hebrew University of Jerusalem, Rehovot, Israel

[c] Earlham Institute, Norwich, UK

[d] University of Washington

[e] USDA-Agricultural Research Service, Boyce Thompson Institute for Plant Research, Department of Plant Pathology and Plant-Microbe Biology, Cornell University, Ithaca, New York, USA

* Corresponding author

Email list:
Adi Kliot: adiaaa@gmail.com
Michael MacCoss: maccoss@uw.edu
Richard Johnson: rj8@uw.edu
Svetlana Kontsedalov: nasvetla@yahoo.com
Galina Lebedev: galinal@volcani.agri.gov.il
Henryk Czosnek: hanokh.czosnek@mail.huji.ac.il
Murad Ghanim: ghanim@volcani.agri.gov.il
Michelle Heck: mlc68@cornell.edu

Abstract

Background Many plant viruses are vector-borne and depend on arthropods for transmission between host plants. Begomoviruses, the largest, most damaging and emerging group of plant viruses, infect hundreds of plant species and new virus species of the group are discovered each year. Begomoviruses are transmitted by members of the whitefly *Bemisia tabaci* species complex in a persistent-circulative manner. *Tomato yellow leaf curl virus* (TYLCV) is one of the most devastating begomoviruses worldwide and causes major losses in tomato crops as well as in many agriculturally important plant species. Different *B. tabaci* populations vary in their virus transmission abilities; however, the causes for these variations are attributed among others to genetic differences among vector populations, as well as to differences in the bacterial symbionts housed within *B. tabaci.*

Results Here, we performed discovery proteomic analyses in nine whiteflies populations from both Middle East Asia Minor I (MEAM1 formerly known as B biotype) and Mediterranean (MED formerly known as Q biotype) species. We analysed our proteomic results based on the different TYLCV transmission abilities of the various populations included in the study. The results provide the first comprehensive list of candidate insect and bacterial symbiont (mainly *Rickettsia)* proteins associated with virus transmission.

Conclusions Our data demonstrate that the proteomic signature of better vectors populations, differ considerably when compared to less efficient vector ones in the two whitefly species tested in this study. While MEAM1 efficient vector populations has a more lenient immune system, the Q efficient vector populations has higher abundance of proteins possibly implicated in virus passage through cells. Both species show a strong link of the facultative symbiont *Rickettsia* to virus transmission.

Keywords

Bemisia tabaci, proteome, TYLC, transmission, bacterial symbiont

Data Description

The whitefly *Bemisia tabaci* is a serious threat to worldwide agriculture, yet an extensive analysis of its proteomic profile has not been performed before. The data we collected in this study represents the most extensive proteomic dataset available for this insect pest, or any hemipteran insect. We extracted total proteins for whole insects which were pooled from various populations and two different species, digested them to peptides and ran them on a mass spectrometer. Three biological replicates were collected per population and three technical replicates were run at random order per biological replicate. Data is available through ProteomeXchange with identifier PXD016964 and will be a valuable tool for future research of *B. tabaci* proteins involved in virus transmission and for further proteomic studies in insects.

Potential implications

The data provided here represent the first large scale discovery proteomics data set created for *Bemisia tabaci* MEAM1 and MED species, both worldwide pests of extremely economic importance. This data was used to mine different protein expression patterns correlated with virus transmission ability. The nine populations used in this study harbor different bacterial symbionts and have varying levels of resistance to insecticides. This dataset and the identified protein patterns provide basis to study other differences at the protein level. The dataset was searched against hundreds of thousands of available whitefly sequences in the public databases, however they were not searched against the published B and Q genomes since those exhibited tremendous differences at the assembly level and have yet to be well-

annotated. We thus preferred to compare the dataset we generated against available whitefly datasets, and with other insect species for which better genome sequences are available. In the future, the dataset provided here may be searched against the assembled genomes of both studied species.

Background

Since first described more than a 100 years ago, the whitefly *Bemisia tabaci* has become an agricultural pest distributed on a worldwide scale. Its importance stems from its extreme invasiveness with international commodity trade, rapidly occupying new niches and displacing local populations, and now considered one of the most invasive species worldwide. *B. tabaci* causes direct cosmetic damage to various crops during feeding, and by the attraction of sooty mold fungus to its sugar-rich honeydew secretions [1]. However, the ~~the~~ most serious damage caused by *B. tabaci* is virus transmission. *B. tabaci* is a vector for over 100 different plant viruses, primarily old and new world Begomoviruses of the family Geminiviridae. ~~However, the insect is expanding its~~ The whitefly's vectoring abilities aren't limited to Begomoviruses~~ty~~ and new viruses belonging to Potyviridae, Closteroviridae, Luteoviridae and Betaflexiviridae were also recently reported to be vectored by *B. tabaci* [2-4].

B. tabaci is a complex of morphologically indistinguishable species. Based on sequence polymorphism in defined mitochondrial genes, it is now agreed that *B. tabaci* comprises 11 species groups, each includes some species-complex members, previously termed as biotypes [5, 6]. The two most polyphagous and invasive species in this complex are the Middle East Asia Minor 1 (MEAM1 formerly known as the B biotype), and Mediterranean (MED formerly known as the Q biotype) [7]. Surveys conducted over the years in Israel have reported the presence of those two species only [8].

Recently, the genomes of both MEAM1 and MED have been sequenced and published [9, 10] creating a wealth of new resources for genetic and molecular studies. *B. tabaci* genomes, which are still being annotated, are highly divergent from that of previously sequenced hemipteran species and shows vast expansions in gene families related to metabolism and insecticide resistance [9].

Mass spectrometry based proteomic approaches have become a prevalent tool in research of various biological systems - from humans to arthropods. Recent studies performed on arthropods and entomopathogenic viruses were able to isolate and identify viral structural proteins and virions from both insect cell cultures and hemolymph [11, 12]. Proteomic studies, comparing efficient and non-efficient virus vector clone lines in aphids were able to identify protein markers linked to transmission ability: in the greenbug aphid, *Schizaphis graminum,* and *Cereal yellow dwarf virus*-RPV (CYDV-RPV) [13], and in the English grain aphid, *Sitobion avenae* and *Barley yellow dwarf virus*-PAV (BYDV-PAV) [14]. Proteomic studies conducted with *B. tabaci* thus far have focused on targeting proteins or genes for the development of new insecticides [15] or for studying insecticide resistance mechanisms [16].

In this manuscript we performed a discovery mass spectrometry analysis using nine populations from the MEAM1 and MED species collected in Israel and Croatia which vary in their *Tomato yellow leaf curl virus* (TYLCV) transmission ability. We compared the proteomic profiles between efficient TYLCV vector populations within each species and between the two species. We were able to identify previously undescribed proteins from *B. tabaci*, some of which are important for virus transmission. Such candidate proteins shed more light on the molecular mechanisms that underlay the insect-virus interactions during TYLCV transmission by *B. tabaci.*

Analyses

TYLCV Transmission assays

To characterize our selected populations with regard to their TYLCV transmission abilities we performed several transmission experiments. We identified a gradient of transmission abilities, with MEAM1 being in general a better vector for the virus compared to MED populations (figure 1 A and B). Our results are consistent with previously published results from Israel [17]. We identified MspRQ as the most efficient vector population of the MED species (figure 1 A) and ObeRB as the most efficient MEAM1 species TYLCV vector (figure 1 B).

Proteomic analysis

We used shotgun proteomics to compare the protein expression profile of the nine different populations of the two different *B. tabaci* species collected in Israel (figure 1). Data for each population composed of 3 biological replicates and 3 technical replicates per biological one. A PCA made of all data showed low percentage of variance originating from the biological replicates, proving high reproducibility of the technical and biological replicates (figure S1, supplementary data).

We were able to identify on average 3,350 proteins from 2,510 protein families with an average FDR of 0.9% in each replicate. We then compared the quantity of all peptides and proteins in order to identify proteins that differ in their abundance between TYLCV efficient vector populations compared to the other populations of the respective species. We found that the general level of variability was much higher between the different MED populations than between the MEAM1 populations. We limited our analysis to up to 15,000 peptides showing >2-fold change in abundance. In the MEAM1 population peptides, we used only peptides with P values <0.05; this approach produced too many results in MED, therefore we reduced

our analysis to peptides with P values of <0.01. This coincides with the findings showing that while MEAM1 and MED are derived from the same ancestral species, during speciation, MEAM1 remained stable while MED continued to separate into more species such as MED, J, L and others [5]. Therefore, while MEAM1 populations are more unified in their proteomic profiles, MED populations show higher variance.

Proteins differentially expressed in MEAM1 biotype efficient vector population

We compared each efficient vector population to other populations of the same ~~specie~~species and identified several interesting candidate proteins with possible functional roles in virus transmission (Figure 2). Out of 108 proteins that are significantly more abundant in the efficient biotype MEAM1 vector, the proteins with more than one peptide identified and with the highest expression levels were: a eukaryotic translation initiation factor 3, cathepsins B and F and a viral A inclusion protein (full list on table S1, supplementary data). Cathepsins are a large family of proteases, in arthropods they are primarily expressed in the digestive system. It is postulated that Cathepsin B proteases are excreted into the plant phloem or that they may assist in resistance to plant defensive secondary metabolites found in the plant sap [18].

We found 85 proteins with significantly lower abundance in the MEAM1 efficient-vector compared to all other MEAM1 populations (selected proteins shown in figure 2, full list on table S1, supplementary data). Of these proteins we found Chondroitin proteoglycan, HSP70, Hdd11 defense protein and two cuticular proteins analogous to peritrophin (CPAP). All of which were previously studied in relation to virus transmission or immune responses. All but Chondroitin proteoglycan, are known as virus transmission inhibitors; Hdd11 and CPAP are related to immune system and HSP70 was previously shown in whiteflies to inhibit TYLCV passage through the insect midgut epithelial cells [19-21].

Proteins differentially expressed in MED biotype efficient vector population

Among the 41 proteins significantly more abundant in the MED efficient-vector compared with all other MED populations, 20 were identified as PEBPs (figure 3, full list on table S1, supplementary data). Alignment of the DNA and amino acids sequences of those 20 candidates showed low sequence identity, implying that these peptides belong to different proteins of the same protein family.

Other prominent highly abundant proteins include a protein with a RUN and FYVE domain, a vesicle associated membrane protein, glutathione peroxidase and mucin-2 like protein. FYVE domain functions in membrane trafficking [22]. A FYVE containing phosphatidylinositol-3-phosphate in mammals was found to be a binding site initiating endocytosis and cell invasion of *Vesicular stomatitis virus*. Inhibition of the FYVE domain of the protein inhibited infection [23]. In arthropods, a FYVE domain containing a zinc-finger was found up regulated in *Litopenaeus vannamei* shrimp resistant to *Taura syndrome virus* [24].

A mucin-like protein was associated with the passage of *Plasmodium* through the guts of the mosquito *Aedes aegypti* [25]. It is also a possible target protein of Baculoviruses while crossing the plasma membrane of the arthropod host [26].

Proteins with lower abundance in the efficient TYLCV vector population included mitochondrial ribosomal and cytochrome b proteins, metabolism related enzymes such as methionine aminopeptidase 1 and adenylate kinase 3, a heat shock factor binding protein, tubulin folding protein and more (Figure 3, full list on table S1, supplementary data). Cytochrome b, was found to be down regulated in *Anopheles gambiae* midguts after acquisition of O'nyong-nyong Virus [27].

Bacterial proteins differentially expressed in TYLCV efficient-vector populations

Among the 41 common proteins highly abundant in the efficient vector populations from both species, 37 were bacterial proteins, all from the facultative endosymbiont *Rickettsia*. *Rickettsia* has been previously implicated in virus transmission. Although each species has a different secondary endosymbiont bacterial composition, *Rickettsia* proteins were the only ones found to have significantly different abundance in the efficient vector populations. In the MEAM1 efficient-vector population a total of 53 proteins were significantly up-regulated; 37 of which common with the MED efficient-vector population. In the MED population only one *Rickettsia* protein was not shared with MEAM1. The abundant *Rickettsia* proteins are adhesin and other membrane proteins and transporters, GroEL and chaperonins, transcription and elongation factors, ribosomal proteins, actin polymerization protein and trigger factor proteins. Fold change of those identified proteins was higher in MED for all proteins but GroEL and adhesin proteins, whose fold change were higher in MEAM1 populations (figure 4, full list on table S1, supplementary data).

In the MEAM1 efficient vector population, additional 15 *Rickettsia* proteins were significantly more abundant compared to the rest of the MEAM1 populations (Figure 5, full list on table S1, supplementary data). Six of them are transcription or DNA editing related, two are membrane related proteins, two ribosomal proteins and the rest are uncategorized. One of these proteins is ftsZ, which has a crucial role in the development of the central cytoskeletal septum during cell division, strengthening our hypothesis that *Rickettsia* is dividing and proliferating more in this efficient vector population [28]. Three *Hamiltonella* proteins were down-regulated in the MEAM1 efficient vector compared to the other populations (1.98-fold change). One of them is the *Hamiltonella* GroEL protein, previously mentioned in this section. It is very surprising that this protein, previously found to improve TYLCV transmission has a lower abundance in the efficient TYLCV vector. It was

hypothesized that the *Hamiltonella* GroEL aids TYLCV virions to avoid the insect immune system in the whitefly hemolymph [17]. Our results, indicating proliferation of *Rickettsia,* could imply that the immune system of efficient vector populations is a "lenient" one, therefore TYLCV virions need not bind to *Hamiltonella* GroEL in order to survive the passage through the hemolymph.

Discussion

Discovery of protein expression patterns in both efficient TYLCV vectors compared to the rest of the tested populations, resulted in six proteins that have significantly different abundances in both efficient vector populations (figure S3). However, all proteins with significantly different abundances that were common to both efficient vector populations of the two species showed different trends: Catalase, a Phosphatidylethanolamine binding protein (PEBP) and Cyclophilin were highly abundant in the MED efficient-vector while Vitellogenin and an antimicrobial protein Alo-2, were highly abundant in MEAM1 (figure S3). Several of these proteins were previously reported with regard to virus transmission; Cyclophilin, a peptydil prolyl-isomerase, was shown to be linked to CYDV-RPV transmission by the aphid *Schizaphis graminum*. Cyclophilin was found to be up-regulated in efficient-vector clone lines compared to inefficient-vector lines. It was also shown to bind to CYDV-RPV virions [29]. Different isoforms of the protein were shown to segregate between clones with different CYDV-RPV transmission efficiencies [30]. Three cyclophilin genes were identified in *B. tabaci* MEAM1 species: B, D and G. The expression of cyclophilin B was shown to be induced upon TYLCV infection, in the whitefly midgut [31]. TYLCV CP and cyclophilin were shown to co-localize in *B. tabaci* midguts and ovaries. Finally, feeding whiteflies with anti-cyclophilin antibodies, a cyclophilin inhibitor or cyclophilin dsRNA

greatly reduced TYLCV transmission rates [31, 32].

Alo-2 is a protein of the Knottin family, a highly diverse protein family with one common domain; the knottin fold. Knottin proteins are extensively studied in arthropods such as *Drosophila* and various Coleopterans with regards to the systemic immune response. Members of the Knottin family have been described to have antifungal and antibacterial functions [33, 34], while no antiviral response was yet identified. Alo-2 is likely to function in the immune response of *B. tabaci* and therefore its upregulation in the efficient vector is unexpected.

Vitellogenin, a large phospholipoglycoprotein involved in oogenesis and presumed to be a storage nutrient in the yolk. It is hypothesized to function as a hemaglutinating factor and an antibacterial effector in organisms from multiple kingdoms [35]. Wei et al. (2017) [36], demonstrated the crucial role of Vitellogenin in transovarial transmission of TYLCV in *B. tabaci* MEAM1 species, thus putting an end to a long-standing debate on the subject [36-39]. Wei et al. have shown that Vitellogenin binds to TYLCV coat protein and aids in the virus translocation into developing eggs inside the ovaries. Interestingly, this study showed that TYLCV was transovarially transmitted to eggs in mature females (11 days after emergence) significantly more efficiently than in young females (1 day after emergence). All samples collected for our study were 1-5 days after emergence, a life stage indicated to have lower TYLCV transovarial transmission efficiency, however we found elevated quantities of Vitellogenin in both MEAM1 and MED efficient vector populations. Interestingly, peptides spanning the entire vitellogenin sequence were found in high abundances in the MEAM1 efficient vector. In MED, peptides from a certain region of the protein were found to have low abundances, unlike the rest of the protein (supplementary figure S2). This might hint to the existence of different isoforms of vitellogenin in the two species.

Half of the significantly abundant protein in the MED efficient vector population were identified as PEBPs (figure 3). PEBPs were found to be linked to immune response activation against bacterial infection via the Toll immune pathway in *Drosophila melanogaster* [40, 41].

A PEBP was also found to be necessary for HIV1 infection [42]. The recent sequencing of the MEAM1 biotype genome showed that PEBP genes are more than 10-fold more abundant in the *B. tabaci* MEAM1 genome compared to 15 other arthropod genomes [9]. This finding, along with our data hints on the important role this gene family has in whiteflies, where they are likely participating in various processes, including virus transmission.

The significantly different abundances of all these proteins in the MED efficient vector population suggest that its midgut is more permeable and thus TYLCV circulation is more efficient. The gut barrier is known to be the first and often most important barrier for an insect-transmitted pathogen to cross along the transmission pathway especially in the whitefly-begomovirus interaction [43], and this barrier determines the efficiency and specificity of transmission.

In the MEAM1 efficient vector population, we identified 108 proteins with significantly higher abundance, of them Cathepsins B and F were highly represented. 78 proteins were of significantly lower abundance in the MEAM1 efficient vector population over the other MEAM1 populations. We identified several of them as immune-system proteins and known virus transmission inhibitors such as Chondroitin proteoglycan, HSP70, Hdd11 defense protein and two CPAPs. It is therefore expected that those proteins were down regulated in the efficient-vector, resulting in observed lower abundances, and demonstrating a more “less stringent" immune system in which TYLCV virions have higher chances of making a full passage through the whitefly tissues for ensuring successful transmission.

Among the 41 common proteins highly abundant in the efficient vector populations from both species, 37 were bacterial proteins encoded by *Rickettsia*. *Rickettsia* is the only shared secondary endosymbiont between MED and MEAM1 in Israel [8]. Six of the nine populations tested in this experiment were infected with *Rickettsia* (see table 1), however, no correlation was found between the presence of *Rickettsia* and TYLCV transmission efficiency. This could indicate that infection only is not enough to improve transmission ability; the expression of additional genes from the bacterium are needed. ~~The~~ Our data also does not suggest that there are higher titers of the bacterium in neither of the efficient vector populations.

Highly abundant *Rickettsia* proteins include adhesin and other membrane proteins and transporters, GroEL and chaperonins, transcription and elongation factors, ribosomal proteins, actin polymerization protein and trigger factor proteins. High quantities of proteins from all these groups indicate that the bacteria are propagating and undergoing cell division characteristic of a 'log phase' of bacterial growth in the better vector populations.

Our current study shows elevated levels of vitellogenin in efficient vector populations. We previously demonstrated that high levels of vitellogenin and high fecundity are associated with the presence of *Rickettsia* [44]. Taken together, these results point out to another possible effect of this bacterium on TYLCV transmission. The role of bacterial endosymbionts in plant virus transmission is still under debate [45]. We have previously demonstrated the significant effect of the secondary endosymbionts *Rickettsia* and *Hamiltonella* from *B. tabaci* on TYLCV transmission by this insect [17, 46, 47].

Conclusions

In this study we have a produced an extensive proteomic database for *B. tabaci*, a non-model insect, which could be very useful for studies related to understanding the biology and

ecology of this important insect pest and virus vector. We further demonstrated the possible uses of this database by comparing the proteomic profiles of different vector populations from two species and correlated the results with their TYLCV transmission efficiencies. Our results demonstrate that different molecular pathways in the insect may participate in the transmission of plant viruses, some might be crucial for the passage of the virus through insect organs. While in MEAM1 species we observed a decline of immune-related genes and virus transmission inhibitors, in MED we observed a wealth of possible target proteins that aid in TYLCV movement within and between cells. Most interestingly, we find that PEBPs, a recently-described and highly expanded protein family in whiteflies, have a strong link to TYLCV transmission in MED. The only shared group of proteins between both efficient vector populations of both species and highly abundant in both are proteins encoded by the endosymbiont *Rickettsia*. Utilizing the database we developed in this study~~, and the~~ we uncovered high number of proteins that~~, some~~ have a role in TYLCV- and possibly other B~~b~~egomoviruses- transmission. This~~,~~ is an important step for functional studies in this insect related to its biology and to virus transmission.

Formatted: No underline

Methods

Insect collections and rearing in the lab

B. tabaci populations were collected from various locations in Israel and Croatia (table 1) and reared on cotton seedlings (*Gossypium hirsutum* L. cv. Acala) in insect proof cages maintained in growth rooms under standard conditions of 25°C±2°C, 60% relative humidity, and a 14-h light/10-h dark photoperiod. Three to five biological replicates containing 200-500 individuals were collected from each population up to a week after adult emergence. Samples were ~~placed~~ stored at -80°C till samples from all populations were collected.

Virus transmission assays

In order to calculate TYLCV transmission efficiencies of whitefly populations, 6-7 days old adults from each population were given a 48-h acquisition access period (AAP) on a TYLCV-infected tomato plant. The insects were then used for a 7-day inoculation access period (IAP) on 4-week-old, non-infected tomato plants, one whitefly per plant- in leaf clip cages. Two weeks post inoculation, young leaves were collected from the plants for DNA extraction (using the Dellaporta protocol, detailed in [46]) and PCR for TYLCV detection (using primers listed in [46]). Three replicates of 30 plants each were performed for each population (except the Q-AWR population that was terminated after the first assay due to technical problems).

Protein extractions and preparations for MS analysis

Protein were extracted as described in [48]; samples were grinded using a mortar and pestle while kept frozen using liquid nitrogen. 1ml of 10% TCA-acetone, 2% β-mercaptoethanol was added per sample. Samples were then incubated for 16 h at -20°C, then centrifuged at 5000 x *g*, 4°C, 30 minutes. Pellets were saved and washed 3 times with cold acetone, dried and re-suspended in 8M urea in 100mM ABC.

Protein was quantified using a Bradford assay, protein integrity was examined by running 5μg from each sample on 1D gel with BSA as a control, and a Coomassie Brilliant Blue staining.

Three biological replicates were chosen per population. Protein samples then proceeded to reduction, Cystein blocking and Trypsin digestion- 50 μg of protein was added to a final volume of 10 mM of DTT in 100 mM ABC, samples were then incubated at 30°C for an hour. A final volume of 30 mM of MMTS in 100 mM ABC was added and samples were incubated for one hour in room temperature.

Samples were then diluted to ~1M urea with 100 mM ABC and Trypsin was added in a 1:50 ratio (Trypsin:protein). Samples were incubated for 16 h at 37°C, desalted using Waters Sep Pak SPE cartridges (according to manufacturer's protocol), dried and kept at -80°C till MS analyses.

MS runs

The dried tryptic digests were solubilized in 50 µl 0.2% trifluoracetic acid and 2% acetonitrile by vortexing for 10 minutes at 37ºC and bath sonication for 5 minutes. The solubilized digests were centrifuged at 10,000 g for 5 minutes in order to pellet any particulates that might cause HPLC clogging, and the supernatants were carefully removed and placed into autosampler vials. Injections of 3 µl resulted in approximately 2 µg total peptide loaded onto the column. The sample order was randomized and blocked by biological replicates. Every third injection was a random.

All mass spectrometry was performed on a LTQ-Orbitrap-Velos (Thermo Fisher Scientific). Samples were loaded onto a 150-µm Kasil fritted trap packed with Jupiter C12 90 Å material (Phenomenex) to a bed length of 2 cm at a flow rate of 2 µl/min. After loading and desalting using a total volume of 10 µl of 0.1% formic acid plus 2% acetonitrile, the trap was brought on-line with a pulled fused-silica capillary tip (75-µm i.d.) packed with 40 cm of Reprosil-Pur C18-AQ (3-µm bead diameter, Dr. Maisch) mounted in an in-house constructed microspray source and placed in line with a Waters Nanoacquity binary UPLC pump plus autosampler. Peptides were eluted off the column using a gradient of 2-35% acetonitrile in 0.1% formic acid over 120 minutes, followed by 35-60% acetonitrile over 10 minutes at a flow rate of 250 nl/min.

The mass spectrometer was operated using data dependent acquisition (DDA) where a maximum of fifteen MS/MS spectra were acquired per MS spectrum. The resolution for MS

Formatted: Indent: First line: 0", Don't add space between paragraphs of the same style, Line spacing: Double, Pattern: Clear

Formatted: Font: (Default) +Headings CS (Times New Roman), Font color: Auto, English (United States)

Formatted: Font: (Default) +Headings CS (Times New Roman), Font color: Auto, English (United States)

Formatted: Font: (Default) +Headings CS (Times New Roman), Font color: Auto, English (United States)

Formatted: Font: (Default) +Headings CS (Times New Roman), 12 pt, Font color: Auto, English (United States)

Formatted: Font: (Default) +Headings CS (Times New Roman), Font color: Auto, English (United States)

Formatted: Font: (Default) +Headings CS (Times New Roman), Font color: Auto, English (United States)

Formatted: Font: (Default) +Headings CS (Times New Roman), 12 pt, Font color: Auto, English (United States)

was 60,000 at m/z 400 covering the m/z range of 400-2000. MS/MS spectra were acquired using a linear ion trap that provided unit resolution. The automatic gain control targets for MS in the orbitrap was 1e6, whereas for MS/MS it was 8000, and the maximum fill times were 20 and 80 msec, respectively. The MS/MS spectra were acquired using an isolation width of 2 m/z and a normalized collision energy (NCE) of 35. The precursor ion threshold intensity was set to 5000 in order to trigger an MS/MS acquisition. Furthermore, MS/MS acquisitions were prevented for precursor charge states of 1, or if the charge state could not be discerned from the MS spectrum. Dynamic exclusion (including all isotope peaks) was set for 20 seconds.

Formatted: English (United States)

~~Dried protein pellets were sonicated for five minutes in 0.2% TFA 2% acetonitrile, strongly vortexed one minute, and vortexed further at 1,200 rpm at 37°C for at least 10 minutes. Samples given a hard spin to pellet particles prior to placing in auto-sampler vials, and 3 µl injected (i.e., 50 µg/ 75 µl x 3 µl loaded to trap). Jupiter C12 5-micron beads were used to make a 2 cm x 150 micron trap prior to a 30 cm x 75 micron Dr Maisch 3 micron C18 packed tip. Samples were analyzed on a ThermoScientific Orbitrap in the MacCoss lab (University of Washington) using a top 10 DDA method. Samples were analyzed in triplicate and randomized with a blank run every third injection.~~ Total Ion Current and Base Peak Chromatograms were analysed to insure that even amounts of protein extractions were injected from all samples and to study the reproducibility and the spread across the gradient of the technical and biological replicates (See figures S4 and S5, supplementary data). MS data were deposited to the ProteomeXchange consortium via PRIDE [49] with identifier PXD016964.

MS analysis and data annotations

An initial search of all animal protein sequences on NCBI (monthly) showed approximately

1,000 proteins identified per run. A FASTA database of whitefly and whitefly endosymbiont bacterial DNA sequences from NCBI was compiled and used for Mascot searching. Using this as a database, the search was drastically improved, with an average of 3350.5 peptides being matched per LCMS run, with an average FDR of 0.9%. Percolator [50, 51] was used for correcting for multiple hypothesis testing and computing q-values. Mascot files were then loaded into the Progenesis QI program (Nonlinear) and aligned to a randomly selected reference run. Each and every run was then aligned and problematic regions with low alignment were removed. The data was then analyzed 3 times, once for every technical replicate of every biological replicate. Hence the data was analyzed as three separate experiments, each containing three biological replicates for every population and one technical replicate of each. Average normalized abundance was calculated for each protein, based on protein features without conflict only, and fold change between the highest and lowest values was calculated. All peaks were then compared and only those showing a >2-fold in expression with P values <0.05 in MEAM1 and <0.01 in MED were selected for analysis. Further sifting of the data was done to keep only proteins that had at least one unique peptide sequence identified. All three final lists of proteins, from the three technical replicates, were then compared and only proteins that appeared in at least two of the three were kept (see table S1, supplementary data).

Funding

This work was funded by a Binational Agricultural Research and Development (BARD) travel grant to Adi Kliot.

Authors' contributions

AK- Investigation, Formal analysis, Validation, Visualization, Writing- original draft, Funding Acquisition

MM- Formal analysis

RJ- Formal analysis

GL- Resources

SK- Resources

HC- Supervision, Writing- review & editing

MH- Methodology, Resources, Validation, Data Curation, Funding Acquisition, Writing- review & editing

MG- Funding Acquisition, Conceptualization, Supervision, Writing- review & editing

~~MH- Methodology, Resources, Validation, Data Curation, Funding Acquisition, Writing- review & editing~~

References

1. Oliveira, M., T. Henneberry, and P. Anderson, *History, current status, and collaborative research projects for Bemisia tabaci.* Crop protection, 2001. **20**(9): p. 709-723.
2. Jones, D.R., *Plant viruses transmitted by whiteflies.* European Journal of Plant Pathology, 2003. **109**(3): p. 195-219.
3. Navas-Castillo, J., E. Fiallo-Olivé, and S. Sánchez-Campos, *Emerging virus diseases transmitted by whiteflies.* Annual Review of Phytopathology, 2011. **49**: p. 219-248.
4. Ghosh, S., et al., *Transmission of a new polerovirus infecting pepper by the whitefly Bemisia tabaci.* Journal of virology, 2019: p. JVI. 00488-19.
5. De Barro, P.J., et al., *Bemisia tabaci: a statement of species status.* Annual review of entomology, 2011. **56**: p. 1-19.
6. Liu, S.-s., J. Colvin, and P.J. De Barro, *Species Concepts as Applied to the Whitefly Bemisia tabaci Systematics: How Many Species Are There?* Journal of Integrative Agriculture, 2012. **11**(2): p. 176-186.
7. Brown, J., D. Frohlich, and R. Rosell, *The sweetpotato or silverleaf whiteflies: biotypes of Bemisia tabaci or a species complex?* Annual review of entomology, 1995. **40**(1): p. 511-534.

8. Chiel, E., et al., *Biotype-dependent secondary symbiont communities in sympatric populations of Bemisia tabaci.* Bulletin of Entomological Research, 2007. **97**(04): p. 407-413.
9. Chen, W., et al., *The draft genome of whitefly Bemisia tabaci MEAM1, a global crop pest, provides novel insights into virus transmission, host adaptation, and insecticide resistance.* BMC biology, 2016. **14**(1): p. 1-15.
10. Xie, W., et al., *Genome sequencing of the sweetpotato whitefly Bemisia tabaci MED/Q.* GigaScience, 2017. **6**(5): p. gix018.
11. Franco, C.F., et al., *Monitoring virus-like particle and viral protein production by intact cell MALDI-TOF mass spectrometry.* Talanta, 2010. **80**(4): p. 1561-1568.
12. Tsai, J.-M., et al., *Genomic and proteomic analysis of thirty-nine structural proteins of shrimp white spot syndrome virus.* Journal of virology, 2004. **78**(20): p. 11360-11370.
13. Cilia, M., et al., *Discovery and targeted LC-MS/MS of purified polerovirus reveals differences in the virus-host interactome associated with altered aphid transmission.* 2012.
14. Papura, D., et al., *Two-dimensional electrophoresis of proteins discriminates aphid clones of Sitobion avenae differing in BYDV-PAV transmission.* Archives of virology, 2002. **147**(10): p. 1881-1898.
15. Mishra, M., et al., *Proteome analysis of Bemisia tabaci suggests specific targets for RNAi mediated control.* Journal of proteomics, 2016. **132**: p. 93-102.
16. Yang, N., et al., *Transcriptomic and Proteomic Responses of Sweetpotato Whitefly, Bemisia tabaci, to Thiamethoxam.* PLoS ONE, 2013. **8**(5): p. e61820.
17. Gottlieb, Y., et al., *The transmission efficiency of tomato yellow leaf curl virus by the whitefly Bemisia tabaci is correlated with the presence of a specific symbiotic bacterium species.* Journal of virology, 2010. **84**(18): p. 9310-9317.
18. Rispe, C., et al., *Large gene family expansion and variable selective pressures for cathepsin B in aphids.* Molecular biology and evolution, 2008. **25**(1): p. 5-17.
19. Götz, M., et al., *Implication of Bemisia tabaci heat shock protein 70 in begomovirus-whitefly interactions.* Journal of virology, 2012. **86**(24): p. 13241-13252.
20. Bao, Y.-Y., et al., *De novo intestine-specific transcriptome of the brown planthopper Nilaparvata lugens revealed potential functions in digestion, detoxification and immune response.* Genomics, 2012. **99**(4): p. 256-264.
21. Wang, L., et al., *A new shrimp peritrophin-like gene from Exopalaemon carinicauda involved in white spot syndrome virus (WSSV) infection.* Fish & Shellfish Immunology, 2013. **35**(3): p. 840-846.
22. Leevers, S.J., B. Vanhaesebroeck, and M.D. Waterfield, *Signalling through phosphoinositide 3-kinases: the lipids take centre stage.* Current opinion in cell biology, 1999. **11**(2): p. 219-225.
23. Le Blanc, I., et al., *Endosome-to-cytosol transport of viral nucleocapsids.* Nature cell biology, 2005. **7**(7): p. 653-664.
24. Sookruksawong, S., et al., *RNA-Seq analysis reveals genes associated with resistance to Taura syndrome virus (TSV) in the Pacific white shrimp Litopenaeus vannamei.* Developmental & Comparative Immunology, 2013. **41**(4): p. 523-533.
25. Berois, M., J. Romero-Severson, and D. Severson, *RNAi knock-downs support roles for the mucin-like (AeIMUC1) gene and short-chain dehydrogenase/reductase (SDR) gene in Aedes aegypti susceptibility to Plasmodium gallinaceum.* Medical and veterinary entomology, 2012. **26**(1): p. 112-115.
26. Rohrmann, G.F., *The baculovirus replication cycle: Effects on cells and insects.* 2013.

27. Rider, M.A., et al., *Quantitative Proteomic Analysis of the Anopheles gambiae (Diptera: Culicidae) Midgut Infected With O'nyong–Nyong Virus.* Journal of medical entomology, 2013. **50**(5): p. 1077-1088.
28. Bramhill, D., *Bacterial cell division.* Annual review of cell and developmental biology, 1997. **13**(1): p. 395-424.
29. Yang, X., et al., *Coupling genetics and proteomics to identify aphid proteins associated with vector-specific transmission of polerovirus (Luteoviridae).* Journal of virology, 2008. **82**(1): p. 291-299.
30. Tamborindeguy, C., et al., *Genomic and proteomic analysis of Schizaphis graminum reveals cyclophilin proteins are involved in the transmission of Cereal yellow dwarf virus.* PloS one, 2013. **8**(8): p. e71620.
31. Kanakala, S. and M. Ghanim, *Implication of the whitefly Bemisia tabaci cyclophilin B protein in the transmission of Tomato yellow leaf curl virus.* Frontiers in plant science, 2016. **7**: p. 1702.
32. Kanakala, S., et al., *Plant-Mediated Silencing of the Whitefly Bemisia tabaci Cyclophilin B and Heat Shock Protein 70 impairs insect development and virus transmission.* Frontiers in physiology, 2019. **10**: p. 557.
33. Ntwasa, M., A. Goto, and S. Kurata, *Coleopteran antimicrobial peptides: prospects for clinical applications.* International journal of microbiology, 2012. **2012**.
34. Balmand, S., et al., *Antimicrobial peptides keep insect endosymbionts under control.* Science, 2011. **334**(6054): p. 362-365.
35. Zhang, S., et al., *Hemagglutinating and antibacterial activities of vitellogenin.* Fish & shellfish immunology, 2005. **19**(1): p. 93-95.
36. Wei, J., et al., *Vector development and vitellogenin determine the transovarial transmission of begomoviruses.* Proceedings of the National Academy of Sciences, 2017. **114**(26): p. 6746-6751.
37. Rubinstein, G. and H. Czosnek, *Long-term association of tomato yellow leaf curl virus with its whitefly vector Bemisia tabaci: effect on the insect transmission capacity, longevity and fecundity.* Journal of General Virology, 1997. **78**(10): p. 2683-2689.
38. Cohen, S. and F. Nitzany, *Transmission and host range of the tomato yellow leaf curl virus.* Phytopathology, 1966. **56**(10): p. 1127-1131.
39. Ghanim, M., et al., *Evidence for Transovarial Transmission of Tomato Yellow Leaf Curl Virus by Its Vector, the WhiteflyBemisia tabaci.* Virology, 1998. **240**(2): p. 295-303.
40. Levy, F., et al., *Peptidomic and proteomic analyses of the systemic immune response of Drosophila.* Biochimie, 2004. **86**(9): p. 607-616.
41. Reumer, A., et al., *Unraveling the protective effect of a Drosophila phosphatidylethanolamine-binding protein upon bacterial infection by means of proteomics.* Developmental & Comparative Immunology, 2009. **33**(11): p. 1186-1195.
42. Ott, D.E., et al., *Actin-binding cellular proteins inside human immunodeficiency virus type 1.* Virology, 2000. **266**(1): p. 42-51.
43. Pan, L., et al., *Differential efficiency of a begomovirus to cross the midgut of different species of whiteflies results in variation of virus transmission by the vectors.* Science China Life Sciences, 2018. **61**(10): p. 1254-1265.
44. Brumin, M., et al., *Levels of the endosymbiont Rickettsia in the whitefly Bemisia tabaci are influenced by the expression of vitellogenin.* Insect Molecular Biology, 2020. **29**(2): p. 241-255.

45. Pinheiro, P.V., et al., *Is there a role for symbiotic bacteria in plant virus transmission by insects?* Current Opinion in Insect Science, 2015. **8**: p. 69-78.
46. Kliot, A., et al., *Implication of the Bacterial Endosymbiont Rickettsia spp. in Interactions of the Whitefly Bemisia tabaci with Tomato yellow leaf curl virus.* Journal of virology, 2014. **88**(10): p. 5652-5660.
47. Kliot, A., et al., *Combined infection with Tomato yellow leaf curl virus and Rickettsia influences fecundity, attraction to infected plants and expression of immunity-related genes in the whitefly Bemisia tabaci.* Journal of General Virology, 2019. **100**(4): p. 721-731.
48. Cilia, M., et al., *A comparison of protein extraction methods suitable for gel-based proteomic studies of aphid proteins.* Journal of biomolecular techniques: JBT, 2009. **20**(4): p. 201.
49. Perez-Riverol, Y., et al., *The PRIDE database and related tools and resources in 2019: improving support for quantification data.* Nucleic acids research, 2019. **47**(D1): p. D442-D450.
50. Käll, L., et al., *Semi-supervised learning for peptide identification from shotgun proteomics datasets.* Nature methods, 2007. **4**(11): p. 923-925.
51. Spivak, M., et al., *Improvements to the percolator algorithm for peptide identification from shotgun proteomics data sets.* Journal of proteome research, 2009. **8**(7): p. 3737-3745.

Figure Legends

Figure 1. TYLCV Transmission abilities of MED (A) and MEAM1 (B) species populations used in this study. MspRQ and OberRB are the populations with the highest transmission efficiency in each species. Numbers above columns represent the number of plants tested for virus transmission with whiteflies from each population.

Figure 2. Top 40 differentially abundant proteins in OberRB. The 20 proteins with significantly low abundance and the 20 proteins with significantly high abundance in the MEAM1 efficient vector population compared to all other MEAM1 populations.

Figure 3. Top 40 differentially abundant proteins in MspRQ. The 20 proteins with significantly low abundance and the 20 proteins with significantly high abundance in the MED efficient vector population compared to the other MED populations.

Figure 4. *Rickettsia* proteins found at high quantities in both MEAM1 and MED efficient vector populations. Common bacterial proteins significantly abundant in MEAM1 (black) and MED (white) efficient vectors.

Figure 5. Additional symbiont (*Rickettsia* and *Hamiltonella)* proteins with high and low abundance in the MEAM1 efficient vector population.

Figure S1. Peptide PCAs for selected populations. Two PCA analyses for three randomly selected populations (of 9 in the experiment- three in A and three in B). Data for each PCA consisted of quantification of all peptides found in all three biological replicates performed for each population and all three technical replicates performed per biological replicate.

Figure S2. Proteins of differential expression common to both efficient vector populations show opposite abundances. Common proteins with significantly different quantities in MEAM1 (dark gray) and MED (light gray) efficient vectors.

Figure S3. *B. tabaci* complete Vitellogenin amino acids sequence. Highlighted are peptides identified to be of high abundance in the MEAM1 efficient TYLCV vector compared to the rest of the MEAM1 populations. Formatted are the peptides identified to be of low abundance in the MED efficient vector compared to the rest of MED populations.

Figure S4. Total Ion Current (TICs) of three selected runs. The TIC is the summed intensity of all ions (all m/zs) for the entire LCMS run. A and B are duplicate injections of the same sample and C is a biological replicate injection. TIC shows high reproducibility and a good spread across the gradient. The intensity of the second biological replicate (C), seems lower than the first biological replicate (A and B), which could be due to slightly lower concentration. As the data was normalized for comparison this isn't a problem.

Figure S5. Base Peak Chromatograms of three selected runs. base peak chromatograms for the same three runs as in figure S3. A and B are duplicate injections of the same sample and C is a biological replicate injection. The base peak chromatogram is the intensity of the most intense m/z peak during each scan. The base peaks are very reproducible between analytical replicates (A and B) and between biological replicates (A, B compared to C). The retention times are reproducible, many abundant peaks to within one minute.

Table 1. Populations collected and used in this study.

Symbiont Legend: P- *Portiera,* H- *Hamiltonella*, A- *Arsenophonus*, W- *Wolbachia*, R- *Rickettsia*, C- *Cardinium*.

	**Symbiont populations composition**						
**population name**	**P**	**H**	**A**	**W**	**R**	**C**	**collection site**
**MED populations**							
Q-AWR	+		+	+	+		Ayalon valley, Israel
fluf	+		+	+	+		Israel
Zadar	+	+	+	+			Zadar, Croatia
Q'-HC	+	+				+	Croatia
MspRQ	+		+	+	+		Israel
**MEAM1 populations**							
Ayalon	+	+			+		Ayalon Valley, Israel
MspRB	+	+					Israel
Tamra	+	+			+		Tamra, Israel
ObeRB	+	+			+		Israel

~~1.~~

Manuscript clean version

Click here to access/download;Manuscript;TYLCV proteomics clean final.docx

A proteomic approach reveals possible molecular mechanisms and roles for endosymbiotic bacteria in begomovirus transmission by whiteflies

Adi Kliot,[a,b,c] Richard S Johnson,[d] Michael J MacCoss,[d] Svetlana Kontsedalov,[a] Galina Lebedev,[a] Henryk Czosnek,[b] Michelle Heck,[e] Murad Ghanim,[a*]

[a] Department of Entomology, The Volcani Center, Rishon LeZion, Israel

[b] Institute of Plant Sciences and Genetics in Agriculture, Robert H. Smith Faculty of Agriculture, Food and Environment, Hebrew University of Jerusalem, Rehovot, Israel

[c] Earlham Institute, Norwich, UK

[d] University of Washington

[e] USDA-Agricultural Research Service, Boyce Thompson Institute for Plant Research, Department of Plant Pathology and Plant-Microbe Biology, Cornell University, Ithaca, New York, USA

* Corresponding author

Email list:
Adi Kliot: adiaaa@gmail.com
Michael MacCoss: maccoss@uw.edu
Richard Johnson: rj8@uw.edu
Svetlana Kontsedalov: nasvetla@yahoo.com
Galina Lebedev: galinal@volcani.agri.gov.il
Henryk Czosnek: hanokh.czosnek@mail.huji.ac.il
Murad Ghanim: ghanim@volcani.agri.gov.il
Michelle Heck: mlc68@cornell.edu

Abstract

Background Many plant viruses are vector-borne and depend on arthropods for transmission between host plants. Begomoviruses, the largest, most damaging and emerging group of plant viruses, infect hundreds of plant species and new virus species of the group are discovered each year. Begomoviruses are transmitted by members of the whitefly *Bemisia tabaci* species complex in a persistent-circulative manner. *Tomato yellow leaf curl virus* (TYLCV) is one of the most devastating begomoviruses worldwide and causes major losses in tomato crops as well as in many agriculturally important plant species. Different *B. tabaci* populations vary in their virus transmission abilities; however, the causes for these variations are attributed among others to genetic differences among vector populations, as well as to differences in the bacterial symbionts housed within *B. tabaci.*

Results Here, we performed discovery proteomic analyses in nine whiteflies populations from both Middle East Asia Minor I (MEAM1 formerly known as B biotype) and Mediterranean (MED formerly known as Q biotype) species. We analysed our proteomic results based on the different TYLCV transmission abilities of the various populations included in the study. The results provide the first comprehensive list of candidate insect and bacterial symbiont (mainly *Rickettsia)* proteins associated with virus transmission.

Conclusions Our data demonstrate that the proteomic signature of better vectors populations, differ considerably when compared to less efficient vector ones in the two whitefly species tested in this study. While MEAM1 efficient vector populations has a more lenient immune system, the Q efficient vector populations has higher abundance of proteins possibly implicated in virus passage through cells. Both species show a strong link of the facultative symbiont *Rickettsia* to virus transmission.

Keywords

Bemisia tabaci, proteome, TYLC, transmission, bacterial symbiont

Data Description

The whitefly *Bemisia tabaci* is a serious threat to worldwide agriculture, yet an extensive analysis of its proteomic profile has not been performed before. The data we collected in this study represents the most extensive proteomic dataset available for this insect pest, or any hemipteran insect. We extracted total proteins for whole insects which were pooled from various populations and two different species, digested them to peptides and ran them on a mass spectrometer. Three biological replicates were collected per population and three technical replicates were run at random order per biological replicate. Data is available through ProteomeXchange with identifier PXD016964 and will be a valuable tool for future research of *B. tabaci* proteins involved in virus transmission and for further proteomic studies in insects.

Potential implications

The data provided here represent the first large scale discovery proteomics data set created for *Bemisia tabaci* MEAM1 and MED species, both worldwide pests of extremely economic importance. This data was used to mine different protein expression patterns correlated with virus transmission ability. The nine populations used in this study harbor different bacterial symbionts and have varying levels of resistance to insecticides. This dataset and the identified protein patterns provide basis to study other differences at the protein level. The dataset was searched against hundreds of thousands of available whitefly sequences in the public databases, however they were not searched against the published B and Q genomes since those exhibited tremendous differences at the assembly level and have yet to be well-

annotated. We thus preferred to compare the dataset we generated against available whitefly datasets, and with other insect species for which better genome sequences are available. In the future, the dataset provided here may be searched against the assembled genomes of both studied species.

Background

Since first described more than a 100 years ago, the whitefly *Bemisia tabaci* has become an agricultural pest distributed on a worldwide scale. Its importance stems from its extreme invasiveness with international commodity trade, rapidly occupying new niches and displacing local populations, and now considered one of the most invasive species worldwide. *B. tabaci* causes direct cosmetic damage to various crops during feeding, and by the attraction of sooty mold fungus to its sugar-rich honeydew secretions [1]. However, the most serious damage caused by *B. tabaci* is virus transmission. *B. tabaci* is a vector for over 100 different plant viruses, primarily old and new world Begomoviruses of the family Geminiviridae. The whitefly's vectoring abilities aren't limited to Begomoviruses and new viruses belonging to Potyviridae, Closteroviridae, Luteoviridae and Betaflexiviridae were also recently reported to be vectored by *B. tabaci* [2-4].

B. tabaci is a complex of morphologically indistinguishable species. Based on sequence polymorphism in defined mitochondrial genes, it is now agreed that *B. tabaci* comprises 11 species groups, each includes some species-complex members, previously termed as biotypes [5, 6]. The two most polyphagous and invasive species in this complex are the Middle East Asia Minor 1 (MEAM1 formerly known as the B biotype), and Mediterranean (MED formerly known as the Q biotype) [7]. Surveys conducted over the years in Israel have reported the presence of those two species only [8].

Recently, the genomes of both MEAM1 and MED have been sequenced and published [9, 10] creating a wealth of new resources for genetic and molecular studies. *B. tabaci* genomes, which are still being annotated, are highly divergent from that of previously sequenced hemipteran species and shows vast expansions in gene families related to metabolism and insecticide resistance [9].

Mass spectrometry based proteomic approaches have become a prevalent tool in research of various biological systems - from humans to arthropods. Recent studies performed on arthropods and entomopathogenic viruses were able to isolate and identify viral structural proteins and virions from both insect cell cultures and hemolymph [11, 12]. Proteomic studies, comparing efficient and non-efficient virus vector clone lines in aphids were able to identify protein markers linked to transmission ability: in the greenbug aphid, *Schizaphis graminum,* and *Cereal yellow dwarf virus*-RPV (CYDV-RPV) [13], and in the English grain aphid, *Sitobion avenae* and *Barley yellow dwarf virus*-PAV (BYDV-PAV) [14]. Proteomic studies conducted with *B. tabaci* thus far have focused on targeting proteins or genes for the development of new insecticides [15] or for studying insecticide resistance mechanisms [16]. In this manuscript we performed a discovery mass spectrometry analysis using nine populations from the MEAM1 and MED species collected in Israel and Croatia which vary in their *Tomato yellow leaf curl virus* (TYLCV) transmission ability. We compared the proteomic profiles between efficient TYLCV vector populations within each species and between the two species. We were able to identify previously undescribed proteins from *B. tabaci*, some of which are important for virus transmission. Such candidate proteins shed more light on the molecular mechanisms that underlay the insect-virus interactions during TYLCV transmission by *B. tabaci.*

Analyses

TYLCV Transmission assays

To characterize our selected populations with regard to their TYLCV transmission abilities we performed several transmission experiments. We identified a gradient of transmission abilities, with MEAM1 being in general a better vector for the virus compared to MED populations (figure 1 A and B). Our results are consistent with previously published results from Israel [17]. We identified MspRQ as the most efficient vector population of the MED species (figure 1 A) and ObeRB as the most efficient MEAM1 species TYLCV vector (figure 1 B).

Proteomic analysis

We used shotgun proteomics to compare the protein expression profile of the nine different populations of the two different *B. tabaci* species collected in Israel (figure 1). Data for each population composed of 3 biological replicates and 3 technical replicates per biological one. A PCA made of all data showed low percentage of variance originating from the biological replicates, proving high reproducibility of the technical and biological replicates (figure S1, supplementary data).

We were able to identify on average 3,350 proteins from 2,510 protein families with an average FDR of 0.9% in each replicate. We then compared the quantity of all peptides and proteins in order to identify proteins that differ in their abundance between TYLCV efficient vector populations compared to the other populations of the respective species. We found that the general level of variability was much higher between the different MED populations than between the MEAM1 populations. We limited our analysis to up to 15,000 peptides showing >2-fold change in abundance. In the MEAM1 population peptides, we used only peptides with P values <0.05; this approach produced too many results in MED, therefore we reduced

our analysis to peptides with P values of <0.01. This coincides with the findings showing that while MEAM1 and MED are derived from the same ancestral species, during speciation, MEAM1 remained stable while MED continued to separate into more species such as MED, J, L and others [5]. Therefore, while MEAM1 populations are more unified in their proteomic profiles, MED populations show higher variance.

Proteins differentially expressed in MEAM1 biotype efficient vector population

We compared each efficient vector population to other populations of the same species and identified several interesting candidate proteins with possible functional roles in virus transmission (Figure 2). Out of 108 proteins that are significantly more abundant in the efficient biotype MEAM1 vector, the proteins with more than one peptide identified and with the highest expression levels were: a eukaryotic translation initiation factor 3, cathepsins B and F and a viral A inclusion protein (full list on table S1, supplementary data). Cathepsins are a large family of proteases, in arthropods they are primarily expressed in the digestive system. It is postulated that Cathepsin B proteases are excreted into the plant phloem or that they may assist in resistance to plant defensive secondary metabolites found in the plant sap [18].

We found 85 proteins with significantly lower abundance in the MEAM1 efficient-vector compared to all other MEAM1 populations (selected proteins shown in figure 2, full list on table S1, supplementary data). Of these proteins we found Chondroitin proteoglycan, HSP70, Hdd11 defense protein and two cuticular proteins analogous to peritrophin (CPAP). All of which were previously studied in relation to virus transmission or immune responses. All but Chondroitin proteoglycan, are known as virus transmission inhibitors; Hdd11 and CPAP are related to immune system and HSP70 was previously shown in whiteflies to inhibit TYLCV passage through the insect midgut epithelial cells [19-21].

Proteins differentially expressed in MED biotype efficient vector population

Among the 41 proteins significantly more abundant in the MED efficient-vector compared with all other MED populations, 20 were identified as PEBPs (figure 3, full list on table S1, supplementary data). Alignment of the DNA and amino acids sequences of those 20 candidates showed low sequence identity, implying that these peptides belong to different proteins of the same protein family.

Other prominent highly abundant proteins include a protein with a RUN and FYVE domain, a vesicle associated membrane protein, glutathione peroxidase and mucin-2 like protein. FYVE domain functions in membrane trafficking [22]. A FYVE containing phosphatidylinositol-3-phosphate in mammals was found to be a binding site initiating endocytosis and cell invasion of *Vesicular stomatitis virus*. Inhibition of the FYVE domain of the protein inhibited infection [23]. In arthropods, a FYVE domain containing a zinc-finger was found up regulated in *Litopenaeus vannamei* shrimp resistant to *Taura syndrome virus* [24].

A mucin-like protein was associated with the passage of *Plasmodium* through the guts of the mosquito *Aedes aegypti* [25]. It is also a possible target protein of Baculoviruses while crossing the plasma membrane of the arthropod host [26].

Proteins with lower abundance in the efficient TYLCV vector population included mitochondrial ribosomal and cytochrome b proteins, metabolism related enzymes such as methionine aminopeptidase 1 and adenylate kinase 3, a heat shock factor binding protein, tubulin folding protein and more (Figure 3, full list on table S1, supplementary data). Cytochrome b, was found to be down regulated in *Anopheles gambiae* midguts after acquisition of O’nyong-nyong Virus [27].

Bacterial proteins differentially expressed in TYLCV efficient-vector populations

Among the 41 common proteins highly abundant in the efficient vector populations from both species, 37 were bacterial proteins, all from the facultative endosymbiont *Rickettsia*. *Rickettsia* has been previously implicated in virus transmission. Although each species has a different secondary endosymbiont bacterial composition, *Rickettsia* proteins were the only ones found to have significantly different abundance in the efficient vector populations. In the MEAM1 efficient-vector population a total of 53 proteins were significantly up-regulated; 37 of which common with the MED efficient-vector population. In the MED population only one *Rickettsia* protein was not shared with MEAM1. The abundant *Rickettsia* proteins are adhesin and other membrane proteins and transporters, GroEL and chaperonins, transcription and elongation factors, ribosomal proteins, actin polymerization protein and trigger factor proteins. Fold change of those identified proteins was higher in MED for all proteins but GroEL and adhesin proteins, whose fold change were higher in MEAM1 populations (figure 4, full list on table S1, supplementary data).

In the MEAM1 efficient vector population, additional 15 *Rickettsia* proteins were significantly more abundant compared to the rest of the MEAM1 populations (Figure 5, full list on table S1, supplementary data). Six of them are transcription or DNA editing related, two are membrane related proteins, two ribosomal proteins and the rest are uncategorized. One of these proteins is ftsZ, which has a crucial role in the development of the central cytoskeletal septum during cell division, strengthening our hypothesis that *Rickettsia* is dividing and proliferating more in this efficient vector population [28]. Three *Hamiltonella* proteins were down-regulated in the MEAM1 efficient vector compared to the other populations (1.98-fold change). One of them is the *Hamiltonella* GroEL protein, previously mentioned in this section. It is very surprising that this protein, previously found to improve TYLCV transmission has a lower abundance in the efficient TYLCV vector. It was

hypothesized that the *Hamiltonella* GroEL aids TYLCV virions to avoid the insect immune system in the whitefly hemolymph [17]. Our results, indicating proliferation of *Rickettsia,* could imply that the immune system of efficient vector populations is a "lenient" one, therefore TYLCV virions need not bind to *Hamiltonella* GroEL in order to survive the passage through the hemolymph.

Discussion

Discovery of protein expression patterns in both efficient TYLCV vectors compared to the rest of the tested populations, resulted in six proteins that have significantly different abundances in both efficient vector populations (figure S3). However, all proteins with significantly different abundances that were common to both efficient vector populations of the two species showed different trends: Catalase, a Phosphatidylethanolamine binding protein (PEBP) and Cyclophilin were highly abundant in the MED efficient-vector while Vitellogenin and an antimicrobial protein Alo-2, were highly abundant in MEAM1 (figure S3). Several of these proteins were previously reported with regard to virus transmission; Cyclophilin, a peptydil prolyl-isomerase, was shown to be linked to CYDV-RPV transmission by the aphid *Schizaphis graminum*. Cyclophilin was found to be up-regulated in efficient-vector clone lines compared to inefficient-vector lines. It was also shown to bind to CYDV-RPV virions [29]. Different isoforms of the protein were shown to segregate between clones with different CYDV-RPV transmission efficiencies [30]. Three cyclophilin genes were identified in *B. tabaci* MEAM1 species: B, D and G. The expression of cyclophilin B was shown to be induced upon TYLCV infection, in the whitefly midgut [31]. TYLCV CP and cyclophilin were shown to co-localize in *B. tabaci* midguts and ovaries. Finally, feeding whiteflies with anti-cyclophilin antibodies, a cyclophilin inhibitor or cyclophilin dsRNA

greatly reduced TYLCV transmission rates [31, 32].

Alo-2 is a protein of the Knottin family, a highly diverse protein family with one common domain; the knottin fold. Knottin proteins are extensively studied in arthropods such as *Drosophila* and various Coleopterans with regards to the systemic immune response. Members of the Knottin family have been described to have antifungal and antibacterial functions [33, 34], while no antiviral response was yet identified. Alo-2 is likely to function in the immune response of *B. tabaci* and therefore its upregulation in the efficient vector is unexpected.

Vitellogenin, a large phospholipoglycoprotein involved in oogenesis and presumed to be a storage nutrient in the yolk. It is hypothesized to function as a hemaglutinating factor and an antibacterial effector in organisms from multiple kingdoms [35]. Wei et al. (2017) [36], demonstrated the crucial role of Vitellogenin in transovarial transmission of TYLCV in *B. tabaci* MEAM1 species, thus putting an end to a long-standing debate on the subject [36-39]. Wei et al. have shown that Vitellogenin binds to TYLCV coat protein and aids in the virus translocation into developing eggs inside the ovaries. Interestingly, this study showed that TYLCV was transovarially transmitted to eggs in mature females (11 days after emergence) significantly more efficiently than in young females (1 day after emergence). All samples collected for our study were 1-5 days after emergence, a life stage indicated to have lower TYLCV transovarial transmission efficiency, however we found elevated quantities of Vitellogenin in both MEAM1 and MED efficient vector populations. Interestingly, peptides spanning the entire vitellogenin sequence were found in high abundances in the MEAM1 efficient vector. In MED, peptides from a certain region of the protein were found to have low abundances, unlike the rest of the protein (supplementary figure S2). This might hint to the existence of different isoforms of vitellogenin in the two species.

Half of the significantly abundant protein in the MED efficient vector population were identified as PEBPs (figure 3). PEBPs were found to be linked to immune response activation against bacterial infection via the Toll immune pathway in *Drosophila melanogaster* [40, 41].

A PEBP was also found to be necessary for HIV1 infection [42]. The recent sequencing of the MEAM1 biotype genome showed that PEBP genes are more than 10-fold more abundant in the *B. tabaci* MEAM1 genome compared to 15 other arthropod genomes [9]. This finding, along with our data hints on the important role this gene family has in whiteflies, where they are likely participating in various processes, including virus transmission.

The significantly different abundances of all these proteins in the MED efficient vector population suggest that its midgut is more permeable and thus TYLCV circulation is more efficient. The gut barrier is known to be the first and often most important barrier for an insect-transmitted pathogen to cross along the transmission pathway especially in the whitefly-begomovirus interaction [43], and this barrier determines the efficiency and specificity of transmission.

In the MEAM1 efficient vector population, we identified 108 proteins with significantly higher abundance, of them Cathepsins B and F were highly represented. 78 proteins were of significantly lower abundance in the MEAM1 efficient vector population over the other MEAM1 populations. We identified several of them as immune-system proteins and known virus transmission inhibitors such as Chondroitin proteoglycan, HSP70, Hdd11 defense protein and two CPAPs. It is therefore expected that those proteins were down regulated in the efficient-vector, resulting in observed lower abundances, and demonstrating a more “less stringent" immune system in which TYLCV virions have higher chances of making a full passage through the whitefly tissues for ensuring successful transmission.

Among the 41 common proteins highly abundant in the efficient vector populations from both species, 37 were bacterial proteins encoded by *Rickettsia*. *Rickettsia* is the only shared secondary endosymbiont between MED and MEAM1 in Israel [8]. Six of the nine populations tested in this experiment were infected with *Rickettsia* (see table 1), however, no correlation was found between the presence of *Rickettsia* and TYLCV transmission efficiency. This could indicate that infection only is not enough to improve transmission ability; the expression of additional genes from the bacterium are needed. Our data also does not suggest that there are higher titers of the bacterium in neither of the efficient vector populations.

Highly abundant *Rickettsia* proteins include adhesin and other membrane proteins and transporters, GroEL and chaperonins, transcription and elongation factors, ribosomal proteins, actin polymerization protein and trigger factor proteins. High quantities of proteins from all these groups indicate that the bacteria are propagating and undergoing cell division characteristic of a 'log phase' of bacterial growth in the better vector populations.

Our current study shows elevated levels of vitellogenin in efficient vector populations. We previously demonstrated that high levels of vitellogenin and high fecundity are associated with the presence of *Rickettsia* [44]. Taken together, these results point out to another possible effect of this bacterium on TYLCV transmission. The role of bacterial endosymbionts in plant virus transmission is still under debate [45]. We have previously demonstrated the significant effect of the secondary endosymbionts *Rickettsia* and *Hamiltonella* from *B. tabaci* on TYLCV transmission by this insect [17, 46, 47].

Conclusions

In this study we have a produced an extensive proteomic database for *B. tabaci,* a non-model insect, which could be very useful for studies related to understanding the biology and

ecology of this important insect pest and virus vector. We further demonstrated the possible uses of this database by comparing the proteomic profiles of different vector populations from two species and correlated the results with their TYLCV transmission efficiencies. Our results demonstrate that different molecular pathways in the insect may participate in the transmission of plant viruses, some might be crucial for the passage of the virus through insect organs. While in MEAM1 species we observed a decline of immune-related genes and virus transmission inhibitors, in MED we observed a wealth of possible target proteins that aid in TYLCV movement within and between cells. Most interestingly, we find that PEBPs, a recently-described and highly expanded protein family in whiteflies, have a strong link to TYLCV transmission in MED. The only shared group of proteins between both efficient vector populations of both species and highly abundant in both are proteins encoded by the endosymbiont *Rickettsia*. Utilizing the database we developed in this study we uncovered high number of proteins that have a role in TYLCV- and possibly other Begomoviruses- transmission. This is an important step for functional studies in this insect related to its biology and to virus transmission.

Methods

Insect collections and rearing in the lab

B. tabaci populations were collected from various locations in Israel and Croatia (table 1) and reared on cotton seedlings (*Gossypium hirsutum* L. cv. Acala) in insect proof cages maintained in growth rooms under standard conditions of 25°C±2°C, 60% relative humidity, and a 14-h light/10-h dark photoperiod. Three to five biological replicates containing 200-500 individuals were collected from each population up to a week after adult emergence. Samples were stored at -80°C till samples from all populations were collected.

Virus transmission assays

In order to calculate TYLCV transmission efficiencies of whitefly populations, 6-7 days old adults from each population were given a 48-h acquisition access period (AAP) on a TYLCV-infected tomato plant. The insects were then used for a 7-day inoculation access period (IAP) on 4-week-old, non-infected tomato plants, one whitefly per plant- in leaf clip cages. Two weeks post inoculation, young leaves were collected from the plants for DNA extraction (using the Dellaporta protocol, detailed in [46]) and PCR for TYLCV detection (using primers listed in [46]). Three replicates of 30 plants each were performed for each population (except the Q-AWR population that was terminated after the first assay due to technical problems).

Protein extractions and preparations for MS analysis

Protein were extracted as described in [48]; samples were grinded using a mortar and pestle while kept frozen using liquid nitrogen. 1ml of 10% TCA-acetone, 2% β-mercaptoethanol was added per sample. Samples were then incubated for 16 h at -20°C, then centrifuged at 5000 x *g*, 4°C, 30 minutes. Pellets were saved and washed 3 times with cold acetone, dried and re-suspended in 8M urea in 100mM ABC.

Protein was quantified using a Bradford assay, protein integrity was examined by running 5µg from each sample on 1D gel with BSA as a control, and a Coomassie Brilliant Blue staining.

Three biological replicates were chosen per population. Protein samples then proceeded to reduction, Cystein blocking and Trypsin digestion- 50 µg of protein was added to a final volume of 10 mM of DTT in 100 mM ABC, samples were then incubated at 30°C for an hour. A final volume of 30 mM of MMTS in 100 mM ABC was added and samples were incubated for one hour in room temperature.

Samples were then diluted to ~1M urea with 100 mM ABC and Trypsin was added in a 1:50 ratio (Trypsin:protein). Samples were incubated for 16 h at 37°C, desalted using Waters Sep Pak SPE cartridges (according to manufacturer's protocol), dried and kept at -80°C till MS analyses.

MS runs

The dried tryptic digests were solubilized in 50 µl 0.2% trifluoracetic acid and 2% acetonitrile by vortexing for 10 minutes at 37ºC and bath sonication for 5 minutes. The solubilized digests were centrifuged at 10,000 g for 5 minutes in order to pellet any particulates that might cause HPLC clogging, and the supernatants were carefully removed and placed into autosampler vials. Injections of 3 µl resulted in approximately 2 µg total peptide loaded onto the column. The sample order was randomized and blocked by biological replicates. Every third injection was a random.

All mass spectrometry was performed on a LTQ-Orbitrap-Velos (Thermo Fisher Scientific). Samples were loaded onto a 150-µm Kasil fritted trap packed with Jupiter C12 90 Å material (Phenomenex) to a bed length of 2 cm at a flow rate of 2 µl/min. After loading and desalting using a total volume of 10 µl of 0.1% formic acid plus 2% acetonitrile, the trap was brought on-line with a pulled fused-silica capillary tip (75-µm i.d.) packed with 40 cm of Reprosil-Pur C18-AQ (3-µm bead diameter, Dr. Maisch) mounted in an in-house constructed microspray source and placed in line with a Waters Nanoacquity binary UPLC pump plus autosampler. Peptides were eluted off the column using a gradient of 2-35% acetonitrile in 0.1% formic acid over 120 minutes, followed by 35-60% acetonitrile over 10 minutes at a flow rate of 250 nl/min.

The mass spectrometer was operated using data dependent acquisition (DDA) where a maximum of fifteen MS/MS spectra were acquired per MS spectrum. The resolution for MS

was 60,000 at m/z 400 covering the m/z range of 400-2000. MS/MS spectra were acquired using a linear ion trap that provided unit resolution. The automatic gain control targets for MS in the orbitrap was 1e6, whereas for MS/MS it was 8000, and the maximum fill times were 20 and 80 msec, respectively. The MS/MS spectra were acquired using an isolation width of 2 m/z and a normalized collision energy (NCE) of 35. The precursor ion threshold intensity was set to 5000 in order to trigger an MS/MS acquisition. Furthermore, MS/MS acquisitions were prevented for precursor charge states of 1, or if the charge state could not be discerned from the MS spectrum. Dynamic exclusion (including all isotope peaks) was set for 20 seconds.

Total Ion Current and Base Peak Chromatograms were analysed to insure that even amounts of protein extractions were injected from all samples and to study the reproducibility and the spread across the gradient of the technical and biological replicates (See figures S4 and S5, supplementary data). MS data were deposited to the ProteomeXchange consortium via PRIDE [49] with identifier PXD016964.

MS analysis and data annotations

An initial search of all animal protein sequences on NCBI (monthly) showed approximately 1,000 proteins identified per run. A FASTA database of whitefly and whitefly endosymbiont bacterial DNA sequences from NCBI was compiled and used for Mascot searching. Using this as a database, the search was drastically improved, with an average of 3350.5 peptides being matched per LCMS run, with an average FDR of 0.9%. Percolator [50, 51] was used for correcting for multiple hypothesis testing and computing q-values. Mascot files were then loaded into the Progenesis QI program (Nonlinear) and aligned to a randomly selected reference run. Each and every run was then aligned and problematic regions with low alignment were removed. The data was then analyzed 3 times, once for every technical

replicate of every biological replicate. Hence the data was analyzed as three separate experiments, each containing three biological replicates for every population and one technical replicate of each. Average normalized abundance was calculated for each protein, based on protein features without conflict only, and fold change between the highest and lowest values was calculated. All peaks were then compared and only those showing a >2-fold in expression with P values <0.05 in MEAM1 and <0.01 in MED were selected for analysis. Further sifting of the data was done to keep only proteins that had at least one unique peptide sequence identified. All three final lists of proteins, from the three technical replicates, were then compared and only proteins that appeared in at least two of the three were kept (see table S1, supplementary data).

Funding

This work was funded by a Binational Agricultural Research and Development (BARD) travel grant to Adi Kliot.

Authors' contributions

AK- Investigation, Formal analysis, Validation, Visualization, Writing- original draft, Funding Acquisition

MM- Formal analysis

RJ- Formal analysis

GL- Resources

SK- Resources

HC- Supervision, Writing- review & editing

MH- Methodology, Resources, Validation, Data Curation, Funding Acquisition, Writing- review & editing

MG- Funding Acquisition, Conceptualization, Supervision, Writing- review & editing

References

1. Oliveira, M., T. Henneberry, and P. Anderson, *History, current status, and collaborative research projects for Bemisia tabaci.* Crop protection, 2001. **20**(9): p. 709-723.
2. Jones, D.R., *Plant viruses transmitted by whiteflies.* European Journal of Plant Pathology, 2003. **109**(3): p. 195-219.
3. Navas-Castillo, J., E. Fiallo-Olivé, and S. Sánchez-Campos, *Emerging virus diseases transmitted by whiteflies.* Annual Review of Phytopathology, 2011. **49**: p. 219-248.
4. Ghosh, S., et al., *Transmission of a new polerovirus infecting pepper by the whitefly Bemisia tabaci.* Journal of virology, 2019: p. JVI. 00488-19.
5. De Barro, P.J., et al., *Bemisia tabaci: a statement of species status.* Annual review of entomology, 2011. **56**: p. 1-19.
6. Liu, S.-s., J. Colvin, and P.J. De Barro, *Species Concepts as Applied to the Whitefly Bemisia tabaci Systematics: How Many Species Are There?* Journal of Integrative Agriculture, 2012. **11**(2): p. 176-186.
7. Brown, J., D. Frohlich, and R. Rosell, *The sweetpotato or silverleaf whiteflies: biotypes of Bemisia tabaci or a species complex?* Annual review of entomology, 1995. **40**(1): p. 511-534.
8. Chiel, E., et al., *Biotype-dependent secondary symbiont communities in sympatric populations of Bemisia tabaci.* Bulletin of Entomological Research, 2007. **97**(04): p. 407-413.
9. Chen, W., et al., *The draft genome of whitefly Bemisia tabaci MEAM1, a global crop pest, provides novel insights into virus transmission, host adaptation, and insecticide resistance.* BMC biology, 2016. **14**(1): p. 1-15.
10. Xie, W., et al., *Genome sequencing of the sweetpotato whitefly Bemisia tabaci MED/Q.* GigaScience, 2017. **6**(5): p. gix018.
11. Franco, C.F., et al., *Monitoring virus-like particle and viral protein production by intact cell MALDI-TOF mass spectrometry.* Talanta, 2010. **80**(4): p. 1561-1568.
12. Tsai, J.-M., et al., *Genomic and proteomic analysis of thirty-nine structural proteins of shrimp white spot syndrome virus.* Journal of virology, 2004. **78**(20): p. 11360-11370.
13. Cilia, M., et al., *Discovery and targeted LC-MS/MS of purified polerovirus reveals differences in the virus-host interactome associated with altered aphid transmission.* 2012.
14. Papura, D., et al., *Two-dimensional electrophoresis of proteins discriminates aphid clones of Sitobion avenae differing in BYDV-PAV transmission.* Archives of virology, 2002. **147**(10): p. 1881-1898.
15. Mishra, M., et al., *Proteome analysis of Bemisia tabaci suggests specific targets for RNAi mediated control.* Journal of proteomics, 2016. **132**: p. 93-102.
16. Yang, N., et al., *Transcriptomic and Proteomic Responses of Sweetpotato Whitefly, Bemisia tabaci, to Thiamethoxam.* PLoS ONE, 2013. **8**(5): p. e61820.
17. Gottlieb, Y., et al., *The transmission efficiency of tomato yellow leaf curl virus by the whitefly Bemisia tabaci is correlated with the presence of a specific symbiotic bacterium species.* Journal of virology, 2010. **84**(18): p. 9310-9317.

18. Rispe, C., et al., *Large gene family expansion and variable selective pressures for cathepsin B in aphids.* Molecular biology and evolution, 2008. **25**(1): p. 5-17.
19. Götz, M., et al., *Implication of Bemisia tabaci heat shock protein 70 in begomovirus-whitefly interactions.* Journal of virology, 2012. **86**(24): p. 13241-13252.
20. Bao, Y.-Y., et al., *De novo intestine-specific transcriptome of the brown planthopper Nilaparvata lugens revealed potential functions in digestion, detoxification and immune response.* Genomics, 2012. **99**(4): p. 256-264.
21. Wang, L., et al., *A new shrimp peritrophin-like gene from Exopalaemon carinicauda involved in white spot syndrome virus (WSSV) infection.* Fish & Shellfish Immunology, 2013. **35**(3): p. 840-846.
22. Leevers, S.J., B. Vanhaesebroeck, and M.D. Waterfield, *Signalling through phosphoinositide 3-kinases: the lipids take centre stage.* Current opinion in cell biology, 1999. **11**(2): p. 219-225.
23. Le Blanc, I., et al., *Endosome-to-cytosol transport of viral nucleocapsids.* Nature cell biology, 2005. **7**(7): p. 653-664.
24. Sookruksawong, S., et al., *RNA-Seq analysis reveals genes associated with resistance to Taura syndrome virus (TSV) in the Pacific white shrimp Litopenaeus vannamei.* Developmental & Comparative Immunology, 2013. **41**(4): p. 523-533.
25. Berois, M., J. Romero-Severson, and D. Severson, *RNAi knock-downs support roles for the mucin-like (AeIMUC1) gene and short-chain dehydrogenase/reductase (SDR) gene in Aedes aegypti susceptibility to Plasmodium gallinaceum.* Medical and veterinary entomology, 2012. **26**(1): p. 112-115.
26. Rohrmann, G.F., *The baculovirus replication cycle: Effects on cells and insects.* 2013.
27. Rider, M.A., et al., *Quantitative Proteomic Analysis of the Anopheles gambiae (Diptera: Culicidae) Midgut Infected With O'nyong–Nyong Virus.* Journal of medical entomology, 2013. **50**(5): p. 1077-1088.
28. Bramhill, D., *Bacterial cell division.* Annual review of cell and developmental biology, 1997. **13**(1): p. 395-424.
29. Yang, X., et al., *Coupling genetics and proteomics to identify aphid proteins associated with vector-specific transmission of polerovirus (Luteoviridae).* Journal of virology, 2008. **82**(1): p. 291-299.
30. Tamborindeguy, C., et al., *Genomic and proteomic analysis of Schizaphis graminum reveals cyclophilin proteins are involved in the transmission of Cereal yellow dwarf virus.* PloS one, 2013. **8**(8): p. e71620.
31. Kanakala, S. and M. Ghanim, *Implication of the whitefly Bemisia tabaci cyclophilin B protein in the transmission of Tomato yellow leaf curl virus.* Frontiers in plant science, 2016. **7**: p. 1702.
32. Kanakala, S., et al., *Plant-Mediated Silencing of the Whitefly Bemisia tabaci Cyclophilin B and Heat Shock Protein 70 impairs insect development and virus transmission.* Frontiers in physiology, 2019. **10**: p. 557.
33. Ntwasa, M., A. Goto, and S. Kurata, *Coleopteran antimicrobial peptides: prospects for clinical applications.* International journal of microbiology, 2012. **2012**.
34. Balmand, S., et al., *Antimicrobial peptides keep insect endosymbionts under control.* Science, 2011. **334**(6054): p. 362-365.
35. Zhang, S., et al., *Hemagglutinating and antibacterial activities of vitellogenin.* Fish & shellfish immunology, 2005. **19**(1): p. 93-95.
36. Wei, J., et al., *Vector development and vitellogenin determine the transovarial transmission of begomoviruses.* Proceedings of the National Academy of Sciences, 2017. **114**(26): p. 6746-6751.

37. Rubinstein, G. and H. Czosnek, *Long-term association of tomato yellow leaf curl virus with its whitefly vector Bemisia tabaci: effect on the insect transmission capacity, longevity and fecundity.* Journal of General Virology, 1997. **78**(10): p. 2683-2689.
38. Cohen, S. and F. Nitzany, *Transmission and host range of the tomato yellow leaf curl virus.* Phytopathology, 1966. **56**(10): p. 1127-1131.
39. Ghanim, M., et al., *Evidence for Transovarial Transmission of Tomato Yellow Leaf Curl Virus by Its Vector, the WhiteflyBemisia tabaci.* Virology, 1998. **240**(2): p. 295-303.
40. Levy, F., et al., *Peptidomic and proteomic analyses of the systemic immune response of Drosophila.* Biochimie, 2004. **86**(9): p. 607-616.
41. Reumer, A., et al., *Unraveling the protective effect of a Drosophila phosphatidylethanolamine-binding protein upon bacterial infection by means of proteomics.* Developmental & Comparative Immunology, 2009. **33**(11): p. 1186-1195.
42. Ott, D.E., et al., *Actin-binding cellular proteins inside human immunodeficiency virus type 1.* Virology, 2000. **266**(1): p. 42-51.
43. Pan, L., et al., *Differential efficiency of a begomovirus to cross the midgut of different species of whiteflies results in variation of virus transmission by the vectors.* Science China Life Sciences, 2018. **61**(10): p. 1254-1265.
44. Brumin, M., et al., *Levels of the endosymbiont Rickettsia in the whitefly Bemisia tabaci are influenced by the expression of vitellogenin.* Insect Molecular Biology, 2020. **29**(2): p. 241-255.
45. Pinheiro, P.V., et al., *Is there a role for symbiotic bacteria in plant virus transmission by insects?* Current Opinion in Insect Science, 2015. **8**: p. 69-78.
46. Kliot, A., et al., *Implication of the Bacterial Endosymbiont Rickettsia spp. in Interactions of the Whitefly Bemisia tabaci with Tomato yellow leaf curl virus.* Journal of virology, 2014. **88**(10): p. 5652-5660.
47. Kliot, A., et al., *Combined infection with Tomato yellow leaf curl virus and Rickettsia influences fecundity, attraction to infected plants and expression of immunity-related genes in the whitefly Bemisia tabaci.* Journal of General Virology, 2019. **100**(4): p. 721-731.
48. Cilia, M., et al., *A comparison of protein extraction methods suitable for gel-based proteomic studies of aphid proteins.* Journal of biomolecular techniques: JBT, 2009. **20**(4): p. 201.
49. Perez-Riverol, Y., et al., *The PRIDE database and related tools and resources in 2019: improving support for quantification data.* Nucleic acids research, 2019. **47**(D1): p. D442-D450.
50. Käll, L., et al., *Semi-supervised learning for peptide identification from shotgun proteomics datasets.* Nature methods, 2007. **4**(11): p. 923-925.
51. Spivak, M., et al., *Improvements to the percolator algorithm for peptide identification from shotgun proteomics data sets.* Journal of proteome research, 2009. **8**(7): p. 3737-3745.

Figure Legends

Figure 1. TYLCV Transmission abilities of MED (A) and MEAM1 (B) species populations used in this study. MspRQ and OberRB are the populations with the highest transmission efficiency in each species. Numbers above columns represent the number of plants tested for virus transmission with whiteflies from each population.

Figure 2. Top 40 differentially abundant proteins in OberRB. The 20 proteins with significantly low abundance and the 20 proteins with significantly high abundance in the MEAM1 efficient vector population compared to all other MEAM1 populations.

Figure 3. Top 40 differentially abundant proteins in MspRQ. The 20 proteins with significantly low abundance and the 20 proteins with significantly high abundance in the MED efficient vector population compared to the other MED populations.

Figure 4. *Rickettsia* proteins found at high quantities in both MEAM1 and MED efficient vector populations. Common bacterial proteins significantly abundant in MEAM1 (black) and MED (white) efficient vectors.

Figure 5. Additional symbiont (*Rickettsia* and *Hamiltonella)* proteins with high and low abundance in the MEAM1 efficient vector population.

Figure S1. Peptide PCAs for selected populations. Two PCA analyses for three randomly selected populations (of 9 in the experiment- three in A and three in B). Data for each PCA consisted of quantification of all peptides found in all three biological replicates performed for each population and all three technical replicates performed per biological replicate.

Figure S2. Proteins of differential expression common to both efficient vector populations show opposite abundances. Common proteins with significantly different quantities in MEAM1 (dark gray) and MED (light gray) efficient vectors.

Figure S3. *B. tabaci* complete Vitellogenin amino acids sequence. Highlighted are peptides identified to be of high abundance in the MEAM1 efficient TYLCV vector compared to the rest of the MEAM1 populations. Formatted are the peptides identified to be of low abundance in the MED efficient vector compared to the rest of MED populations.

Figure S4. Total Ion Current (TICs) of three selected runs. The TIC is the summed intensity of all ions (all m/zs) for the entire LCMS run. A and B are duplicate injections of the same sample and C is a biological replicate injection. TIC shows high reproducibility and a good spread across the gradient. The intensity of the second biological replicate (C), seems lower than the first biological replicate (A and B), which could be due to slightly lower concentration. As the data was normalized for comparison this isn't a problem.

Figure S5. Base Peak Chromatograms of three selected runs. base peak chromatograms for the same three runs as in figure S3. A and B are duplicate injections of the same sample and C is a biological replicate injection. The base peak chromatogram is the intensity of the most intense m/z peak during each scan. The base peaks are very reproducible between analytical replicates (A and B) and between biological replicates (A, B compared to C). The retention times are reproducible, many abundant peaks to within one minute.

Table 1. Populations collected and used in this study.

Symbiont Legend: P- *Portiera*, H- *Hamiltonella*, A- *Arsenophonus*, W- *Wolbachia*, R- *Rickettsia*, C- *Cardinium*.

	**Symbiont populations composition**						
**population name**	**P**	**H**	**A**	**W**	**R**	**C**	**collection site**
**MED populations**							
Q-AWR	+		+	+	+		Ayalon valley, Israel
fluf	+		+	+	+		Israel
Zadar	+	+	+	+			Zadar, Croatia
Q'-HC	+	+				+	Croatia
MspRQ	+		+	+	+		Israel
**MEAM1 populations**							
Ayalon	+	+			+		Ayalon Valley, Israel
MspRB	+	+					Israel
Tamra	+	+			+		Tamra, Israel
ObeRB	+	+			+		Israel

Figure 1

Click here to access/download;Figure;Figure 1.tif

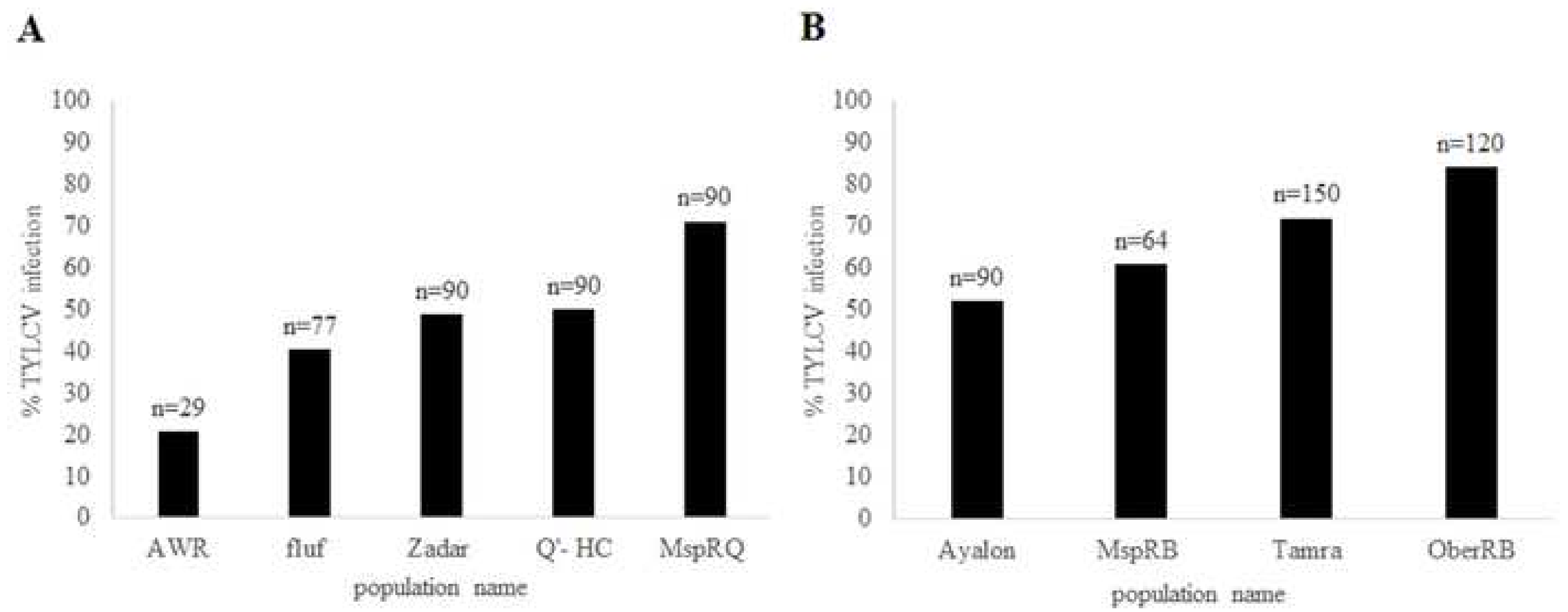

Figure 2

Click here to access/download;Figure;Figure 2.tif

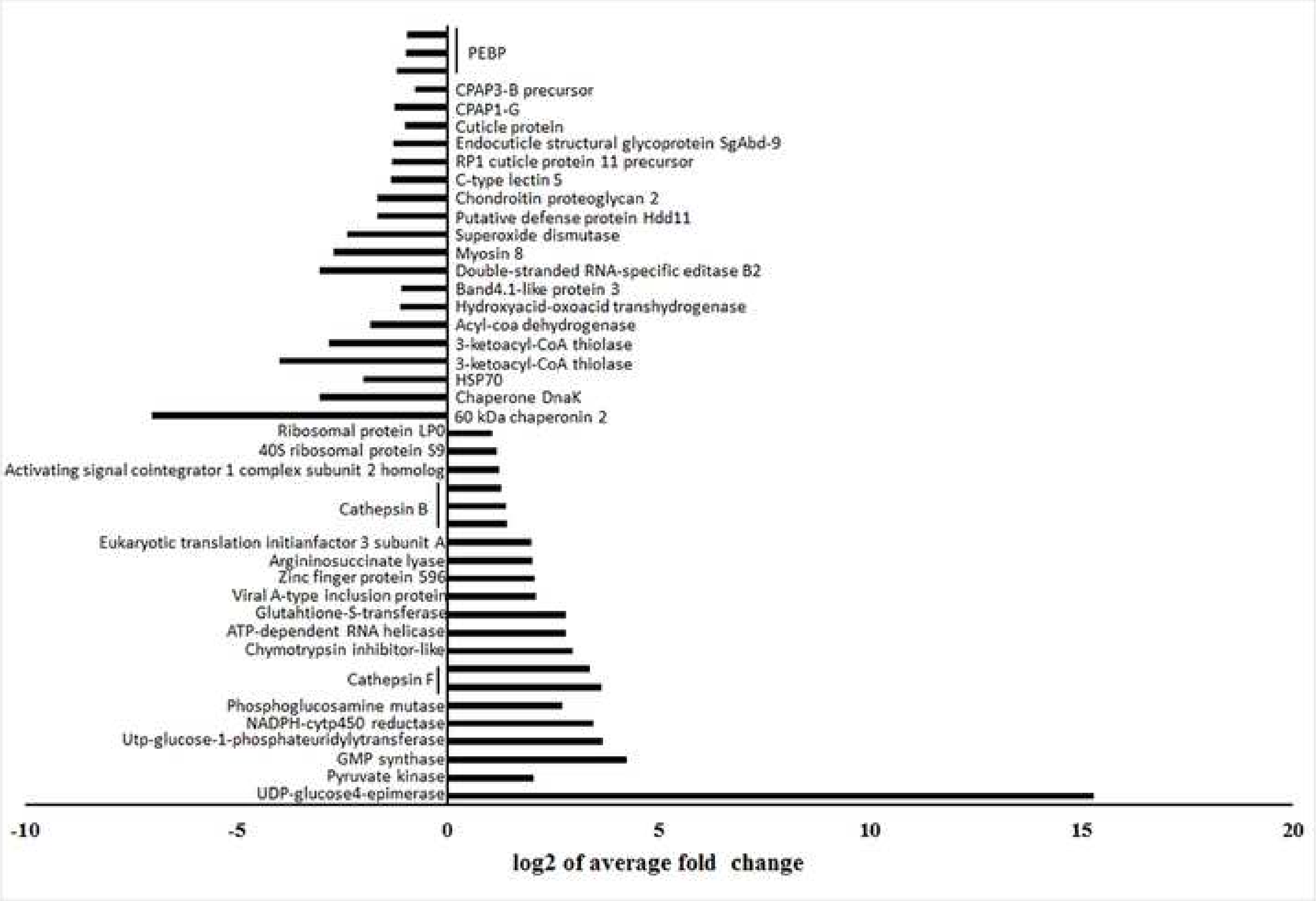

Figure 3

Click here to access/download;Figure;Figure 3.tif

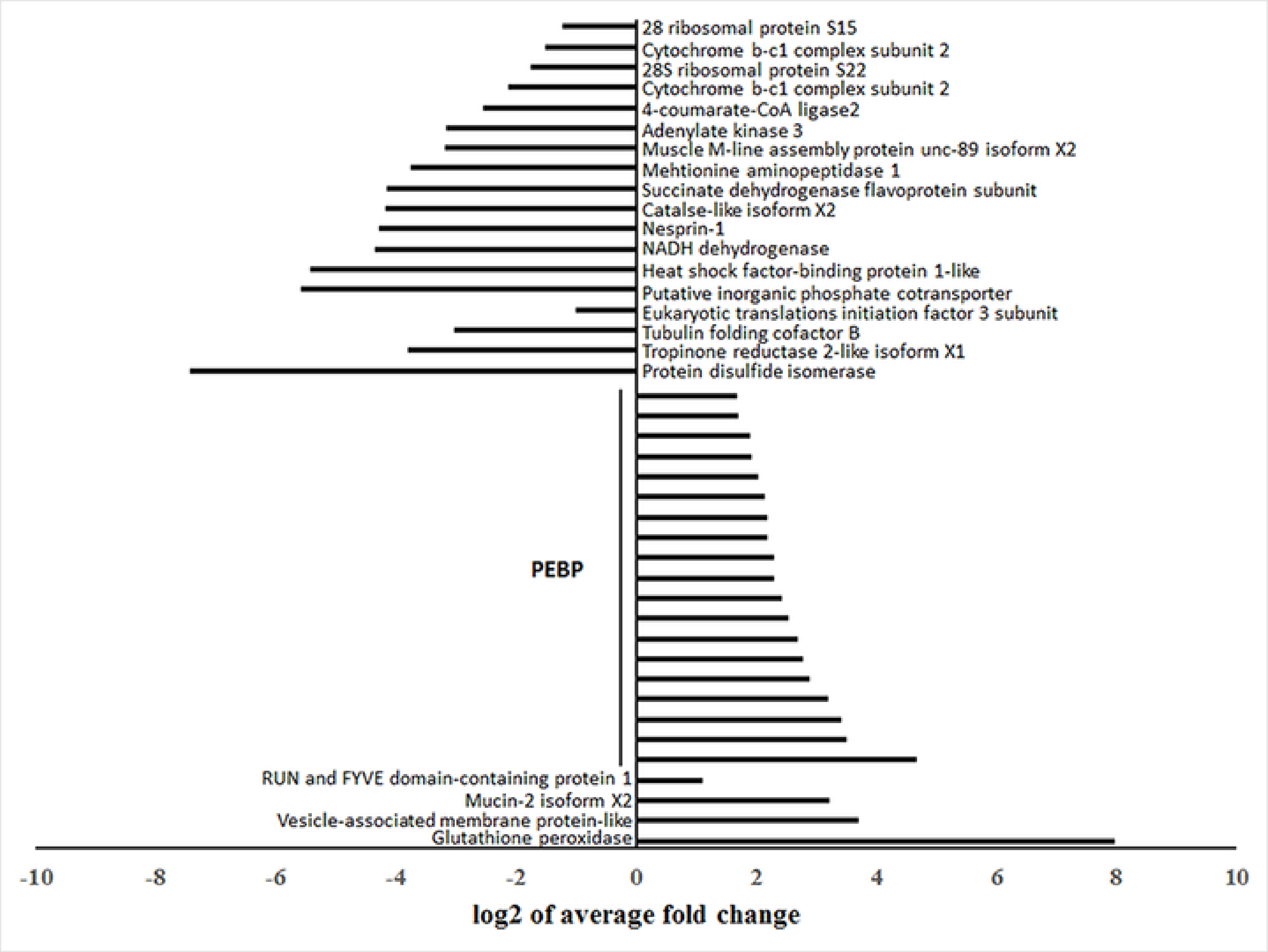

Figure 4

Click here to access/download;Figure;Figure 4.tif

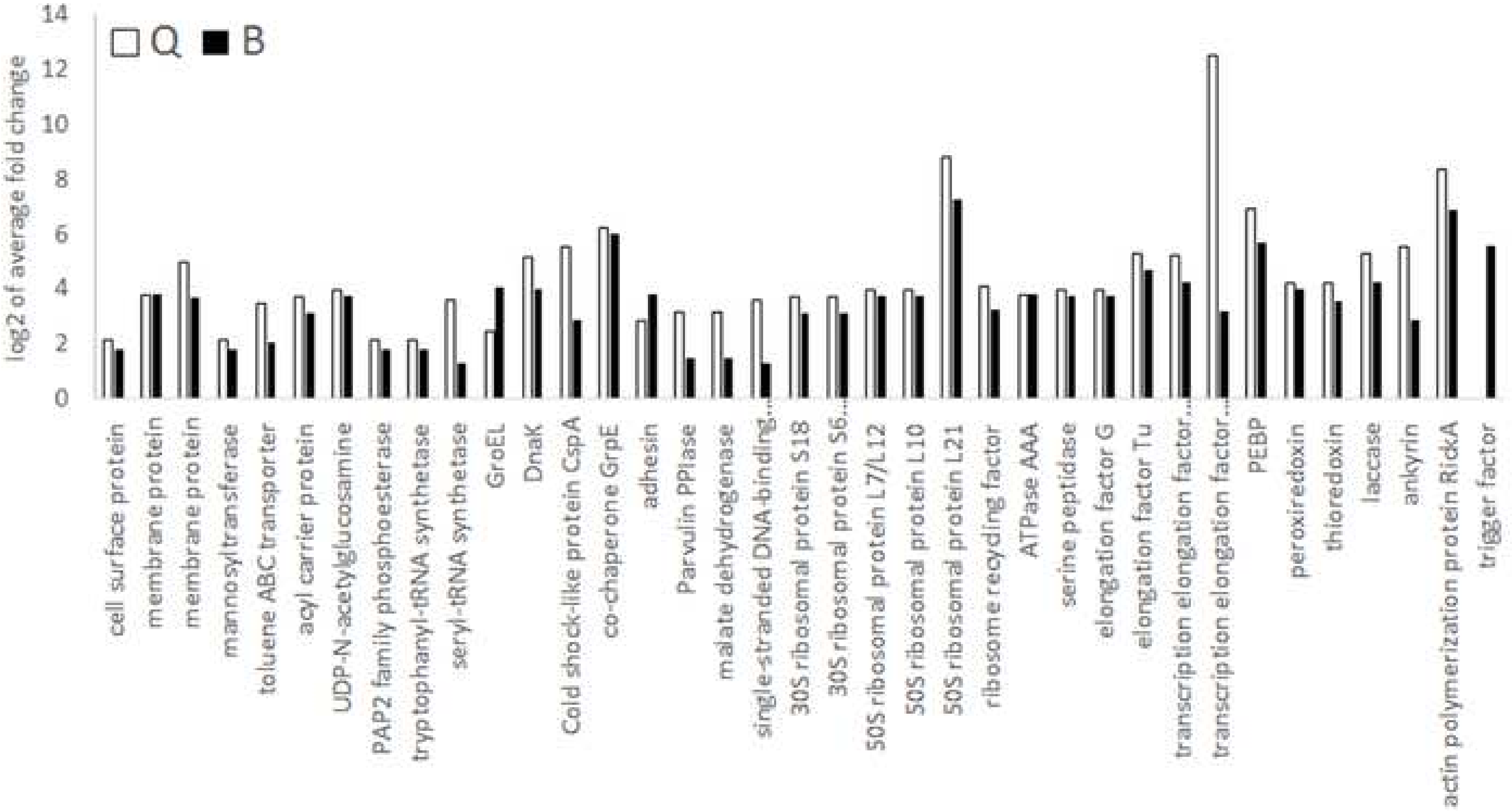

Figure 5

Click here to access/download;Figure;Figure 5.tif

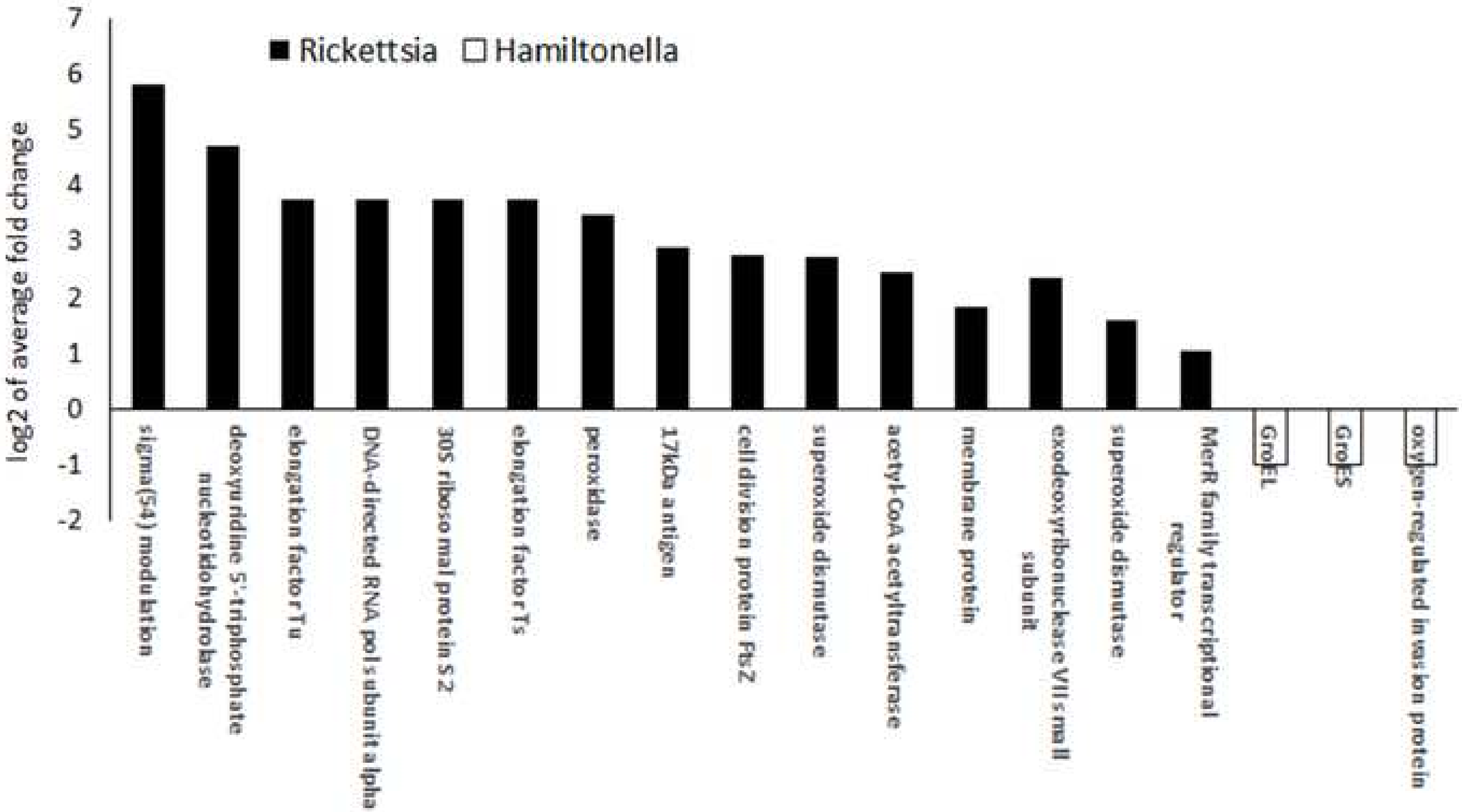

Click here to access/download
Supplementary Material
Supplementary figures and legends.pdf

Supplementary TableS1

Click here to access/download

Supplementary Material

supplementary table 1- all DAPs.xlsx

Personal Cover Click here to access/download;Personal Cover;cover letter.pdf

מדינת ישראל / משרד החקלאות ופיתוח הכפר
State of Israel / Ministry of Agriculture and Rural Development

Agricultural Research Organization
The Volcani Center
Institute of Plant Protection
Department of Entomology

Prof. Murad Ghanim

מינהל המחקר החקלאי
מרכז וולקני
המכון להגנת הצומח
המחלקה לאנטומולוגיה

פרופ' מוראד גאנם

18.7.2020

Dr. Nicole Nogoy,

Gigascience,

Dear Dr. Nogoy,

We are submitting a revision to our manuscript entitled "A proteomic approach reveals possible molecular mechanisms and roles for endosymbiotic bacteria in begomovirus transmission by whiteflies". The manuscript was revised based on the reviewers comments and was reformatted as a Data Note as suggested in your decision letter.

The authors of this manuscript declare no competing interests, and confirm that all all authors have approved the manuscript for submission, and that the content of the manuscript has not been published, or submitted for publication elsewhere.

We hope our manuscript will now be found suitable for publication in GigaScience,

Sincerely,
Murad Ghanim

P.O. Box 6, Bet-Dagan 50250 ISRAEL – 50250 ת"ד 6, בית דגן
Tel/Fax +972-3-9683911 טל'/פקס:
WWW: http://www.agri.gov.il/en/people/759.aspx
