# Supplementary material for: A proteomic approach reveals possible molecular mechanisms and roles for endosymbiotic bacteria in begomovirus transmission by whiteflies
Source: Gigascience. 2020 Nov 13;9(11):giaa124. doi: 10.1093/gigascience/giaa124 (PMC7662926; doi:10.1093/gigascience/giaa124)
Supplement: giaa124_Supplemental_Files [file giaa124_supplemental_files.zip › Supplementary figures and legends-ak101120.pdf]

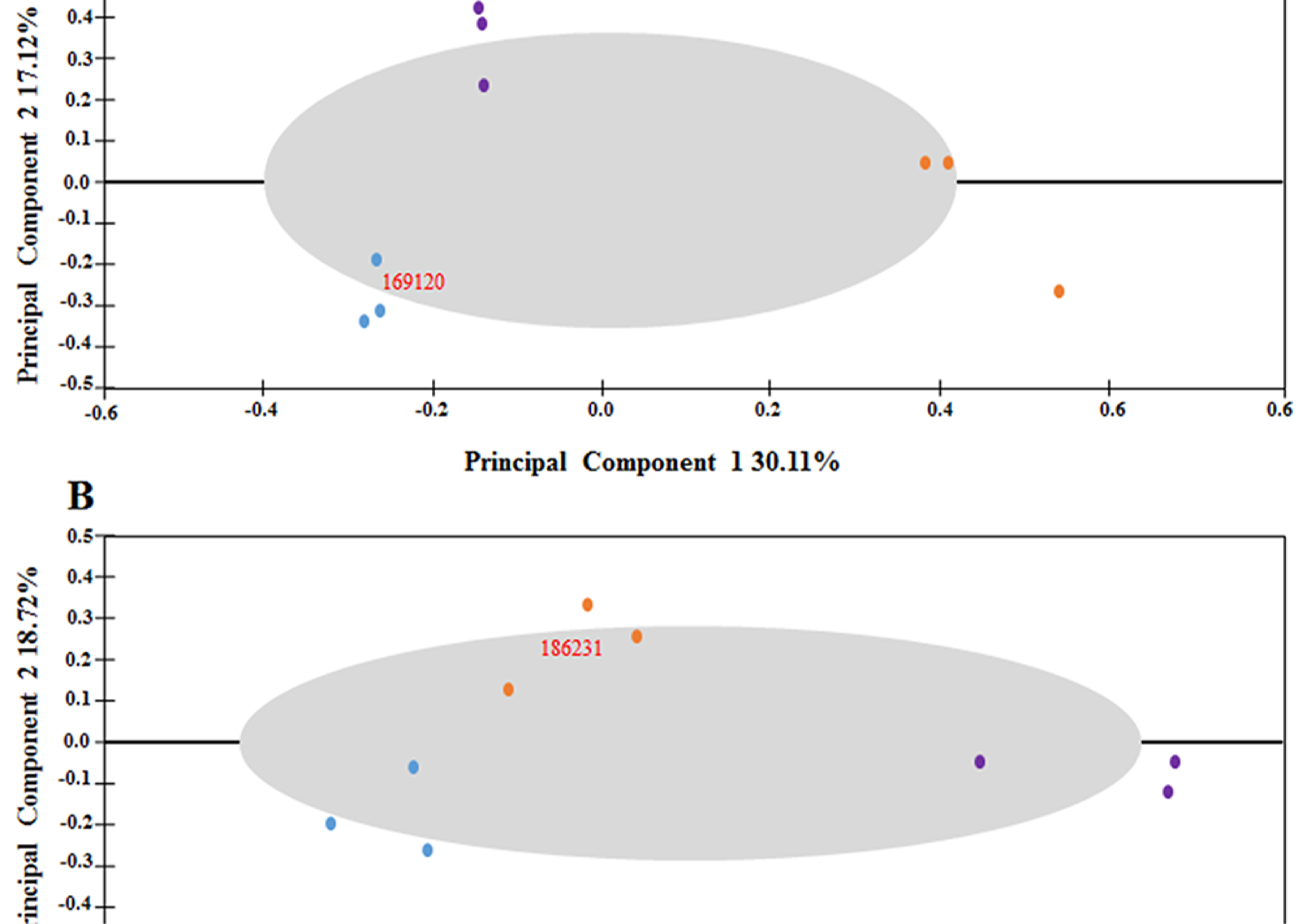

Principal Component 2 17.12%
0.4
0.3
0.2
0.1
0.0
-0.1
-0.2
-0.3
-0.4
-0.5
169120
-0.6
-0.4
-0.2
0.0
0.2
0.4
0.6
0.6
Principal Component 1 30.11%
B
Principal Component 2 18.72%
0.5
0.4
0.3
0.2
0.1
0.0
-0.1
-0.2
-0.3
-0.4
186231

mwtpvllcllvaaanaqygwkngnlykyevngrtltalnqvadqyagvlfranfivqplssdrlsaqiqnaeiaqvhte
lpagyeshipssqlnyknmplseqpfeinlkqgvvsnlrvnknvsdwelniikavvsqiqvdtqgqnlkksshnqlp
kenkpygvyktmedsvtgecetlydvsplpeitlqtrpwlvpfpklrengqvidivkatnyskceersayhfgitgltn
wkpanqmgqflsrsninrviisgnvkqyhiqssvstnkivispqmyesqkgmvvsvmnlxlasfhqsngsprmvs
narkinnlvydynaaspnayaqhynnngassssssssssssdssssssssssssssssssssssssseedqyyrrhnnnnr
nnnnnnnnnnnrnnnhnnnnnandnnnsannsnnnnsnnsannnnnnnknnnrnndenvsrsrsrrdisqyky
nsfennnnndnedeknnsgrnghnghngnnghngnnghnghnghngnngqnnsngssssssssssssssgsssse
ennsrynngkfasfarhngsgsssssssspdssdsssssssssssssssssssssssssssednssfgssvsssseedyeprps
tykapqtpffpyfignygnsiqsakqvngvalarklaqeiaeelndprqitqkstlakfnmlveelrtldakqmeqasqe
lhfnsaqasshsrqdalkslawksfcdalveagtgpaflqiqkiiehqqvsdaeaarmisrlpvtarfpdkeymnsffnf
vrsnnvqhqnqlnetallafaelcrkadvnarnahnyypvhvygrvlpehakavahqylpyyeqnlkravangdsrk
iqayiraignfahpkilevfepylegkvpisnfqrtvmvlslnelarvypnlarnvlfkiyqntqenqgvrvaavflifgtn
psaqtlqrmaqftnedqdqqvnaavksalenaakahsesrqelaqaaqsaiallspktyglqyskkwlrdyivkeenla
yrvsadmiqsedslipnqvyvalhrylggfaqrvasframtssasdlvekiqeqftngeeyqqqsemnqqfsaeqifr
qfnikpdypqeveallqytvfgakrwaffdeeffnqiprrlndapskvqngqsfnstkfyndislslafptatglpfsytlk
vptlvqaggevqarvqghnsnnnnnnnnlf**ripeavnvtaeieivyatelkselgfvtpfnheryvaglaknifvni**
pvkvaanvdiantkvefymkpmnnqneqkifhygsypytaiqnifdfrplqenentkyifanenknkfekv
ygeektgfafrcqykgdqqsfqfadfynfakrndffsaaffpwaektiqynnfdvyydparsaaksakfalny
ankyankennnegnsnnhnnnqndavpssyqpdneqrmnqfasraqsgvqsanidvigisaqffgqknad
yvatlayarspvaekarflfyagannannnknkvavdataympnvplvnaaqafnadansrfyanvksgenldnga
qfqfqanmkqsqefrdyfrqsqmykqcsqqmeqgeymmpacrnatvvanrlneahfsinfdkvsdavknytyqa
fayarhlgyqyqsenfgnpngqhnkidgyfkfspkfdfaqfyfnapsvaasfknvpvhqyvadffaphpvysgfdrl
mqdtfqakyqaacvadkmhattfdnktyplhmqqnnwyvlmtyvnrnnyynnqynsyfqgnnknqysyrdyn
qkrfyttvyarqnsngqkelkivlnngeyeifmqpassraglhssnsgknnaaikvfinkqeqqfndkqftdfhghng
kiyaqfyalpdgairffapqsglqaiydgarikiqaanqyrgavrgmcgtysnqyaddftapqncvyknpedfaavya
vidssspsqvksqkeraqqnfcahknnqfgnyvsrsdagygykynnndkyyesaykntkyydpsnsqynkyykn
nkyakdqdssyssssssssdsssssssdsssssssssssssssssdssssqsndnnrnnnnrnnnnrqqqqqpqqqq
qpqqqqqqqpqqqpqqqpqqqqqqqqqprsqlrtekpkrtlypqtvpchqprrrhvlhhqrypnlqipsqarw
krqeng

Figure S2

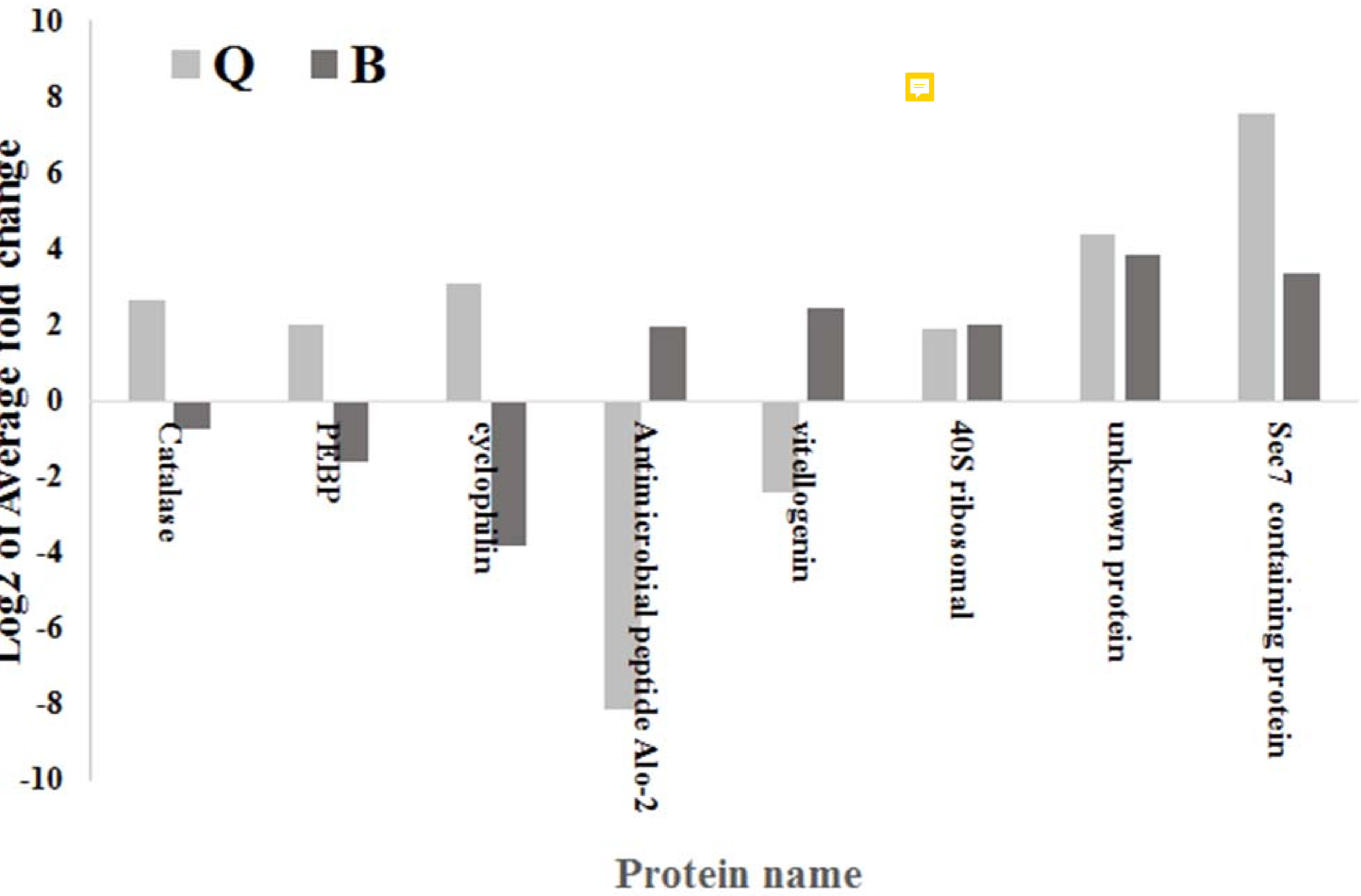

Q
B
Log2 of Average fold change
10
8
6
4
2
0
-2
-4
-6
-8
-10
Catalase
PEBP
cyclophilin
Antimicrobial peptide Alo-2
vitellogenin
40S ribosomal
unknown protein
Sec7 containing protein
Protein name

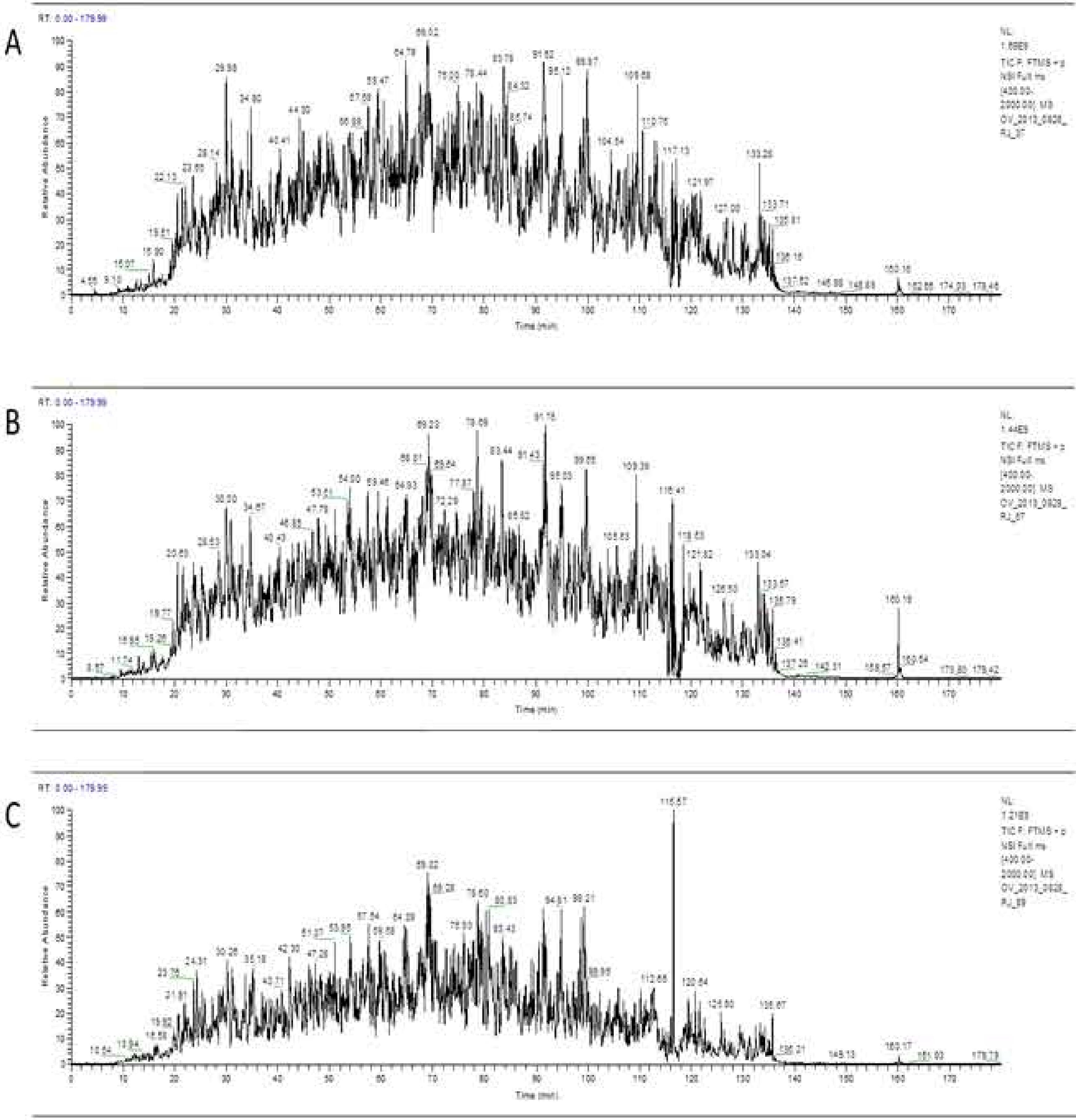
A
B
C
Relative Abundance
Time (min)
TIC F: FTMS + p NSI Full ms [400.00-2000.00] MS

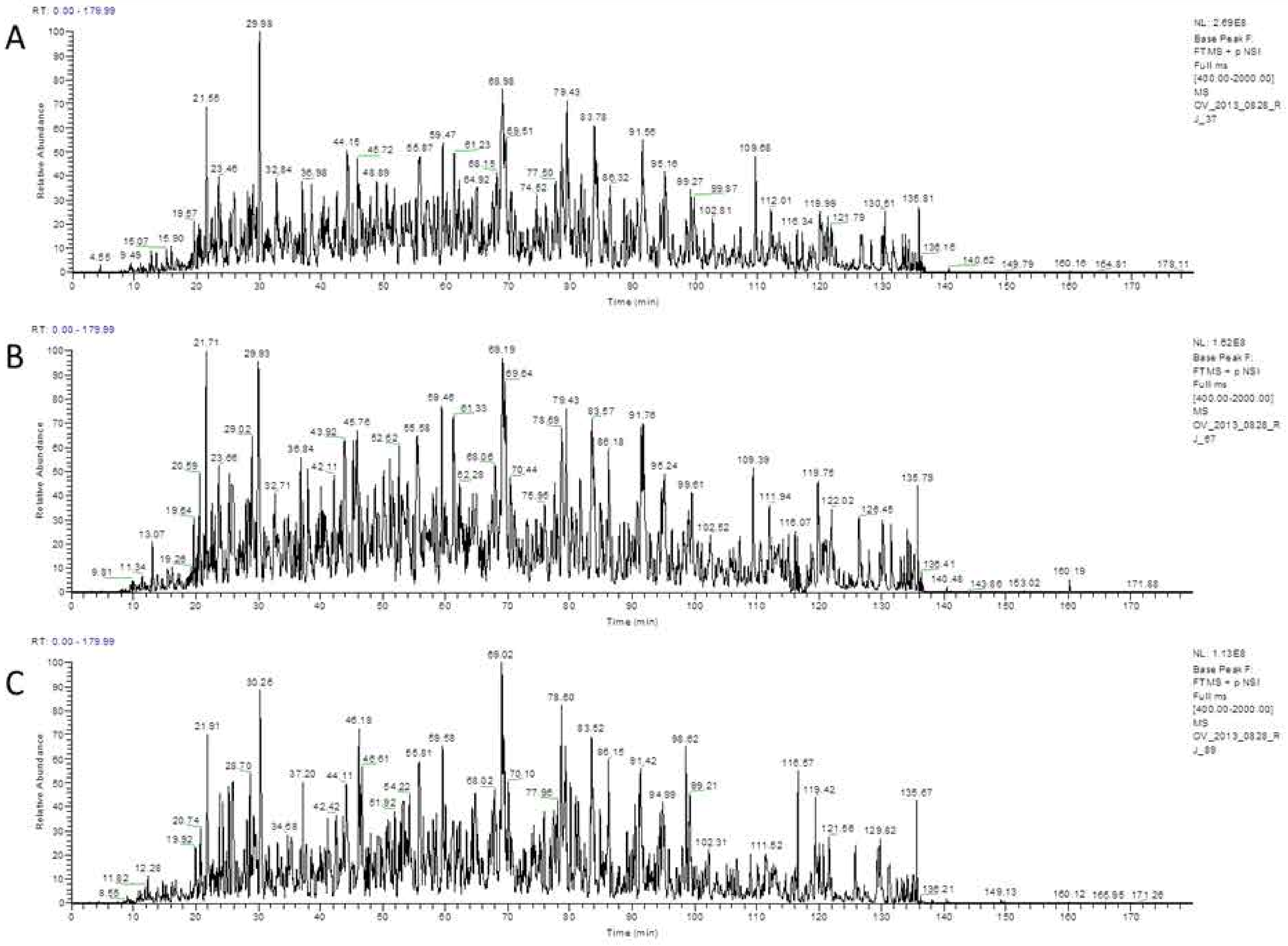

A
RT: 0.00 - 179.99
NL: 2.69E8
Base Peak F: FTMS + p NSI Full ms [400.00-2000.00] MS OV_2013_08.28_RJ_37
Relative Abundance
Time (min)
B
RT: 0.00 - 179.99
NL: 1.52E8
Base Peak F: FTMS + p NSI Full ms [400.00-2000.00] MS OV_2013_08.28_RJ_67
Relative Abundance
Time (min)
C
RT: 0.00 - 179.99
NL: 1.13E8
Base Peak F: FTMS + p NSI Full ms [400.00-2000.00] MS OV_2013_08.28_RJ_89
Relative Abundance
Time (min)

Supplementary figures legends.

Figure S1. Peptide PCAs for selected populations. Two PCA analyses for three randomly selected populations (of 9 in the experiment- three in A and three in B). Data for each PCA consisted of quantification of all peptides found in all three biological replicates performed for each population and all three technical replicates performed per biological replicate.

Figure S2. *B. tabaci* complete Vitellogenin amino acids sequence. Highlighted are peptides identified to be of high abundance in the MEAM1 efficient TYLCV vector compared to the rest of the MEAM1 populations. Formatted are the peptides identified to be of low abundance in the MED efficient vector compared to the rest of MED populations.

Figure S3. Proteins of differential expression common to both efficient vector populations show opposite abundances. Common proteins with significantly different quantities in MEAM1 (dark gray) and MED (light gray) efficient vectors.

Figure S4. Total Ion Current (TICs) of three selected runs. The TIC is the summed intensity of all ions (all m/zs) for the entire LCMS run. A and B are duplicate injections of the same sample and C is a biological replicate injection. TIC shows high reproducibility and a good spread across the gradient. The intensity of the second biological replicate (C), seems lower than the first biological replicate (A and B), which could be due to slightly lower concentration. As the data was normalized for comparison this isn't a problem.

Figure S5. Base Peak Chromatograms of three selected runs. base peak chromatograms for the same three runs as in figure S3. A and B are duplicate

injections of the same sample and C is a biological replicate injection. The base peak chromatogram is the intensity of the most intense m/z peak during each scan. The base peaks are very reproducible between analytical replicates (A and B) and between biological replicates (A, B compared to C). The retention times are reproducible, many abundant peaks to within one minute.
