# Supplementary material for: A proteomic approach reveals possible molecular mechanisms and roles for endosymbiotic bacteria in begomovirus transmission by whiteflies
Source: Gigascience. 2020 Nov 13;9(11):giaa124. doi: 10.1093/gigascience/giaa124 (PMC7662926; doi:10.1093/gigascience/giaa124)
Supplement: giaa124_Response_to_Reviewer_Comments_Revision_1 [file giaa124_response_to_reviewer_comments_revision_1.pdf]

Author's Response To Reviewer Comments

Close

We thank the reviewers and editor for their valuable and constructive comments. We have modified the manuscript based on the comments raised, converted the paper to a note as instructed. The list of comments that were addressed are given below (C) and our responses are given underneath (A):
Response to reviewer 1:
C: The result section under abstract- "A protein database, over-expressed and down downregulated proteins from efficient vector populations from two different B. tabaci species are provided."
There should be uniformity in the words used in the sentence, either up/down or over/under. The use of word "expressed" or "regulated" can't be used directly in proteomics study without supported by some biological results. As far as selection of words to explain identified proteins, I would suggest use Increased/ decreased abundant proteins.
A: We have changed all references to protein quantities in the manuscript to "higher / lower abundance".

C: "Data is available through ProteomeXchange with identifier PXD016964…" - I was not able to see the dataset as reviewer.
A: We apologise for issues with PRIDE. Those have now been fixed and the data is now available.

C: I suggest author should also provide increased/ decreased abundant proteins lists from nine whiteflies populations from both Middle East Asia Minor I (MEAM1 formerly known as B biotype) and Mediterranean (MED formerly known as Q biotype) species as supplementary files.

A: We have changed all references to protein quantities in the manuscript to "higher / lower abundance".

C: We then compared the expression levels of all peptides and proteins in order to identify proteins that are differentially expressed in each…"
Same as above, expression is not the correct term to use. You can write- We then compared the abundance levels of all peptides and proteins in order to identify proteins that are differentially abundant in each… Please change accordingly throughout in manuscript.

A: We have changed all references to protein quantities in the manuscript to "higher / lower abundance".

C: same as above. Please change to Proteins differentially abundant… and in subsequent places.

A: We have changed all references to protein quantities in the manuscript to "higher / lower abundance".

C: A spelling mistakes "specie"

A: mistake has been fixed.

C: The sentence "The over expression of these two proteins in the efficient-vector populations confirmed the validity of our approach regarding the MED efficient-vector population."
Author is referring two proteins, what are those proteins?

A: The sentence is referring to the low abundances of HSP70 and cyclophilin in MED. The reviewer is right to point out this sentence that was moved out of context in our discussion. The discussion has now been extensively edited and the above sentence has been deleted.

C: Protein were extracted as described in [58]- Wrong reference, it should be 57. Please check other

references.
A: Reference was indeed wrong and has been updated.

C: "Pellets were saved and washed 3 times with cold acetone, dried and re-suspended in 8M urea in 100mM ABC.
Protein was quantified using a Bradford assay, protein integrity was examined by running 5μg from each sample on 1D gel …"
Author used Bradford assay of proteins dissolved in 8M urea in 100mM ABC. As it is known that Urea is incompatible with Bradford assay. So, I will suggest them to add gel pictures in manuscript.

A: Unfortunately, we could not get hold of all gel images in time for this deadline, however, we added figures S4 and S5 to the manuscript supplementary data in which we show the total ion current and base peak chromatograms for 3 samples; 2 technical replicates of the same biological replicate and one more biological replicate from the same population. These show high reproducibility among samples and good spread across the gradient.

C: please add final concentration or total amount of trypsin added to reaction mixture.

A: From the updated method section: "Samples were then diluted to ~1M urea with 100 mM ABC and Trypsin was added in a 1:50 ratio (Trypsin:protein)."

C: How was the elution schedule done? Was it a continuous gradient or stepwise?

A: From the updated method section: "Peptides were eluted off the column using a gradient of 2-35% acetonitrile in 0.1% formic acid over 120 minutes, followed by 35-60% acetonitrile over 10 minutes at a flow rate of 250 nl/min."

C: Which was the data acquisition ion mode (positive?) set in the MS?

A: From the updated method section: "The resolution for MS was 60,000 at m/z 400 covering the m/z range of 400-2000. MS/MS spectra were acquired using a linear ion trap that provided unit resolution. The automatic gain control targets for MS in the orbitrap was 1e6, whereas for MS/MS it was 8000, and the maximum fill times were 20 and 80 msec, respectively. The MS/MS spectra were acquired using an isolation width of 2 m/z and a normalized collision energy (NCE) of 35."

C: Which was the charge range of the selected peptides for MS?

A: From the updated method section: "The precursor ion threshold intensity was set to 5000 in order to trigger an MS/MS acquisition. Furthermore, MS/MS acquisitions were prevented for precursor charge states of 1, or if the charge state could not be discerned from the MS spectrum. Dynamic exclusion (including all isotope peaks) was set for 20 seconds. "

C: What is the model name of MS system?

A: From the updated method section: "All mass spectrometry was performed on a LTQ-Orbitrap-Velos (Thermo Fisher Scientific)."

C: How did author calculated abundance? please highlight.

A: From the updated method section: "Mascot files were then loaded into the Progenesis QI program (Nonlinear) and aligned to a randomly selected reference run."
"Average normalized abundance was calculated for each protein, based on protein features without conflict only, and fold change between the highest and lowest values was calculated. All peaks were then compared and only those showing a >2-fold in expression with P values <0.05 in MEAM1 and <0.01 in MED were selected for analysis."
To clarify- per set of biological replicates, a random selected sample (selected based on run score calculated by Progenesis QI program, usually the run with the highest score on that set) was chosen as a reference run. Peak intensities for all proteins were calculated based on the reference run.

C: Describe about P H A W R C labelling in footnote.

A: From the updated table legend: “Symbiont Legend: P- Portiera, H- Hamiltonella, A- Arsenophonus, W- Wolbachia, R- Rickettsia, C- Cardinium.”

Response to reviewer 2:
C: The English written needs to be deeply improved.

A: the manuscript has undergone extensive editing and English proofing.

C: The discussion is no good. It seems only a description. The manuscript is too descriptive. Proteomics works are not like that!

A: We thank the reviewer for pointing this out and we agree
The discussion has been modified to be less ‘listy’, however many of the data we found important is still included. We tried editing and turning the discussion into a more ;story telling ‘ section drawing conclusions from the data in the results.

C: The authors have to make sense in all results they found out. In my opinion the discussion should be rewritten in a better way.

A: As written above, we rewrote and edited our discussion and hope it is now more fitting.

C: I suggest some additional experiments of qPCR to try to corroborates both RNA expression and protein data.

A: This manuscript is now submitted as a data note and the editor mentioned that there is no need for qPCR verification experiments in a data note. We also think that qPCRs will indicate the RNA levels and might not reflect the proteomic profile obtained in our results.

C: The conclusion is a merely short communication of the results. It has to be done again.

A: The conclusions have also been rewritten.

C: Where is the protein table? I need to all identified proteins.

A: We apologize for excluding this in the first submission. Supplementary table 1 is now added and includes all proteins that showed significantly different abundances in all 3 sets of biological replicates.

C: The MS runs is badly described! There are many question about MS analysis? How many runs were done for each sample? How many biological samples were used? Three? Five? How many peptides were used to identify a protein?

A: Three biological replicates were run per population and three technical runs for each biological replicate. We have rewritten the MS runs part of the methods and revised other parts of the methods’ section as well.
From the updated methods’ section: “Further sifting of the data was done to keep only proteins that had at least one unique peptide sequence identified.”

Close
