# Supplementary material for: A proteomic approach reveals possible molecular mechanisms and roles for endosymbiotic bacteria in begomovirus transmission by whiteflies
Source: Gigascience. 2020 Nov 13;9(11):giaa124. doi: 10.1093/gigascience/giaa124 (PMC7662926; doi:10.1093/gigascience/giaa124)
Supplement: giaa124_Response_to_Reviewer_Comments_Revision_2 [file giaa124_response_to_reviewer_comments_revision_2.pdf]

Author's Response To Reviewer Comments

Close

Two comments were raised by reviewer # 2 in lines 140 and 160 and both comments were addressed as suggested. Several other changes were made throughout the manuscript as suggested by the reviewer.

Close
