# Supplementary material for: A proteomic approach reveals possible molecular mechanisms and roles for endosymbiotic bacteria in begomovirus transmission by whiteflies
Source: Gigascience. 2020 Nov 13;9(11):giaa124. doi: 10.1093/gigascience/giaa124 (PMC7662926; doi:10.1093/gigascience/giaa124)
Supplement: giaa124_Response_to_Reviewer_Comments_Revision_3 [file giaa124_response_to_reviewer_comments_revision_3.pdf]

Author's Response To Reviewer Comments

Close

08.10.2020
Dr. Nicole Nogoy,
Gigascience,

Dear Dr. Nogoy,

We are submitting a revision to our manuscript entitled "A proteomic approach reveals possible molecular mechanisms and roles for endosymbiotic bacteria in begomovirus transmission by whiteflies". The few remaining additions requested by the editor were addressed and the manuscript modified accordingly.

The authors of this manuscript declare no competing interests, and confirm that all all authors have approved the manuscript for submission, and that the content of the manuscript has not been published, or submitted for publication elsewhere.

We hope our manuscript will now be found acceptable for publication in GigaScience,

Sincerely,
Murad Ghanim

Close
